# Discovery of Coumarins from *Zanthoxylum dimorphophyllum* var. *spinifolium*as and Their Potential against Rheumatoid Arthritis

**DOI:** 10.3390/molecules29184395

**Published:** 2024-09-16

**Authors:** Caixia Du, Xingyu Li, Junlei Chen, Lili Luo, Chunmao Yuan, Jue Yang, Xiaojiang Hao, Wei Gu

**Affiliations:** 1 State Key Laboratory of Functions and Applications of Medicinal Plants, Guizhou Medical University, Guiyang 550014, China; 2 Natural Products Research Center of Guizhou Province, Guiyang 550014, China; 3 School of Basic Medicine, Guizhou Medical University, Guiyang 550014, China; 4 Bijie Medical College, Bijie 551700, China; 5 College of Science, Yunnan Agricultural University, Kunming 650201, China; 6 State Key Laboratory of Phytochemistry and Plant Resources in West China, Kunming Institute of Botany, Chinese Academy of Sciences, Kunming 650201, China

**Keywords:** *Zanthoxylum dimorphophyllum* var. *spinifolium*, coumarins, anti-rheumatoid arthritis, network pharmacology

## Abstract

In the present study, a series of coumarins, including eight undescribed bis-isoprenylated ones Spinifoliumin A-H, were isolated and identified from the aerial parts of *Zanthoxylum dimorphophyllum* var. *spinifolium* (ZDS), a plant revered in traditional Chinese medicine, particularly for treating rheumatoid arthritis (RA). The structures of the compounds were elucidated using 1D and 2D NMR spectroscopy, complemented by ECD, [Rh_2_(OCOCF_3_)_4_]-induced ECD, Mo_2_(OAc)_4_ induced ECD, IR, and HR-ESI-MS mass spectrometry. A network pharmacology approach allowed for predicting their anti-RA mechanisms and identifying the MAPK and PI3K-Akt signaling pathways, with EGFR as a critical gene target. A CCK-8 method was used to evaluate the inhibition activities on HFLS-RA cells of these compounds. The results demonstrated that Spinifoliumin A, B, and D-H are effective at preventing the abnormal proliferation of LPS-induced HFLS-RA cells. The results showed that compounds Spinifoliumin A, D, and G can significantly suppress the levels of IL-1β, IL-6, and TNF-α. Moreover, molecular docking methods were utilized to confirm the high affinity between Spinifoliumin A, D, and G and EGFR, SRC, and JUN, which were consistent with the results of network pharmacology. This study provides basic scientific evidence to support ZDS’s traditional use and potential clinical application.

## 1. Introduction

Rheumatoid arthritis (RA) is a systemic inflammatory autoimmune disease mainly affecting the surrounding joints. RA without systemic treatment can persist for many years, eventually leading to joint deformities and loss of function [1]. According to epidemiological investigations, the prevalence rate in China is 0.34–0.45%. Most people are middle-aged and elderly patients. Due to the rapid decline of hormone levels, the incidence rate in women is 2–3 times higher than men [2]. Without adequate treatment, the disease can lead to progressive joint destruction and deformity, causing long-term disability, chronic pain, and premature death [3]. During the pathological process of RA, fibroblast-like synoviocytes (FLSs) exhibit tumor-like abnormal proliferation and invade cartilage and bone, producing a variety of cytokines, leading to chronic inflammation of cartilage and bone [4]. RA-FLS is an important effector cell in the course of RA. The over-proliferation of RA-FLSs will lead to damage of joints and chronic persistent inflammation [5,6,7]. Therefore, inhibiting the proliferation of RA-FLSs is an important therapeutic strategy in the clinical treatment of RA [8]. Current studies have shown that the pathogenesis of RA is related to multiple mechanisms, such as infection, heredity, immune mechanism disorders, and metabolic abnormalities [4]. However, it has not yet been definitively concluded, and there is still a lack of effective preventive and therapeutic drugs [4].

*Zanthoxylum dimorphophyllum* var. *spinifolium* Rehder & E. H. Wilson (ZDS) is an evergreen shrub or small arbor belonging to the Rutaceae family. ZDS is mainly distributed in the Shanxi, Hubei, Guizhou, and Sichuan provinces of China [9]. Traditionally, its root is commonly used for treating rheumatoid arthritis (RA), traumatic injury, cough, and deworming [10,11]. Its fruit peel is also used as a seasoning and preservative in many places. Previously research has been reported on coumarins [9,12], alkaloids [12], and lignins [13] from ZDS; among them, coumarins are the main components. Nevertheless, as a commonly used folk medicine for the treatment of rheumatoid arthritis, the effect components on RA from ZDS have not been reported yet.

Coumarins are important natural compounds with 1, 2 benzopyrone structures [14]. As the third major category of chemical components in the *Zanthoxylum* genus, most of the coumarins reported from the *Zanthoxylum* primarily belong to simple coumarins and furanocoumarins. In addition, the former are excellent lead compounds with low molecular weights, simple structures, and easy modification, exhibiting broad application prospects [15]. Especially, numerous reports have demonstrated that some coumarins have excellent anti-rheumatoid arthritis effects [16,17,18,19], In addition, several studies have shown that coumarins in *Zanthoxylum* plants have anti-inflammatory activity [20,21]. Based on the above reasons, we speculate that coumarin components may be the main active ingredients of ZDS in exerting anti-RA effects. Therefore, this study intends to focus on exploring the coumarin compounds in ZDS and their effects on RA.

**Figure 1:**
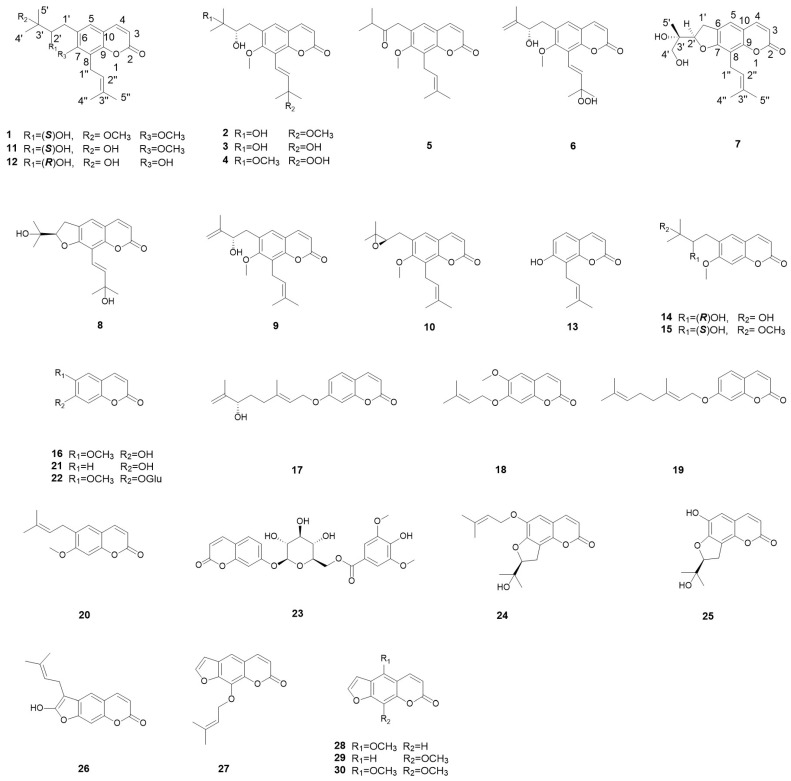
Chemical structures of compounds **1**–**30**.

In the present work, eight undescribed coumarins along with twenty-two known ones were isolated and identified from ZDS (Figure 1). Furthermore, the mechanism and target of the isolated compounds related to RA were predicted through network pharmacology methods. The effects of new coumarins from ZDS on human rheumatoid fibroblast-like synoviocyte RA (HFLS-RA) cells in vitro were investigated. The levels of related pro-inflammatory cytokines were analyzed and discussed.

## 2. Results

### 2.1. Structural Elucidation of Eight New Compounds

**Figure 2:**
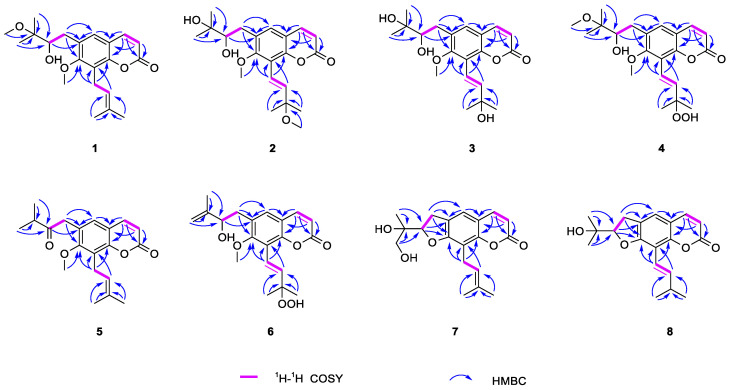
The key HMBC and ^1^H-^1^H COSY correlations of compounds **1**–**8**.

**Figure 3:**
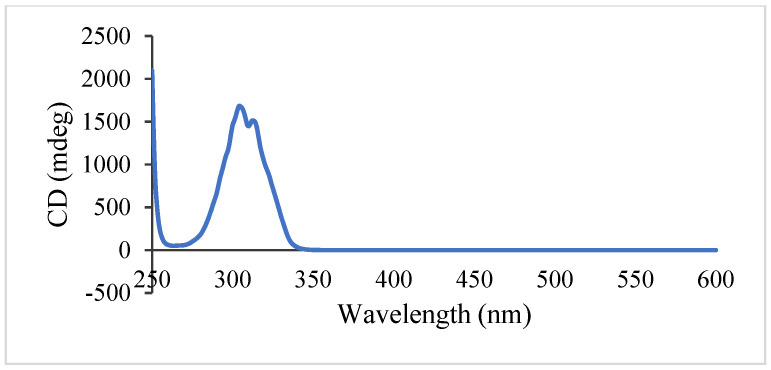
ECD curve of the Rh_2_(OCOCF_3_)_4_ complex of compound **1**.

Spinifoliumin A (**1**) was obtained as a yellow amorphous solid. HRESIMS determined the molecular formula C_21_H_28_O_5_ at *m*/*z* 383.1840 [M + Na]^+^ (calcd. for C_21_H_28_O_5_Na *m*/*z* 383.1830), requiring eight degrees of unsaturation. The IR spectrum showed the existence of a hydroxyl group (3482 cm^−1^), carbonyl (1731 cm^−1^), aromatic ring (1606 cm^−1^), and olefinic bond (2977 cm^−1^). Analysis of the NMR data of **1** (Table 1 and Table 2) suggested that the structure of **1** was the methoxyl analog of 6-(3′-methyl-2′,3′-dihydroxylbutyl)-7-methoxyl-8-(3″-methyl-2″-butenyl)-2H-1-benzopyran-2-one (**11**) [9]. The key HMBC correlations (Figure 2) between H-OCH_3_ (*δ*_H_ 3.30) and C-3′ (*δ*_C_ 77.5) demonstrated that the methoxyl group was connected to C-3′ in **1**. Subsequently, the absolute configuration of 2′ for **1** was determined by an [Rh_2_(OCOCF_3_)_4_]-induced circular dichroism experiment. According to the bulkiness rule [22], a positive Cotton effect at approximately 350 nm (Figure 3) elucidated the 2′S configuration in **1**. Thus, the structure of **1** was identified as 6-(2′S-hydroxy-3′-methoxyl-3′-methylbutyl)-7-methoxyl-8-(3″-methyl-2″-butenyl)-2H-1-benzopyr-an-2-one (Figure 1) and was given the name Spinifoliumin A.

Spinifoliumin B (**2**) was isolated as a yellow amorphous solid. HRESIMS deduced the molecular formula C_21_H_28_O_6_ at *m*/*z* 399.1789 [M + Na]^+^ (calcd. for C_21_H_28_O_6_Na *m*/*z* 399.1778). The IR spectrum showed the existence of a hydroxyl group (3420 cm^−1^), carbonyl (1705 cm^−1^), aromatic ring (1631 cm^−1^), and olefinic bond (2976 cm^−1^). The ^1^H-NMR spectrum (Table 1) displayed five unsaturated protons at *δ*_H_ 6.34 (d, *J* = 9.6 Hz, H-3), 7.63 (d, *J* = 9.6 Hz, H-4), 7.28 (s, H-5), 6.77 (d, *J* = 16.8 Hz, H-1″), and 6.71 (d, *J* = 16.8 Hz, H-2″), two methoxy protons at *δ*_H_ 3.78 (s, 7-OCH_3_) and 3.28 (s, 3″-OCH_3_), and four methyls at *δ*_H_ 1.33 (s, H-4′), 1.30 (s, H-5′), 1.43 (s, H-4″), and 1.43 (s, H-5″). Simultaneously, the ^13^C-NMR and HMQC spectra (Table 2) revealed twenty-one carbon resonances consisting of a coumarin skeleton (*δ*_C_ 162.8, 115.1, 146.1, 130.5, 132.3, 161.3, 119.6, 152.4, 116.8), two substitutions for the isoamyl group (*δ*_C_ 32.9, 79.4, 73.8, 25.9, 24.9) and isopentenyl group (*δ*_C_ 119.3, 143.4, 77.2, 26.2, 26.1), and two methoxyl groups (*δ*_C_ 51.0 and 61.6). Analysis of the data of **2** (Table 1 and Table 2) suggested that the plane structure of **2** was similar to 6-(3′-methyl-2′,3′-dihydroxylbutyl)-7-methoxyl-8-(3″-methyl-2″-butenyl)-2H-1-benzopyran-2-one (**11**) [9], except for the position of the trans-olefinic bond in the C-8 side chain. ^1^H-^1^H COSY correlation (Figure 2) of H-1″ (*δ*_H_ 6.71)/H-2″(*δ*_H_ 6.77), combined with the key HMBC correlations from H-2″ (*δ*_H_ 6.77) to C-8 (*δ*_C_ 119.6) and C-1″ (*δ*_C_ 119.3), H-4″ (*δ*_H_ 1.43) and H-5″ (*δ*_H_ 1.43) to C-2″ (*δ*_C_ 143.4) and C-3″ (*δ*_C_ 77.2), indicated that the substitution in **2** was 3″-methyl-3″-methoxyl-1″-butenyl group (Figure 2). In addition, the position of the methoxyl group was determined via the HMBC correlation from H-OCH_3_ (*δ*_H_ 3.30) to C-3″ (*δ*_C_ 77.2). Compounds **2** and **1** have the same chiral centers, and the optical rotation of **2** ([*α*]_25_D +81.8 (c = 1.0, MeOH)) was found to be consistent with that of **1**. Thus, the structure of **2** was determined to be 6-(2′S,3′-dihydroxyl-3′-methylbutyl)-7-methoxyl-8-(3″-methyl-3″-methoxyl-1″*E*-butenyl)-2H-1-benzopyran-2-on (Figure 1).

Spinifoliumin C (**3**) was a yellow amorphous solid. HRESIMS deduced the molecular formula C_20_H_26_O_6_ at *m*/*z* 385.1616 [M + Na]^+^ (calcd. for C_20_H_26_O_6_Na *m*/*z* 385.1622), which was 14 mass units less than 2. The 5.3 ppm downshift of C-3″ (*δ*_C_ 71.9) in the ^13^C NMR spectra of **3** indicated that the methoxyl in 2 was replaced by a hydroxyl. The key HMBC correlations from H-2″ (*δ*_H_ 6.92) to C-8 (*δ*_C_ 119.9) and C-1″ (*δ*_C_ 116.0), H-4″ (*δ*_H_ 1.43), and H-5″ (*δ*_H_ 1.44) to C-2″ (*δ*_C_ 146.2) and C-3″ (*δ*_C_ 71.9) indicated that the substitution of C-8 in **3** was the 3″-methyl-3″-hydroxyl-1″-butenyl group (Figure 2). The absolute configuration of compound **3** can be determined by the consistent binding of its chiral center with compound **1** based on its specific rotation. Finally, the absolute stereochemistry of **3** was confirmed to be 2′S. Thus, the structure of 3 was determined to be 6-(2′S,3′-dihydroxyl-3′-methylbutyl)-7-methoxyl-8-(3″-methyl-3″-hydroxyl-1″*E*-butenyl)-2H-1-benzopyran-2-one (Figure 1).

Spinifoliumin D (**4**) was isolated as a yellow amorphous solid. HRESIMS deduced the molecular formula C_21_H_28_O_6_ at *m*/*z* 415.1719 [M + Na]^+^ (calcd. for C_21_H_28_O_6_Na *m*/*z* 415.1727). The IR spectrum showed the existence of a hydroxyl group (3421 cm^−1^), carbonyl (1719 cm^−1^), aromatic ring (1602 cm^−1^), and olefinic bond (2976 cm^−1^). The increased 16 mass units of the molecular weight (*m*/*z* 415.1719, [M + Na]^+^) observed in HRESIMS and the 10.7 ppm downshift of C-3″ (*δ*_C_ 82.5) in the ^13^C NMR spectra of **4** indicated that the hydroxyl in 2 was replaced by a hydroperoxyl in **4**. The key HMBC correlations between H-OCH_3_ (*δ*_H_ 3.32) and C-3′ (*δ*_C_ 73.8) demonstrated that the methoxyl group was connected to C-3′ in **4**. Finally, the absolute configuration of 2′S for **4** was determined by the similarity of the optical rotation with **1**. Thus, the structure of **4** was determined to be 6-(2′S,3′-dihydroxyl-3′-methoxyl-3′-methylbutyl)-7-methoxyl-8-(3″-methyl-3″-hydroperoxyl-1″*E*-butenyl)-2H-1-benzopyran -2-one (Figure 1).

Spinifoliumin E (**5**), a yellow amorphous solid, gave the molecular formula of C_20_H_24_O_4_Na^+^ by the positive HRESIMS signal at *m*/*z* 351.1572 (calcd. for C_20_H_24_O_4_Na, 351.1567), which was 18 mass units less than 11. The IR spectrum showed the existence of carbonyl (1721 cm^−1^), an aromatic ring (1611 cm^−1^), and an olefinic bond (2965 cm^−1^). The 1D NMR spectral data of **5** (Table 2 and Table 3) exhibited similarities with those of 6-(3′-methyl-2′,3′-dihydroxylbutyl)-7-methoxyl-8-(3″-methyl-2″-butenyl)-2H-1-benzopyran-2-one (**11**) [9]. The primary structural difference between the two was the replacement of the 3′-methyl-2′-oxobutyl moiety at C-6 in **5**. This modification was evidenced by the ^1^H-^1^H COSY correlation of H-4′ (*δ*_H_ 1.19)/H-3′ (*δ*_H_ 2.78) and H-5′ (*δ*_H_ 1.19), combined with the key HMBC correlations from H-1′ (*δ*_H_ 3.84) to C-5 (*δ*_C_ 127.8), C-6 (*δ*_C_ 125.1), C-7 (*δ*_C_ 159.6), and C-2′ (*δ*_C_ 211.7) and from H-4′ (*δ*_H_ 1.19) and H-5′ (*δ*_H_ 1.19) to C-2′ (*δ*_C_ 211.7) and C-3′ (*δ*_C_ 40.8). Therefore, the structure of **5** was established as 6-(3′-methyl-2′-oxobutyl)-7-methoxy-8-(3″-methyl-3″-methoxyl-1″-butenyl)-2H-1-benzopyran-2-one (Figure 1).

Spinifoliumin F (**6**) was obtained as a yellow amorphous powder. Its molecular formula was determined as C_20_H_24_O_6_ by the positive HRESIMS peak at *m*/*z* 3833.1459 [M + Na]^+^ (calcd. for 383.1465). Compared to the ^1^H NMR and ^13^C NMR spectra (Table 2 and Table 3) of **6** and **4**, they have the same substituent group in C-8. The key HMBC correlations from H-2′ (*δ*_H_ 4.43) to C-6 (*δ*_C_ 129.3) and C-1′ (*δ*_C_ 36.2), H-4′ (*δ*_H_ 5.07 and 4.90), and H-5′ (*δ*_H_ 1.88) to C-2′ (*δ*_C_ 74.9) and C-3′ (*δ*_C_ 147.0) indicated that the substitution in C-6 was the 2′-hydroxyl-3′-methyl-3′-butenyl group of **6** (Figure 2). Finally, the absolute configuration of 2′S for **6** was confirmed by the similar optical rotation of [α]_25_D +58.8 (c = 1.0, MeOH) with **1**. Thus, the structure of **6** was determined to be 6-(2′S-dihydroxyl-3′-methyl-3′-butenyl)-7-methoxyl-8-(3″-methyl-3″-hydroperoxyl-1″*E*-butenyl)-2H-1-benzopyran-2-one (Figure 1).

**Figure 4:**
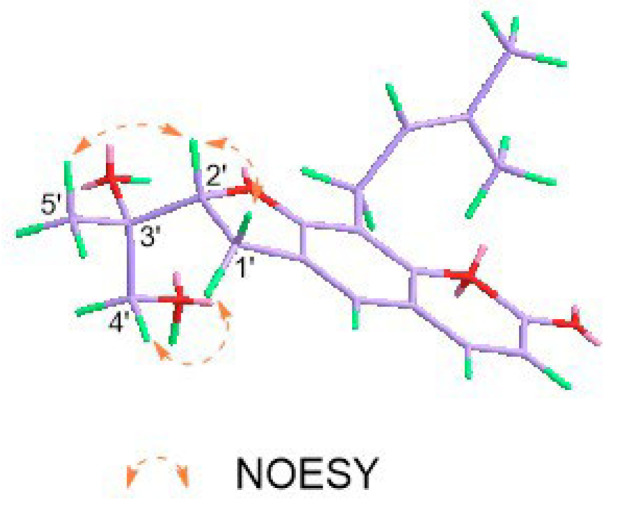
NOESY correlations of compound **7**.

**Figure 5:**
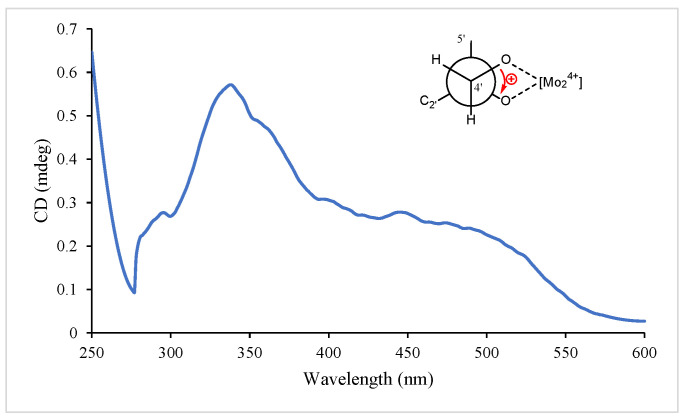
CD spectrum of **7** in DMSO containing Mo_2_(OAc)_4_ with the inherent CDs subtracted.

**Figure 6:**
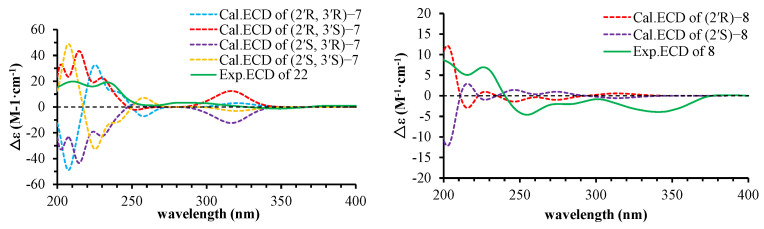
Experimental and calculated ECD spectra of compounds **7** and **8**.

Spinifoliumin G (**7**) was obtained as a yellow amorphous powder. The molecular formula was confirmed as C_19_H_22_O_5_ by the HRESIMS peak at *m*/*z* 353.1369 [M + Na]^+^ (calcd. for 353.1359). The IR spectrum showed the existence of a hydroxyl group (3418 cm^−1^), carbonyl (1705 cm^−1^), aromatic ring (1579 cm^−1^), and olefinic bond (2915 cm^−1^). The ^1^H-NMR spectrum (Table 3) displayed four unsaturated protons at *δ*_H_ 6.15 (d, *J* = 9.6 Hz, H-3), 7.80 (d, *J* = 9.6 Hz, H-4), 7.47 (s, H-5), and 5.28 (t, *J* = 7.2 Hz, H-2″) and three methyls at *δ*_H_ 1.20 (s, H-5′), 1.83 (s, H-4″), and 1.67 (s, H-5″). Simultaneously, the ^13^C-NMR and HMQC spectra (Table 2) revealed nineteen carbon resonances consisting of three methyls, three methylenes, five methyines, and eight quaternary carbons. Further analysis based on the literature data found that compound **7** was very similar to that of 2′,3′-dihydro-2′-(4′-hydroxymethyl-ethyl)-8-(3″-methyl-2″-buten-1″-yl)-2H-1-benzopyran-2-one [9], except for the side moiety at C-3′. The HMBC correlations between H-4′ (*δ*_H_ 7.80), C-3′ (*δ*_C_ 111.6), and C-2′ (*δ*_C_ 164.0) proved the exiting of hydroxymethyl moiety at C-3′. In addition, the NOESY cross-peaks of H-2′ (*δ*_H_ 4.94), /H-1′α (*δ*_H_ 3.24), and H-5′ (*δ*_H_ 1.2) indicated that these protons were α oriented (Figure 4). The absolute configuration of 3′S was determined by the positive Cotton effect at 310–340 nm in the Mo_2_(OAc)_4_-induced ECD spectrum (Figure 5) [23]. 2′R for **7** was further determined by comparing the experimental and calculated ECD data (Figure 6). Consequently, the structure of **7** was established as 2′,3′-dihydro-2′-(4′-hydroxymethyl-ethyl)-8-(3″-methyl-2″-buten-1″-yl)-2H-1-benzopyran-2-one (Figure 1).

Spinifoliumin H (**8**) was obtained as a yellow amorphous powder. Its molecular formula, C_19_H_22_O_4_, was deduced by the HRESIMS peak at *m*/*z* 353.1353 [M + Na]^+^(calcd. for 353.1359). The IR spectrum showed the existence of a hydroxyl group (3422 cm^−1^), carbonyl (1705 cm^−1^), aromatic ring (1611 cm^−1^), and olefinic bond (2972 cm^−1^). The ^1^H-NMR spectrum (Table 3) displayed five unsaturated protons at *δ*_H_ 6.24 (d, *J* = 9.6 Hz, H-3), 7.85 (d, *J* = 9.6 Hz, H-4), 7.3 (d, s, H-5), 7.0 (d, *J* = 16.8 Hz, H-1″), and 7.08 (d, *J* = 16.8 Hz, H-2″) and four methyls at *δ*_H_ 1.36 (s, H-4′), 1.30 (s, H-5′), 1.44 (s, H-4″), and 1.44 (s, H-5″). The ^13^C NMR data (Table 2) exhibited 19 carbon resonances, including a furocoumarin skeleton (*δ*_C_ 163.6, 111.9, 146.8, 123.0, 127.0, 162.6, 109.4, 153.3, 114.1, 30.1, 92.5), an isopropyl group (*δ*_C_ 72.2, 25.8, 24.3), and the substitution for the isopentenyl group (*δ*_C_ 115.2, 145.0. 71.9, 30.1, 30.0). Based on the above analysis, the plane structure of **8** was an analog of 7. The ^1^H-^1^H COSY correlation of H-1″(*δ*_H_ 7.0)/H-2″(*δ*_H_ 7.08), combined with HMBC correlations from H-2″ (*δ*_H_ 7.08) to C-8 (*δ*_C_ 109.4) and C-2″(*δ*_C_ 145.0) and from H-4″ (*δ*_H_ 1.44) and H-5″ (*δ*_H_ 1.44) to C-2″ (*δ*_C_ 145.0) and C-3″ (*δ*_C_ 71.9) indicated that the side group of C-8 was a 3″-methyl-3″-hydroxyl-1″-butenyl moiety. Subsequently, the absolute configuration of 2′R for **8** was determined by comparing the experimental and calculated ECD data (Figure 6). Therefore, the structure of compound **8** was identified as 2′,3′-dihydro-2′-(4′-hydroxymethyl-ethyl)-8-(3″-methyl-3″-hydroxyl-1″-butenyl)-2H-1-benzopyran-2-one (Figure 1).

In addition, twenty-two known compounds were identified as 6-(2′-hydroxyl-3′-methyl-3′-butenyl)-7-methoxyl-8-(3″-methyl-2″-butenyl)-2H-1-benzopyran-2-one (**9**) [9], 6-(3′-methyl-2′,3′-oxiranylbutyl)-7-methoxyl-8-(3″-methyl-2″-butenyl)-2H-1-benzopyran-2-one (**10**) [9], 6-(3′-methyl-2′,3′-dihydroxylbutyl)-7-methoxyl-8-(3″-methyl-2″-butenyl)-2H-1-benzopyran-2-one (**11**) [9], 6-(2′,3′-dihydroxy-3-methylbutyl)-8-prenylumbelliferone (**12**) [9], osthenol (**13**) [24], ulopterol (**14**) [25], (+)-trachypleuranin A (**15**) [26], scopoletin (**16**) [27], 7-(6R-hydroxy-3,7-dimethyl-2E,7-octadienyloxy) (**17**) [28], 6-methoxy-7-isoprenyloxycoumarin (**18**) [29], auraptene (**19**) [30], suberosin (**20**) [31], umbelliferone (**21**) [25], scopolin (**22**) [25], 7-O-(6-O-syringoyl-β-Dglucopyranosyl)coumarin (**23**) [31], 8,9-Dihydro-8-(1-hydroxy-1-methylethyl)-6-(3-methylbut-2-enyloxy)furo-2,3-h]-benzopyran-2-one (**24**) [32], (8S)-8,9-dihydro-8-(1-hydroxy-1-methylethyl)-6-hydroxy-2H-furo [2,3-H]-1-benzopyran-2-one (**25**) [33], demethylsuberosin (**26**) [34], imperatorin (**27**) [35], bergapten (**28**) [36], xanthotoxin (**29**) [37], and isopimpinellin (**30**) [38], respectively. Their structures are listed in Figure 1.

### 2.2. Network Pharmacology

#### 2.2.1. Construction and Analysis of the PPI Network

**Figure 7:**
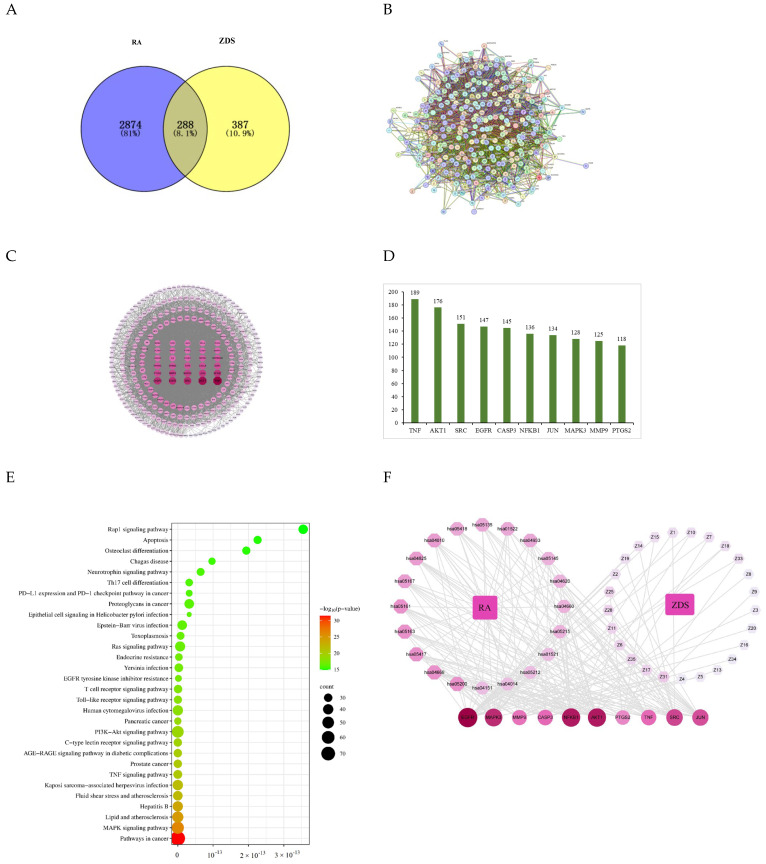
(**A**) Venn diagram showing the common target genes between ZDS and RA. (**B**,**C**) Overall PPI network and (**D**) the top 10 targets in order of degree value. (**E**) The top 30 KEGG pathways of hub genes and (**F**) the compound target pathway network.

The isolated compounds from ZDS were predicted component-related targets. After deleting the duplicate targets, the targets that could directly interact with each compound in the ZDS were retained. As a result, 675 targets were implicated in ZDS (Table S3). A total of 3133 known therapeutic targets for RA were obtained from the GeneCards database, combined with 42 known therapeutic targets for the treatment of RA collected from the OMIM database. After eliminating the redundancy, a total of 3162 known therapeutic targets in the treatment of RA were collected in this study (Table S4). Further, by taking an intersection of the compound target genes and disease-related genes, we finally obtained the ZDS target and RA-related gene set (Figure 7A). To further identify the core regulatory targets, PPI analysis was performed by submitting overlapping targets of active compounds in ZDS and RA to the STRING database. The network consisted of 287 nodes representing targets and 5169 edges denoting interactions, with a median connectivity degree of 36, illustrating a complex web of potential inter-target communications (Figure 7B). Nodes with higher degrees are presented in darker colors and larger sizes, indicating their closer correlation with RA. The top 10 genes in terms of the value of degrees are shown (Figure 7C). These key genes are TNF, AKT1, SRC, EGFR, CASP3, NFKB1, JUN, MAPK3, MMP9, and PTGS2 (Figure 7D), which may play a crucial role in the anti-RA effect of ZDS.

#### 2.2.2. KEGG Enrichment Analysis

In order to elucidate the functions and the enriched pathways of the potential anti-RA genes of ZDS, KEGG pathway enrichment analysis was carried out. The results of KEGG enrichment analysis showed that 317 signaling pathways for 288 target genes were obtained (*p* < 0.05, Table S5), and the top 30 enriched pathways are shown in Figure 7E. The results showed that the targets of ZDS in the treatment of RA were mainly enriched in pathways in cancer (n = 78), the MAPK signaling pathway (n = 55), the PI3K-Akt signaling pathway (n = 51), lipid and atherosclerosis (n = 46), Kaposi sarcoma-associated herpesvirus infection (n = 41), and so on, which could be the key pathways in the effect of ZDS against RA. Among them, pathways in cancer associated with the highest number of genes might be the most important ZDS-RA pathway.

#### 2.2.3. Compound-Target-Pathway Network Analyses

To visualize the relationship between ZDS compounds, candidate potential targets, and disease pathways, a ZDS–compound–target–RA interaction network (Figure 7F) was constructed to better predict the potential regulated actions of ZDS holistically. Each active compound is interconnected to multiple targets corresponding to signaling pathways. It may impose a synergistic effect on disease treatment.

### 2.3. Experimental Validation of the Anti-RA Action of ZDS In Vitro

#### 2.3.1. Cytotoxic Effects of New Coumarins in Primary HFLS-RA Cells

**Figure 8:**
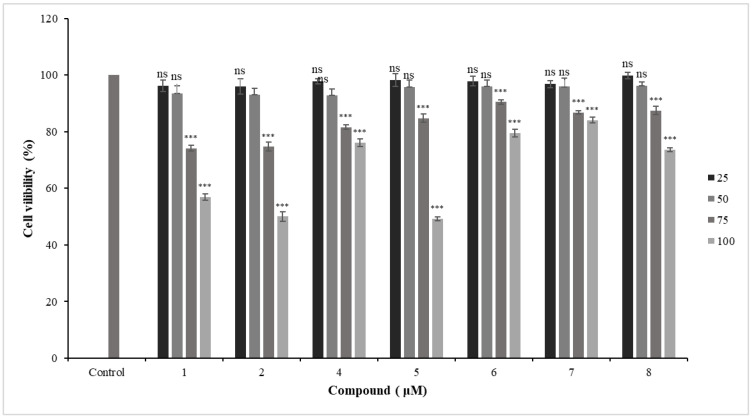
Cell viability of compounds **1**, **2**, and **4**–**8** of HFLS-RA. Data are expressed as mean ± SD (n = 3), vs. the control group, ns means non-significant and *** means *p* < 0.001.

To determine the effects of new coumarins on HFLS-RA survival, primary HFLS-RA cells were treated with different concentrations of coumarins (100, 75, 50, 25 μmol/L) for 24 h, and the cell viabilities were evaluated by the MTT assay. The results are shown in Figure 8. Compounds **1**–**2** and **4**–**8** with a dosage less than 50 μmol/L had no effect on cell viability of HFLS-RA, while compounds **1**–**2** and **4**–**8** exhibit significant cytotoxicity at concentrations of 75 and 100 μmol/L.

#### 2.3.2. Antiproliferative of HFLS-RA Cells

**Figure 9:**
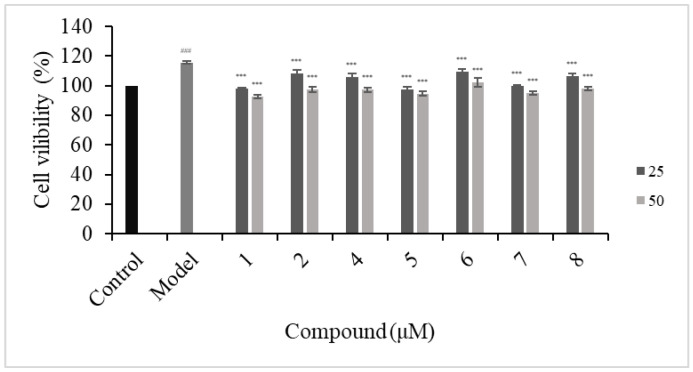
Effect of compounds **1**, **2**, and **4**–**8** on the proliferation viability of LPS (1 μg/mL)-induced HFLS-RA cells. Data are expressed as mean ± SD (n = 3), vs. the untreated group (Normol), ###, *p* < 0.001 vs. LPS-induced group, *** *p* < 0.001.

Previous studies have reported that the LPS-induced HFLS-RA model was used to study the drugs for the treatment of RA in vitro [39]. To investigate the possible inhibitory effects of compounds on LPS-induced HFLS-RA proliferation, HFLS-RA was incubated with LPS (1 μg/mL) in the presence or absence of compounds for 24 h. As shown in Figure 9, LPS (1 μg/mL) significantly improved the cell viability of HFLS-RA compared with the untreated control group. For compounds **1**, **2**, and **4**–**8** (50, 25 μmol/L), treatment significantly reversed the observed viability increase induced by LPS. Among them, compounds **1**, **5**, and **7** have the most significant effect. Therefore, 50, 25, and 12.5 μmol/L of compounds **1**, **5**, and **7** were selected as the final dose concentrations to further evaluate the effects on cytokines in LPS-induced HFLS-RA.

#### 2.3.3. Effects of Compounds **1**, **5**, and **7** on IL-1β, IL-6, and TNF-α Secretions

**Figure 10:**
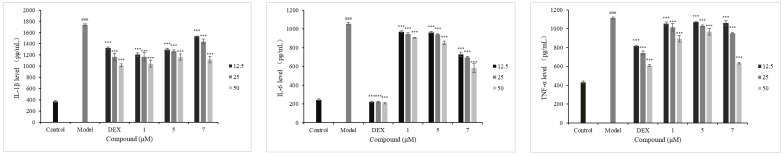
Effects of compounds **1**, **5**, and **7** on the levels of pro-inflammatory cytokines IL-1β, IL-6, and TNF-α in the LPS (1 μg/mL)-induced HFLS-RA cells. Data are expressed as mean ± SD (n = 3), vs. untreated group, ###, *p* < 0.001, vs. LPS-induced group, *** *p* < 0.001.

The effect of compounds **1**, **5**, and **7** on LPS-induced inflammation in HFLS-RA was investigated by ELISA. As shown in Figure 10, LPS stimulation significantly enhanced the protein levels of IL-6, IL-1β, and TNF-α compared with untreated HFLS-RA. Compounds **1**, **5**, and **7** obviously downregulated LPS-induced increases of IL-6, IL-1β, and TNF-α expression in HFLS-RA. Among them, compound **7** has a significantly higher inhibitory effect on IL-6 than compounds **1** and **5** at the test concentration.

### 2.4. Molecular Docking Analysis

**Figure 11:**
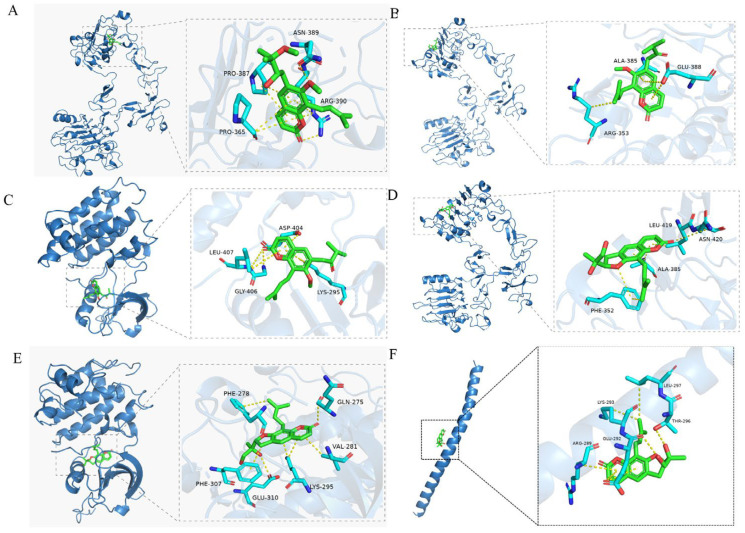
Docking results of the three active compounds of ZDS and the key RA-associated targets. (**A**) compound **1** and EGFR; (**B**) compound **5** and EGFR; (**C**) compound **5** and SRC; (**D**) compound **7** and EGFR; (**E**) compound **7** and SRC; (**F**) compound **7** and JUN.

To ascertain the related targets of compounds **1**, **5**, and **7**, we carried out a molecular docking analysis. The 3D structures of related genes including EGFR (1YY9), SRC (3GEQ), and JUN (1A02) were obtained from the PDB database. The binding free energies are shown in Table 4. Among the docking results, all compounds had a strong binding activity. The main binding complexes are displayed in Figure 11.

## 3. Discussion

The genus *Zanthoxylum* has important industrial applications. Most of the *Zanthoxylum* species are known not only for spices but also in traditional remedies [40]. With the deepening of research, the medicinal value of *Zanthoxylum* species has attracted increasing attention from researchers. Some of the diseases treated by *Zanthoxylum* species include malaria, rheumatism, cancer, stomachache, gastrointestinal disorders, gonorrhea, lung diseases, skin infections, febrifuge, anti-hemorrhagic, and genitourinary diseases [41]. One particular noteworthy underutilized herb medicine is *Z. dimorphophyllum* var. *spinifolium*, commonly known as “San Xue Fei”, which has a wide range of applications in southwest China. There has been very little research on its pharmacological activity until now, with only one report on its coumarin feeding deterrent activities against Tribolium castaneum [9].

Coumarins from the genus *Zanthoxylum* primarily belong to simple coumarins and furanocoumarins [15]. Among the new compounds in our study, compounds **1**–**6** were simple 7-methoxycoumarin derivatives, while compounds **7** and **8** belong to furanocoumarin derivatives. It is worth noting that the C-8 position of compounds **2**–**4**, **6**, and **8** is replaced by a 3″-methyl-1″-butenyl group, and compounds **4** and **6** have a hydroperoxyl group, respectively, which is a rare phenomenon in the genus *Zanthoxylum*. Compounds **13**–**16**, **16**, and **23** were first isolated from ZDS, while compounds **20** and **24**–**26** were first isolated from the genus *Zanthoxylum*.

The results of the KEGG pathway enrichment analysis of 288 genes suggested that 317 signaling pathways were directly linked to the occurrence and development of RA, indicating that these signaling pathways might be the mechanisms of ZDS coumarins against RA. Based on the degree value of each gene in the compound target pathway network, EGFR was the hub gene related to the compounds and targets (Figure 7F). From the results of cell experiments, most of the new compounds showed significant proliferation inhibitory activity against LPS-induced HFLS-RA cells. Meanwhile, many known compounds have also been reported to have anti-inflammatory activity [41,42,43,44,45]. Thus, it can be inferred that coumarin compounds should be one of the main components of anti-RA in ZDS. In addition, based on the results predicted by network pharmacology, cell experiments, and molecular docking, the effect of compound **7** on RA may be a synergistic effect of three targets, and it can be speculated that compound **7** is the most promising component for developing an anti-RA agent among the isolated new compounds. Therefore, the treatment of RA with ZDS has the characteristics of a multi-component, multi-target, and multi-pathway overall regulatory effect, and coumarin can be one of the main active ingredients in treating RA with ZDS.

## 4. Materials and Methods

### 4.1. General Experimental Procedures

A Bruker Avance NEO 600 (Bruker Corporation, Bremen, Germany) was used to obtain the 1D NMR and 2D NMR spectra and TMS as an internal standard. UV spectra were conducted by a Shimadzu UV-2401PC spectrometer (Shimadzu Corporation, Tokyo, Japan). A Bruker FT-IR (Bruker Corporation, Bremen, Germany) was used to record the IR spectra. ESI-MS and HRESIMS were recorded by Thermo ultimate 3000/Q EXACTIVE FOCUS (Thermo Fisher Scientific, Waltham MA, USA) and Agilent 1100 instrument mass spectrometers (Agilent Technologies Inc., Santa Clara, CA, USA), respectively. Optical rotations were acquired on a JASCOP-1020 polarimeter (Jasco Inc., Hiroshima, Japan). The compounds were poured from RP-18 gel (40–63 μm, Merck, Darmstadt, Germany), and column chromatography was performed on silica gel (300–400 mesh; Qingdao Marine Chemical Co., Ltd., Qingdao, China), Sephadex LH-20 (40–70 μm, Amersham Pharmacia Biotech AB, Uppsala, Sweden), and semi-preparative HPLC (Hitachi, Ltd., Tokyo, Japan) with an Agilent ZORBAX RP-18 column (250 mm× 9.4 mm, 5 μm, Agilent Technologies Inc., Santa Clara, CA, USA). All solvents used for general chromatography were analytical grade and purchased from Sinopharm Chemical Reagents Co. Ltd. (Shanghai, China). The solvents used for NMR were D-substituted solvents from Cambridge Isotope Laboratories (Cambridge, MA, USA), and the chromatographic grade solvents used for HPLC were purchased from Merck, Darmstadt, Germany. The TLC (GF 254) was obtained from Qingdao Marine Chemical Co., Ltd. (Qingdao, China). MTT (Biosharp, Hefei, China), fetal bovine serum (Cell-Box, Gippsland, Australia), Dulbecco′s modified eagle medium (DMEM), and phosphate-buffered saline (PBS) were purchased from Solarbio (Beijing, China).

### 4.2. Plant Material

The *Z. dimorphophyllum* var. *spinifolium*’s aerial part was collected from the Liupanshui Region, Guizhou Province of China, in September 2021 and identified by Prof. Wei Gu. A voucher specimen (NO. GZCNG 20210622) was deposited in the Natural Products Research Center of Guizhou Province.

### 4.3. Extract and Isolation

The air-dried and pulverized aerial part of *Z. dimorphophyllum* var. *spinifolium* (30.0 kg) was extracted with 120 L 80% EtOH under reflux three times (4 h each time) and obtained a crude residue (5.8 kg), which was chromatographed on a silica gel column (200–300 mesh) and successively eluted with petroleum ether–EtOAc (100:1 → 1:100, *v*/*v*), CHCl_3_/MeOH (20:1–1:1, *v*/*v*), and MeOH to yield nine fractions (Fr.A–Fr.I).

Fr C (75.2 g) was divided into 4 subfractions (Fr.C1–Fr.C4) over an MCI column eluted with MeOH-H_2_O (60:40 → 100:0, *v*/*v*). Fr. C1 (1.2 g) was fractionated by a Sephadex LH-20 column (MeOH) and further applied to semi-preparative RP–HPLC (Agilent ZORBAX SB–C18, MeOH: H_2_O, 50:50, *v*/*v*, 2.0 mL/min) to obtain **29** (36.8 mg, t*_R_* = 24.6 min). Compound **30** (71.2 mg) was obtained by recrystallization from Fr. C2. The filter liquor (7.3 g) of Fr. C2 was repeatedly chromatographed over a silica gel column to obtain **28** (36.8 mg). Fr. C3 (11.8 g) was chromatographed over a silica gel column (300–400 mesh) eluted with petroleum ether-EtOAc (100:1 → 1:100, *v*/*v*) to obtain Fr. C3–1 (209.3 mg) and was further applied to a silica gel column (petroleum ether-EtOAc, 90:10 → 70:30, *v*/*v*) to give **27** (128.2 mg), **19** (123.3 mg), and **18** (62.3 mg).

Fr.D (20.5 g) was separated into 10 subfractions (Fr.D1–Fr.D10) after an MCI column eluted with MeOH–H_2_O (50: 50 → 100: 0, *v*/*v*). Fr.D4 (8.2 g) was chromatographed over a silica gel column (300–400 mesh) eluted with petroleum ether-EtOAc (80:20 → 20:80, *v*/*v*) to give **26** (1.3 g) and 5 subfractions (Fr.D4–1–Fr.D4–5). Compound **1** (103.2 mg) was obtained by recrystallizing Fr.D4–2–1, and the mother liquor after recrystallization continues to pass through the semi-preparative RP-HPLC (Agilent ZORBAX SB-C18, MeOH: H_2_O, 74:26, *v*/*v*, 2.0 mL/min) to obtain **5** (50.3 mg, t*_R_* = 20.1) and **10** (60.3 mg, t*_R_* = 22.2). Fr. D4–2–2 (107.5 mg) was further subjected to a silica gel column (petroleum ether: EtOAc, 85:15 → 80:20, *v*/*v*) to give compound **20** (11.3 mg) and compound **14** (60.7 mg). Fr.D4–3 (1.0 g) was fractionated by Sephadex LH–20 column (MeOH) and further applied to silica gel column separation to obtain **9** (25.3 mg). Fr.D4–4 (1.4 g) was applied to silica gel using the gradient elution of petroleum ether-EtOAc, 70:30 → 60:40, *v*/*v*) and then subjected to Sephadex LH–20 to yield **13** (17.8 mg), **17** (3.5 mg), and **12** (10.4 mg).

Fr.F (132.8 g) was afforded 5 fractions (Fr.F–1–Fr.F-5) by an RP-18 column eluted with MeOH-H_2_O (30:70 → 100:0, *v*/*v*), and subsequent chromatography of Fr.F–2 (9.2 g) was performed on a silica gel column (petroleum ether: acetone, 80:20, *v*/*v*) and was further applied to a Sephadex LH–20 column (MeOH), which led the isolation of compounds **11** (870.2 mg), **15** (51.2 mg), **6** (6.3 mg), and **24** (51.2 mg).

Fr.G (82.6 g) was separated by an RP-18 column eluted with MeOH-H_2_O (30: 50 → 100:0, *v*/*v*) to obtain 7 subfractions (Fr.G1–Fr.G7). Fr.G–2 (10.5 g) was separated by silica gel CC (300–400 mesh, 65.6 g), eluted with CHCl_3_:MeOH (95:55 → 85:15, *v*/*v*), and further purified by a Sephadex LH-20 column to yield compounds **16** (30.5 mg), **21** (20.6 mg), and **25** (33.9 mg). Fr.G–3 (11.0 g) was separated by silica gel (300–400 mesh) eluted with CHCl_3_:MeOH (95:55 → 85:15, *v*/*v*) to afford compounds **2** (5.3 mg), **3** (17.6 mg), **4** (41.3 mg), **7** (32.1 mg), and **8** (5.6 mg).

Fr.H (600.2 g) was separated into 7 subfractions (Fr.H-1–Fr.H-7) after an RP–18 column eluted with MeOH-H_2_O (10:90 → 0:100, *v*/*v*). Compounds **22** (40.8 mg) and **23** (10.3 mg) were isolated from Fr.H–2 (32.1g) after repeated silica gel column chromatography.

Spinifoliumasone A (**1**): yellow, amorphous solid; [*α*]25D + 71.4 (c = 1.0, MeOH), UV (MeOH) *λ*max (log *ε*): 208 (6.74), 294 (6.15) nm; IR (KBr) νmax 3481, 2977, 1731, 1606 cm^−1^; HRESIMS at *m*/*z* 383.1840 [M + Na]^+^ [calcd. for C_21_H_28_O_5_Na *m*/*z* 383.1830].

Spinifoliumasone B (**2**): yellow, amorphous solid; [*α*]25D +81.8 (c = 1.0, MeOH); UV (MeOH) *λ*max (log *ε*): 218 (6.40), 300 (5.92) nm; IR (KBr) νmax 3420, 2976, 1704, 1631 cm^−1^; HRESIMS at *m*/*z* 399.1789 [M + Na]^+^ [calcd. for C_21_H_28_O_6_Na *m*/*z* 399.1778].

Spinifoliumasone C (**3**): white, amorphous solid; [α]25D + 266.7 (c = 1.0, MeOH); *λ*max (log *ε*): 215 (4.24), 300 (3.75) nm; IR (KBr) νmax 2972, 1714, 1601 cm^−1^; HRESIMS at *m*/*z* 385.1616 [M + H]^+^ [calcd. for C_21_H_25_O_6_ *m*/*z* 385.1622].

Spinifoliumasone D (**4**): white, amorphous solid; [α]25D +267.5 (c = 1.0, MeOH); *λ*max (log *ε*): 218 (4.44), 300 (4.12) nm; IR (KBr) νmax 2976, 1719, 1602 cm^−1^; HRESIMS at *m*/*z* 415.1719 [M + Na]^+^ [calcd. for C_21_H_25_O_6_Na *m*/*z* 415.1727].

Spinifoliumasone E (**5**): white, amorphous solid; *λ*max (log *ε*): 208 (6.74), 294 (6.15) nm; −1; HRESIMS at *m*/*z* 329.1751 [M + H]^+^ [calcd. for C_21_H_25_O_6_ *m*/*z* 329.1747].

Spinifoliumasone F (**6**): white, amorphous solid; [α]25D + 58.8 (c = 1.0, MeOH); *λ*max (log *ε*): 218 (4.42), 300 (3.99) nm; IR (KBr) νmax 2975, 1712, 1650 cm^−1^; HRESIMS at *m*/*z* 383.1459 [M + Na]^+^ [calcd. for C_21_H_25_O_6_Na *m*/*z* 383.1465].

Spinifoliumasone G (**7**): yellow, amorphous solid; [α]25D − 312.5 (c = 1.4, MeOH); UV (MeOH) *λ*max (log *ε*): 202 (4.09), 340 (3.56) nm; ECD (MeOH) *λ* (Δ *ε*) 233 (19.10), 284 (3.37), 349 (−1.21) nm; IR (KBr) νmax 3418, 2915, 1705, 1618 cm^−1^; HRESIMS at *m*/*z* 353.1369 [M + Na]^+^ [calcd. for C_19_H_22_O_5_Na *m*/*z* 353.1359].

Spinifoliumasone H (**8**): yellow, amorphous solid; [α]25D − 189.4 (c = 1.0, MeOH); UV (MeOH) *λ*max (log *ε*): 220 (3.97), 338 (3.67) nm; ECD (MeOH) *λ* (Δ *ε*) 215 (5.08), 226 (6.93), 255 (−4.60), 301 (−0.82), 341 (−3.92) nm; IR (KBr) νmax 3420, 2976, 1704, 1631 cm^−1^; HRESIMS at *m*/*z* 353.1353 [M + Na]^+^ [calcd. for C_19_H_22_O_5_Na *m*/*z* 353.1359].

### 4.4. Electronic Circular Dichroism Calculation of Compounds 7 and 8

Stable conformers were used in the CONFLEX searches based on molecular mechanics with MMFF94S force fields [46]. Distributions higher than 1% were selected for further optimization by the DFT (density functional theory) method at the B3LYP/6-311+g (d) level in the Gaussian 09 program package. The selected ECD conformers were calculated by the TD-DFT method at the CAM-B3LYP/tzvp levels with the CPCM model in a methanol solution. SpecDis 1.51 was used to generate the calculated ECD curve [47].

### 4.5. Determination of the Absolute Configuration of 1 and 7

#### 4.5.1. Preparation of the Rh_2_(OCOCF_3_)_4_ Complex of Compound **1**

Rh_2_(OCOCF_3_)_4_ (1.06 mg) was dissolved in anhydrous dichloromethane (500 μL) at room temperature. Then, this solvent was added to vacuum-dried compound **1** (0.5 mg), and the experimental CD spectrum was recorded immediately. The complex-induced CD spectra were recorded every 10 min for 4 times. The intrinsic CD of **1** was subtracted. The absolute configuration of the compound was assigned by the positive and negative Cotton effects at around 350 nm, according to the bulkiness rule [23].

#### 4.5.2. Preparation of the Mo_2_(OAc)_4_ Complex of Compound **7**

Compound **7** (0.4 mg) with 1.0 mg Mo_2_(OAc)_4_ was mixed in 500 μL dry DMSO. The first ECD values were recorded immediately. The complex-induced CD spectra were recorded every 10 min for 4 times. The inherent ECD spectrum was subtracted. The absolute configuration of **7** was determined by the sign at around 310 nm in the induced ECD spectra [22].

### 4.6. Network Pharmacology Analysis

#### 4.6.1. Target Prediction of ZDS

The tool SwissTargetPrediction (available at http://www.swisstargetprediction.ch, accessed on 7 December 2023) was utilized to gather all targets of individual compounds using the simplified molecular input line entry system (SMILES) limited to “Homo sapiens”. For further analysis, only those targets with a combined score of 0 or higher were chosen [48].

#### 4.6.2. Acquisition of RA-Associated Genes

The term “rheumatoid arthritis” was used as the query, and relevant rheumatoid arthritis targets were searched for in the OMIM database (https://omim.org/, accessed on 23 December 2023) and the GeneCards database (https://www.genecards.org/, accessed on 23 December 2023) [47]. Targets acquired from the GeneCards database were organized based on their relevance score values, with a threshold of score ≥1 used to filter disease targets. A higher relevance score indicates a stronger association between the target and the disease. Targets obtained from both databases were compiled, and duplicates were removed to generate the list of rheumatoid arthritis (RA) targets for further investigation. A Venn diagram (available at https://bioinfogp.cnb.csic.es/tools/venny/, accessed on 23 December 2023) was used to map the target genes for ZDS in RA and to identify potential targets [48].

#### 4.6.3. Construction of a Protein–Protein Interaction Network

A protein–protein interaction (PPI) network for the intersecting targets was constructed using the STRING online database (https://cn.string-db.org/, accessed on 23 December 2023). The species was set to “Homo sapiens”, and only protein interactions with a confidence score greater than 0.400 were included. Free targets were excluded from the network, and the remaining results were saved in TSV format. These TSV files were then imported into Cytoscape 3.9.0 software to visualize the PPI network. Network topology analysis was performed using the network analyzer feature within Cytoscape 3.9.0 software [49]. The top 10 targets with the highest degree were selected as the core targets, and the corresponding drug ingredients were considered the main active ingredients for RA.

#### 4.6.4. KEGG Analysis

The Kyoto Encyclopedia of Genes and Genomes (KEGG) pathway analysis utilized a functional annotation database, DAVID (https://david.ncifcrf.gov/, accessed on 24 December 2023), and selected the top 30 enriched pathways to create bubble charts for visual presentation [49].

#### 4.6.5. Network Construction and Analysis

A network construction of “components-targets-pathways-diseases” was performed by the network visualization software Cytoscape (ver. 3.5.0).

### 4.7. Anti-RA Activity Screening

#### 4.7.1. Cell Culture

HFLS-RA cells were cultured in DMEM (11995; Solarbio, Beijing, China) containing 10% (*v*/*v*) heat-inactivated fetal bovine serum (AUS-01S-02; Cell-Box, Australia), 100 U/mL penicillin, and 100 μg/mL streptomycin under conditions of 37 °C and 5% CO_2_ [49]. The cells in the exponential growth period were used in all the experiments.

#### 4.7.2. Cell Cytotoxic Assay

HFLS-RA cells (1 × 10^4^ cells/well) were seeded in a 96-well plate with DMEM supplemented with 10% (*v*/*v*) FBS (AUS-01S-02; Cell-Box, Gippsland, Australia), penicillin (100 U/mL), and streptomycin (100 mg/mL) (Biosharp, Hefei, China). After 24 h of incubation, the cells were treated with different compound concentrations (100, 75, 50, and 25 μM) for 24 h. Following drug treatment, 10 µL MTT (BS350B; Biosharp, Hefei, China) was added to each well, and the cells were incubated at 37 °C for 4 h [50]. The absorbance at 490 nm was read using a spectrophotometer (Bio Tek, Winooski, VT, USA). The OD values were used to calculate cell viability.

#### 4.7.3. Cell Proliferation Assay

HFLS-RA cells (1 × 10^4^ cells/well) were seeded in a 96-well plate with DMEM supplemented with 10% (*v*/*v*) FBS (AUS-01S-02; Cell-Box, Australia), penicillin (100 U/mL), and streptomycin (100 mg/mL) (Biosharp, Hefei, China). After 24 h of incubation, the cells were exposed to 1 μg/mL LPS and co-treated with different concentrations of compounds for 24 h. A total of 10 µL MTT (BS350B; Biosharp, Hefei, China) was added to each well after drug treatment, and the cells were incubated at 37 °C for 4 h [49]. The absorbance at 490 nm was read using a spectrophotometer (Bio Tek, USA). The OD values were used to calculate cell viability.

#### 4.7.4. Enzyme-Linked Immunosorbent Assay (ELISA)

HFLS-RA cells (1 × 10^4^ cells/well) were seeded in a 96-well plate with DMEM supplemented with 10% (*v*/*v*) FBS (AUS-01S-02; Cell-Box, Australia), penicillin (100 U/mL), and streptomycin (100 mg/mL) (Biosharp, Hefei, China). After 12 h of incubation, the cells were exposed to 1 μg/mL LPS and co-treated with different concentrations of compounds for 24 h. The culture supernatant was collected to evaluate the levels of pro-inflammatory cytokines IL-1β, IL-6, and TNF-α using ELISA kits (RX106152H, RX104793H; Ruixin Biotechnology Co., Ltd., Quanzhou, China), according to the manufacturer′s instructions.

#### 4.7.5. Statistical Analysis

Data are expressed as the mean standard deviation. SPSS 26.0 software (IBM SPSS, Armonk, NY, USA) was used for statistical analysis. Student′s t-test was performed for group comparisons. *p* < 0.05 indicated a statistically significant result [51].

### 4.8. Molecular Docking

Molecular docking was performed to assess the interaction between compounds **1**, **5**, and **7** and their related targets predicted by network pharmacology. Compound-related genes were downloaded from the Protein Data Bank (PDB) database (https://www.rcsb.org/, accessed on 8 September 2024). Autodock Vina (version 1.1.2) was used to dock compounds **1**, **5**, and **7** [52]. The best affinity was selected as the final docking conformation and visualized in Pymol 2.4.

## 5. Conclusions

Chemical investigation on *Z. dimorphophyllum* var. *spinifolium* led to the isolation of eight new coumarins together with twenty-two known ones. Their potential mechanisms against RA were predicted by network pharmacology. As a result, the MAPK signaling pathway and the PI3K-Akt signaling pathway were speculated to be the hub signaling pathway of ZDS against RA, and EGFR was the hub gene related to the compounds and targets. Further in vitro experimental verification of anti-RA was established, and it was found that compounds **1**, **2**, and **4**–**8** inhibited the proliferation of LPS-induced HFLS-RA cells. Compounds **1**, **5**, and **7** could significantly reduce the level of IL-1β, IL-6, and TNF-α in LPS-induced HFLS-RA cells, while compound 7 has a significantly higher inhibitory effect on IL-6 than compounds **1** and **5** at the test concentration, and this is consistent with the results of molecular docking verification. In conclusion, this study provides the first evidence of the anti-rheumatoid arthritis activity of **1**, **5**, and **7** in *Z. dimorphophyllum* var. *spinifolium*, which provides basic scientific evidence to support its traditional use. Compound **7** has the potential to be developed as an anti-RA drug.

## Figures and Tables

**Table 1:** ^1^H NMR (600 MHz) data of compounds **1**–**4** (*δ* in ppm, *J* in Hz).

Position	1 *^b^*	2 *^b^*	2 *^a^*	3 *^b^*	4 *^a^*
3	6.30 d (9.6)	6.34 d (9.6)	6.34 d (9.6)	6.32 d (9.6)	6.21 d (9.6)
4	7.86 d (9.6)	7.88 d (9.6)	7.63 d (9.6)	7.87 d (9.6)	7.59 d (9.6)
5	7.43 s	7.47 s	7.28 s	7.44 s	7.30 s
1′α	2.52 dd (14.4, 10.8)	2.54 dd (14.4, 10.8)	2.65 dd (14.4, 10.8)	2.52 dd (14.4, 10.8)	2.59 dd (14.4, 10.8)
1′β	3.09 dd (14.4, 2.4)	3.12 dd (14.4, 2.4)	2.95 dd (14.4, 1.8)	3.12 dd (14.4, 1.8)	2.96 dd (14.4, 2.4)
2′	3.70 dd (10.8, 2.4)	3.59 dd (10.8, 2.4)	3.63 brd (10.8)	3.58 dd (10.8, 1.8)	3.83 dd (10.8, 2.4)
3′					
4′	1.25 s	1.28 s	1.33 s	1.28 s	1.30 s
5′	1.23 s	1.27 s	1.30 s	1.27 s	1.29 s
1″α	3.50 dd (14.4, 6.6)	6.74 overlapped	6.71 d (16.8)	6.82 d (16.8)	6.61 d (16.6)
1″β	3.56 dd (14.4, 6.6)				
2″	5.22 t (6.6)	6.74 overlapped	6.77 d (16.8)	6.92 d (16.8)	6.86 d (16.6)
4″	1.84 s	1.43 s	1.43 s	1.43 s	1.50 s
5″	1.68 s	1.43 s	1.43 s	1.44 s	1.48 s
OCH_3_−7	3.82 s	3.80 s	3.78 s	3.80 s	3.76 s
OCH_3_−3′	3.30 s				3.32 s
OCH_3_−3″		3.30 s	3.28 s		
OH−2′			2.61 brs		2.56 brs
OH−3′	2.62 brs overlapped		2.14 brs		
OOH					9.80 brs

*^a^* 150 MHz in CDCl_3_. *^b^* 150 MHz in CD_3_OD, s, singlet; d, doublet; dd, double doublet; t, triplet; brs, broad singlet; brd, broad doublet.

**Table 2:** ^13^C NMR (150 MHz) data of compounds **1**-**8** (*δ* in ppm).

Position	1 *^b^*	2 *^b^*	3 *^b^*	4 *^a^*	5 *^a^*	6 *^a^*	7 *^b^*	8 *^b^*
2	163.2	162.8	163.0	161.5	160.9	161.5	164.0	163.6
3	114.9	115.1	114.9	114.0	115.0	115.2	111.6	111.9
4	146.0	146.1	146.2	144.4	143.4	144.4	146.8	146.8
5	129.5	130.5	130.1	129.0	127.8	129.1	122.4	123.0
6	132.2	132.3	132.2	130.2	125.1	129.3	132.3	127.0
7	161.4	161.3	161.3	159.7	159.6	159.7	163.2	162.6
8	124.2	119.6	119.9	118.2	123.6	118.4	112.2	109.4
9	153.0	152.4	152.4	150.8	152.6	150.9	154.2	153.3
10	116.8	116.8	116.7	115.2	115.5	115.2	114.2	114.1
1′	32.8	32.9	32.9	31.5	41.5	36.2	30.2	30.1
2′	78.6	79.4	79.4	76.2	211.7	74.9	88.8	92.5
3′	77.5	73.8	73.8	77.3	40.8	147.0	74.8	72.2
4′	20.5	25.9	25.9	20.3	18.4	111.0	67.6	25.8
5′	21.7	24.9	25.0	20.4	18.4	18.2	20.5	24.3
1″	23.8	119.3	116.0	118.2	23.1	117.9	23.2	115.2
2″	122.9	143.4	146.2	142.0	121.4	142.3	122.1	145.0
3″	133.5	77.2	71.9	82.6	132.9	82.6	133.8	71.9
4″	18.2	26.2	29.9	24.4	18.4	24.4	18.1	30.1
5″	25.8	26.1	29.9	24.5	25.7	24.5	25.9	30.0
7−OCH_3_	62.2	61.6	61.4	60.9	61.8	61.1		
3′−OCH_3_	49.7			49.4				
3″−OCH_3_		51.0						

*^a^* 150 MHz in CDCl_3_. *^b^* 150 MHz in CD_3_OD.

**Table 3:** ^1^H NMR (600 MHz) data of compounds **5**-**8** (*δ* in ppm, *J* in Hz).

Position	5 *^a^*	6 *^a^*	7 *^b^*	8 *^b^*
3	6.35 d (9.6)	6.21 d (9.6)	6.15 d (9.6)	6.24 d (9.6)
4	7.63 d (9.6)	7.59 d (9.6)	7.80 d (9.6)	7.85 d (9.6)
5	7.15 s	7.26 s	7.47 s	7.3 s
1′α	3.84 s	2.95 dd (13.8, 3.6)	3.35 dd (15.6, 9.0)	3.28 ddd (15.0, 9.0, 1.2)
1′β		2.79 dd (13.8, 9.6)	3.24 dd (15.6, 9.0)	3.23 ddd (15.0, 9.0, 1.2)
2′		4.43 dd (9.6, 3.6)	4.94 t (9.0)	4.83 dd (9.0, 7.8)
3′	2.78 m			
4′	1.19 d (7.2)	5.07 s	3.55 d (11.4)	1.36 s
		4.90 s	3.74 d (10.8)	
5′	1.19 d (7.2)	1.88 s	1.2 s	1.30 s
1″α	3.56 d (6.6)	6.62 d (16.8)	3.46 brd (7.2)	7.00 d (16.8)
1″β				
2″	5.24 t (6.6)	6.86 d (16.8)	5.28 t (7.2)	7.08 d (16.8)
4″	1.84 s	1.48 s	1.83 s	1.44 s
5″	1.69 s	1.51 s	1.67 s	1.44 s
OCH_3_−7	3.74 s	3.77 s		
OOH		9.65		
OH−3′				4.59 brs

*^a^* 150 MHz in CDCl_3_. *^b^* 150 MHz in CD_3_OD, s, singlet; d, doublet; dd, two doublets; t, triplet; brs, broad singlet; brd, broad doublet; ddd, three doublets; m, multiplet.

**Table 4:** The binding energy between targets and compounds.

Compound	Target	Binding Energy (Kcal/mol)
7	JUN	−5.3
7	SRC	−8.5
5	SRC	−8.5
1	EGFR	−6.1
5	EGFR	−6.0
7	EGFR	−6.7

## Data Availability

All data are contained in this article and the Supplementary Materials.

## Supplementary Materials

The following supporting information can be downloaded at https://www.mdpi.com/article/10.3390/molecules29184395/s1, Figures S1–S9: 1D/2D NMR, HR-ESIMS, IR, and UV spectra of compound **1**; Figures S10–S18: 1D/2D NMR, HR-ESIMS, IR, and UV spectra of compound **2**; Figures S19–S27: 1D/2D NMR, HR-ESIMS, IR, and UV spectra of compound **3**; Figures S28–S36: 1D/2D NMR, HR-ESIMS, IR, and UV spectra of compound **4**; Figures S37–S45: 1D/2D NMR, HR-ESIMS, IR, and UV spectra of compound **5**; Figures S46–S54: 1D/2D NMR, HR-ESIMS, IR, and UV spectra of compound **6**; Figures S55–S65: 1D/2D NMR, HR-ESIMS, IR, UV, and CD spectra of compound **7**; Figure S66: B3LYP/6-311+g (d) optimized the lowest energy 3D conformer of **7**; Figures S66–S76: 1D/2D NMR, HR-ESIMS, IR, UV, and CD spectra of compound **8**; Figure S77: B3LYP/6-311+g (d) optimized the lowest energy 3D conformer of **8**; Tables S1 and S2: Calculated ECD spectrum of **7**–**8**; Table S3: Targets of isolated compounds; Table S4: Targets of RA; Table S5: Putative targets of ZDS against RA; Table S6: Results of KEGG pathway enrichment analysis of ZDS putative targets. Table S7: Cell viability of HFLS-RA. Table S8: Cell proliferation viability of LPS-induced HFLS-RA. Tables S9–S11: Effects of compounds **1**, **5**, and **7** on the levels of pro-inflammatory cytokines IL-1β, IL-6, and TNF-α.
