# Supplementary material for: Discovery of Coumarins from Zanthoxylum dimorphophyllum var. spinifoliumas and Their Potential against Rheumatoid Arthritis
Source: Molecules. 2024 Sep 16;29(18):4395. doi: 10.3390/molecules29184395 (PMC11433664; doi:10.3390/molecules29184395)
Supplement: Supplementary file 1 [file molecules-29-04395-s001.zip › molecules-3145253-supplementary.pdf]

Discovery of eight undescribed coumarins from *Zanthoxylum dimorphophyllum* var. *spinifoliumas* and their potential against rheumatoid arthritis

Caixia Du[a, b, c, f], Junlei Chen[a, b], Lili Luo[a, b], Chunmao Yuan[a, b], Jue Yang[a, b], Xingyu Li [a, d, *], Xiaojiang Hao[a, b, e, *], Wei Gu[a, b, *]

a *State Key Laboratory of Functions and Applications of Medicinal Plants, Guizhou Medical University, Guiyang, 550014, PR China*

b *The Key Laboratory of Chemistry for Natural Products of Guizhou Province and Chinese Academy of Sciences, Guiyang, 550014, PR China*

c School of Basic Medicine, Guizhou Medical University, Guiyang, 550014, PR China

d College of Science, Yunnan Agricultural University, Kunming, 650201, PR China

e *State Key Laboratory of Phytochemistry and Plant Resources in West China, Kunming Institute of Botany, Chinese Academy of Sciences, Kunming, 650201, PR China*

f Bijie Medical College, Bijie, 551700, PR China

* *Correspondence: guwei2009@126.com (W G), haoxj@mail.kib.ac.cn (X. H), lixingyu@ynau.edu.cn (X. L)*

Supporting Information

Content

Discovery of eight undescribed coumarins from *Zanthoxylum dimorphophyllum* var. *spinifoliumas* and their potential against rheumatoid arthritis 1

Supporting Information 2

Figure S1. ^{1}H NMR (600 MHz, CD_3OD) spectrum of 1. 6

Figure S2. ^{13}C NMR (150 MHz, CD_3OD) spectrum of 1. 7

Figure S3. COSY spectrum of *1*. 7

Figure S4. HSQC spectrum of 1. 8

Figure S5. HMBC spectrum of 1. 8

Figure S6. ROESY spectrum of 1. 9

Figure S7. HR-ESI-MS spectrum of 1. 9

Figure S8. IR spectrum of 1. 10

Figure S9. UV spectrum of 1. 10

Figure S10. ^{1}H NMR (600 MHz, CD_3OD) spectrum of 2. 11

Figure S11. ^{13}C NMR (150 MHz, CD_3OD) spectrum of 2. 11

Figure S12. COSY spectrum of 2. 12

Figure S13. HSQC spectrum of 2. 12

Figure S14. HMBC spectrum of 2. 13

Figure S15. ROESY spectrum of 2. 13

Figure S16. HR-ESI-MS spectrum of 2. 14

Figure S17. IR spectrum of 2. 14

Figure S18. UV spectrum of 2. 15

Figure S19. ^{1}H NMR (600 MHz, CD_3OD) spectrum of 3 15

Figure S20. ^{13}C NMR (150 MHz, CD_3OD) spectrum of 3. 16

Figure S21. COSY spectrum of 3. 16

Figure S22. HSQC spectrum of 3. 17

Figure S23. HMBC spectrum of 3. 17

Figure S24. ROESY spectrum of 3. 18

Figure S25. HR-ESI-MS spectrum of 3. 18

Figure S26. IR spectrum of 3. 19

Figure S27. UV spectrum of 3. 19
Figure S28. ^{1}H NMR (600 MHz, $CDCl_3$) spectrum of 4. 20
Figure S29. ^{13}C NMR (150 MHz, $CDCl_3$) spectrum of 4. 20
Figure S30. COSY spectrum of 4. 21
Figure S31. HSQC spectrum of 4. 21
Figure S32. HMBC spectrum of 4. 22
Figure S33. ROESY spectrum of 4. 22
Figure S34. HR-ESI-MS spectrum of 4. 23
Figure S35. IR spectrum of 4. 23
Figure S36. UV spectrum of 4. 24
Figure S37. ^{1}H NMR (600 MHz, CD_3OD) spectrum of 5. 24
Figure S38. ^{13}C NMR (150 MHz, CD_3OD) spectrum of 5. 25
Figure S39. COSY spectrum of 5. 25
Figure S40. HSQC spectrum of 5. 26
Figure S41. HMBC spectrum of 5. 26
Figure S42. ROESY spectrum of 5. 27
Figure S43. HR-ESI-MS spectrum of 5. 27
Figure S44. IR spectrum of 5. 28
Figure S45. UV spectrum of 5. 28
Figure S46. ^{1}H NMR (600 MHz, CD_3OD) spectrum of 6. 29
Figure S47. ^{13}C NMR (150 MHz, CD_3OD) spectrum of 6. 29
Figure S48. COSY spectrum of 6. 30
Figure S49. HSQC spectrum of 6. 30
Figure S50. HMBC spectrum of 6. 31
Figure S51. ROESY spectrum of 6. 31
Figure S52. HR-ESI-MS spectrum of 6. 32
Figure S53. IR spectrum of 6. 32
Figure S54. UV spectrum of 6. 33
Figure S55. ^{1}H NMR (600 MHz, CD_3OD) spectrum of 7. 33
Figure S56. ^{13}C NMR (150 MHz, CD_3OD) spectrum of 7. 34
Figure S57. COSY spectrum of 7. 34
Figure S58. HSQC spectrum of 7. 35

Figure S59. HMBC spectrum of 7. 35
Figure S60. ROESY spectrum of 7. 36
Figure S61. HR-ESI-MS spectrum of 7. 36
Figure S63. IR spectrum of 7. 37
Figure S64. UV spectrum of 7. 37
Figure S65. CD spectrum of 7. 38
Figure S66. B3LYP/6-311+g (d) optimized lowest energy 3D conformers of 7. 38
Figure S67. ^{1}H NMR (600 MHz, CD_3OD) spectrum of 8. 39
Figure S68. ^{13}C NMR (150 MHz, CD_3OD) spectrum of 8. 39
Figure S69. COSY spectrum of 8. 40
Figure S70. HSQC spectrum of 8. 40
Figure S71. HMBC spectrum of 8. 41
Figure S72. ROESY spectrum of 8. 41
Figure S73. HR-ESI-MS spectrum of 8. 42
Figure S74. IR spectrum of 8. 42
Figure S75. UV spectrum of 8. 43
Figure S76. CD spectrum of 8. 43
Figure S77. B3LYP/6-311+g (d) optimized lowest energy 3D conformers of **8**. 44
Table S1. Calculated ECD spectrum of 7 45
Table S2. Calculated ECD spectrum of 8 46
Table S3. 675 tagets of isolated componds. 47
Table S4. 3162 targets of RA 51
Table S5. 288 putative targets of ZDS against RA. 69
Table S6. Results of KEGG pathway enrichment analysis of ZDS putative targets. 71
Table S7. Cell viability of HFLS-RA. 75
Table S8. Cell proliferation viability of LPS induced HFLS-RA. 75
Table S9. Effects of compounds 1, 5 and 7 on the levels of pro-inflammatory cytokines IL-1β. 75
Table S10. Effects of compounds 1, 5 and 7 on the levels of pro-inflammatory cytokines IL-6. 76
Table S11. Effects of compounds 1, 5 and 7 on the levels of pro-inflammatory cytokines TNF-α. 76

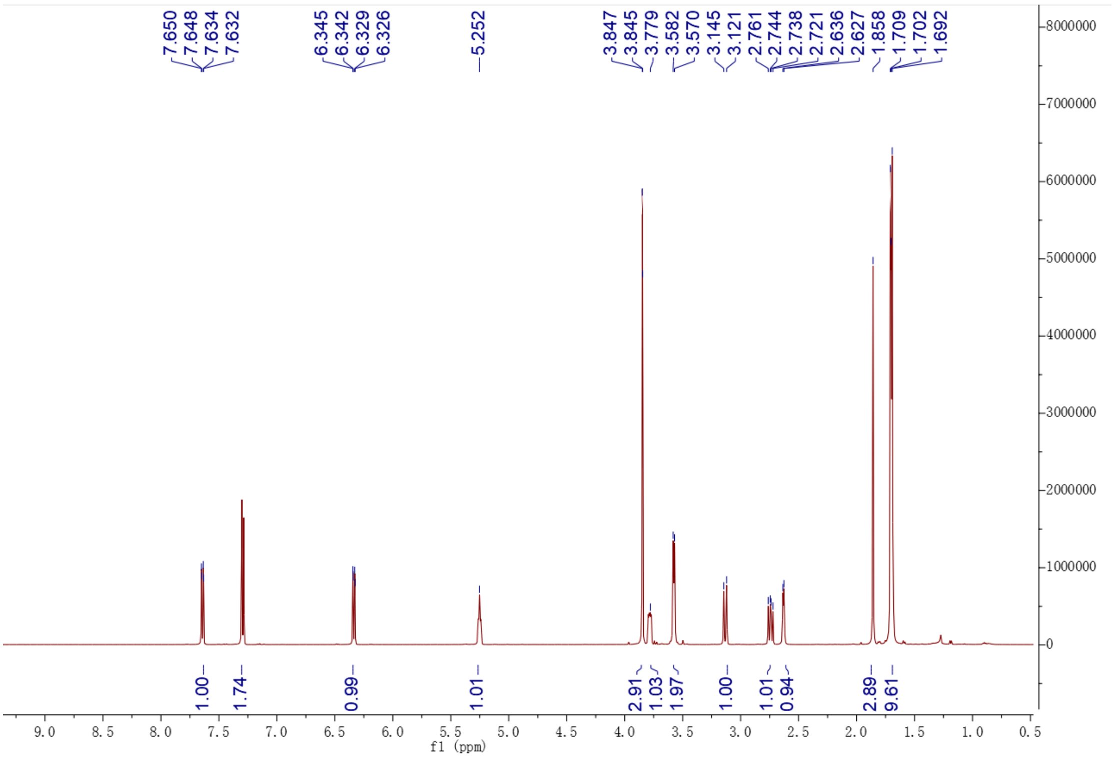

Figure S1. ^{1}H NMR (600 MHz, CD_3OD) spectrum of 1.

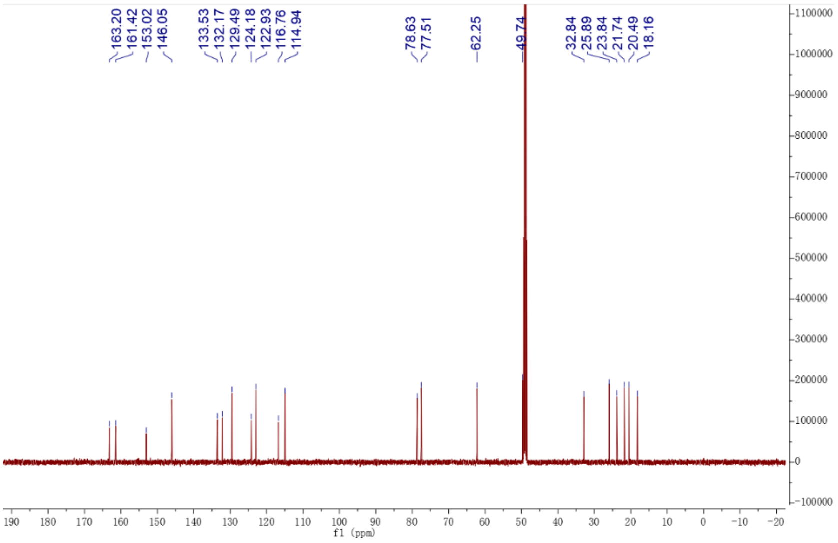

Figure S2. ^{13}C NMR (150 MHz, CD_3OD) spectrum of **1**.

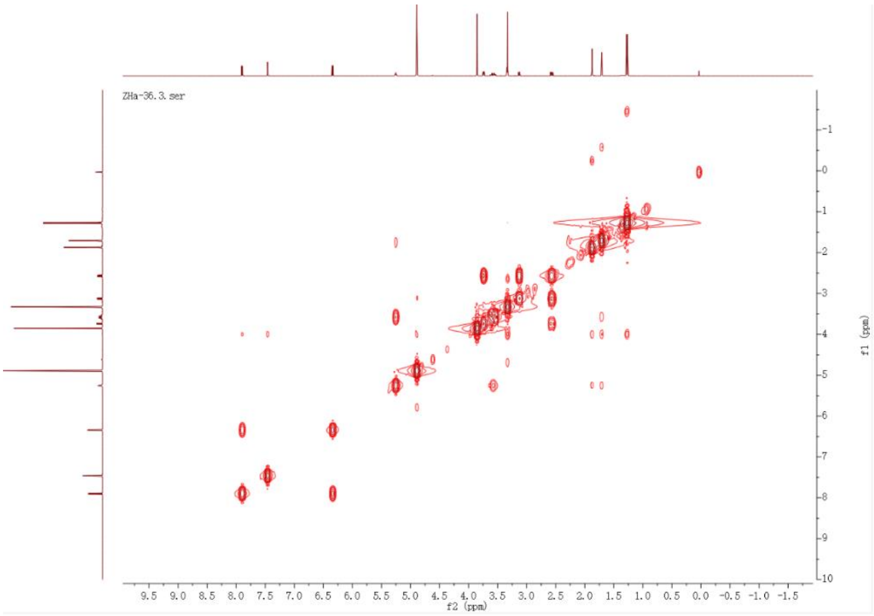

Figure S3. COSY spectrum of *1*.

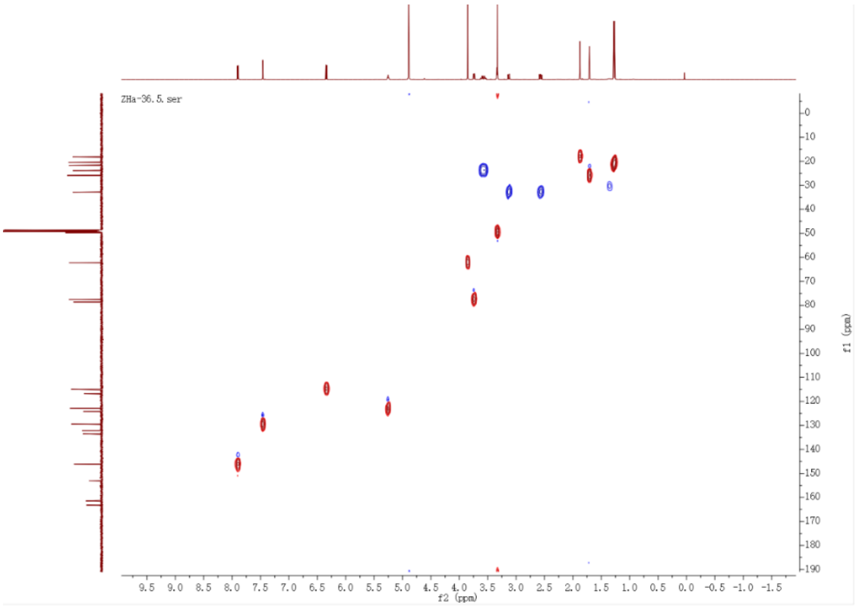

Figure S4. HSQC spectrum of **1**.

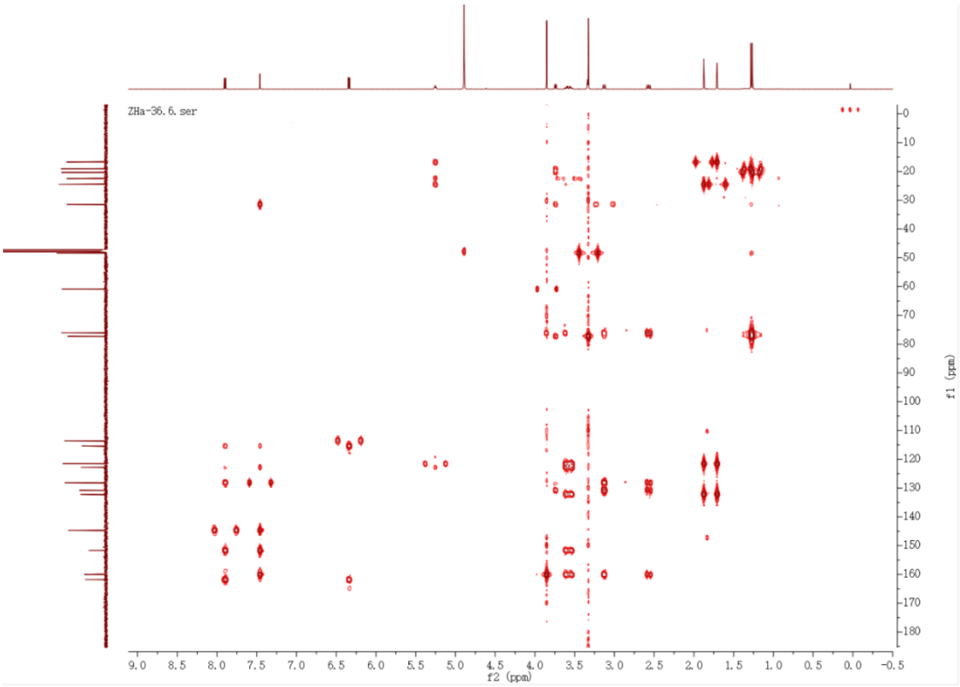

Figure S5. HMBC spectrum of **1**.

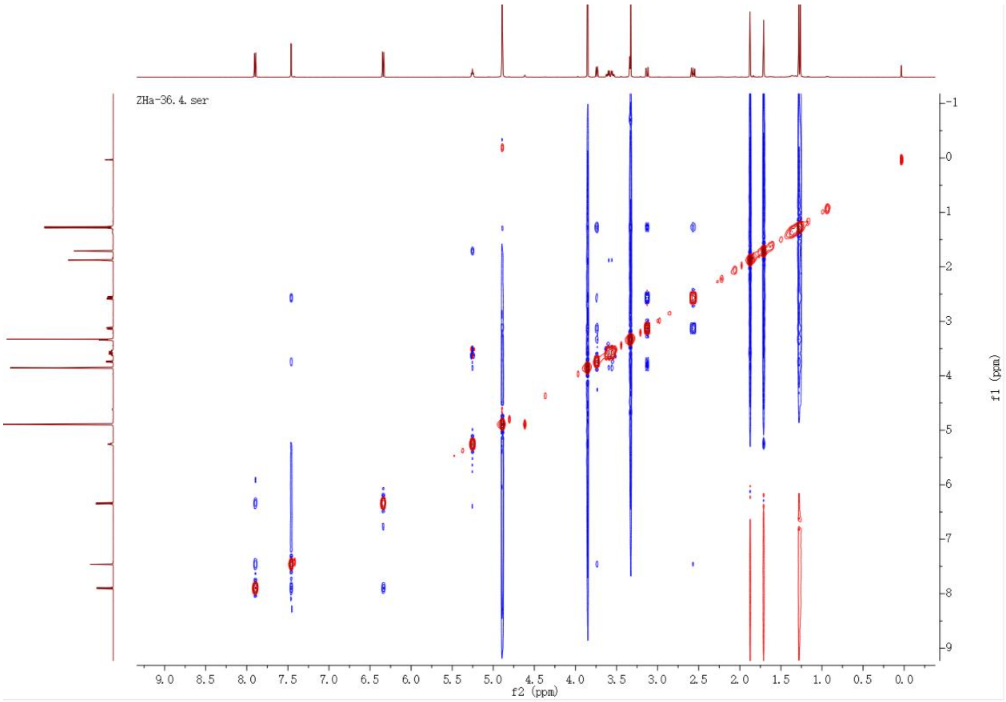

Figure S6. ROESY spectrum of **1**.

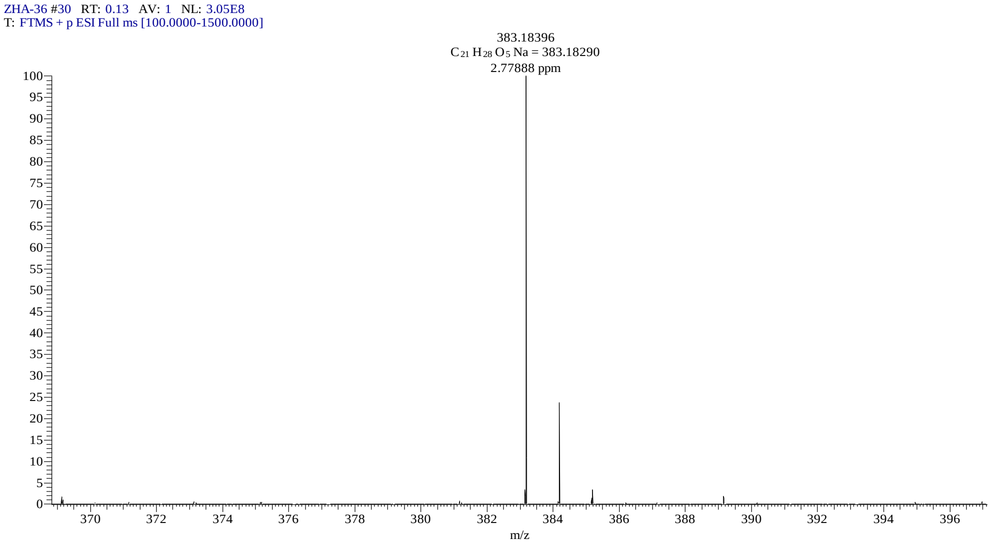

Figure S7. HR-ESI-MS spectrum of **1**.

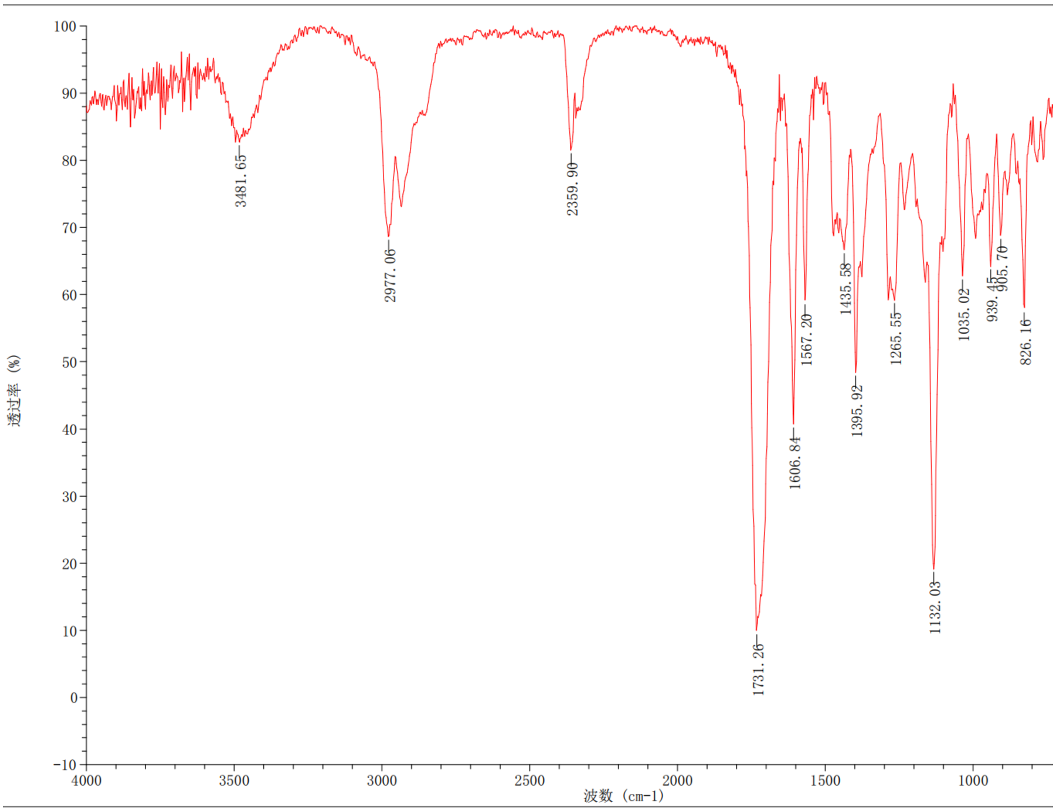

Figure S8. IR spectrum of **1**.

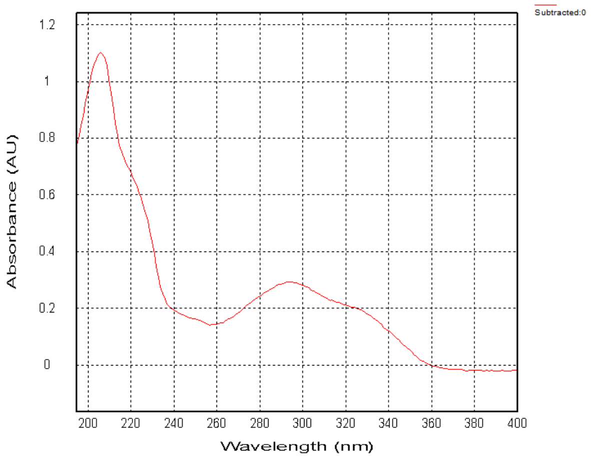

Figure S9. UV spectrum of **1**.

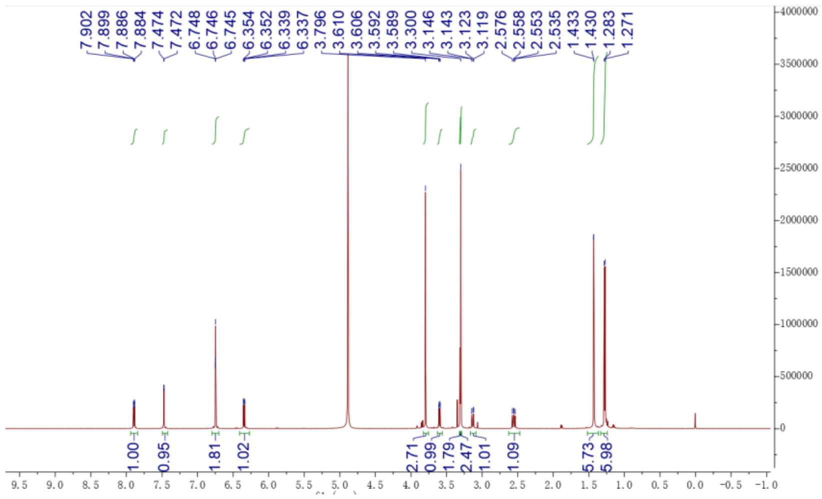

Figure S10. ^{1}H NMR (600 MHz, CD3OD) spectrum of **2**.

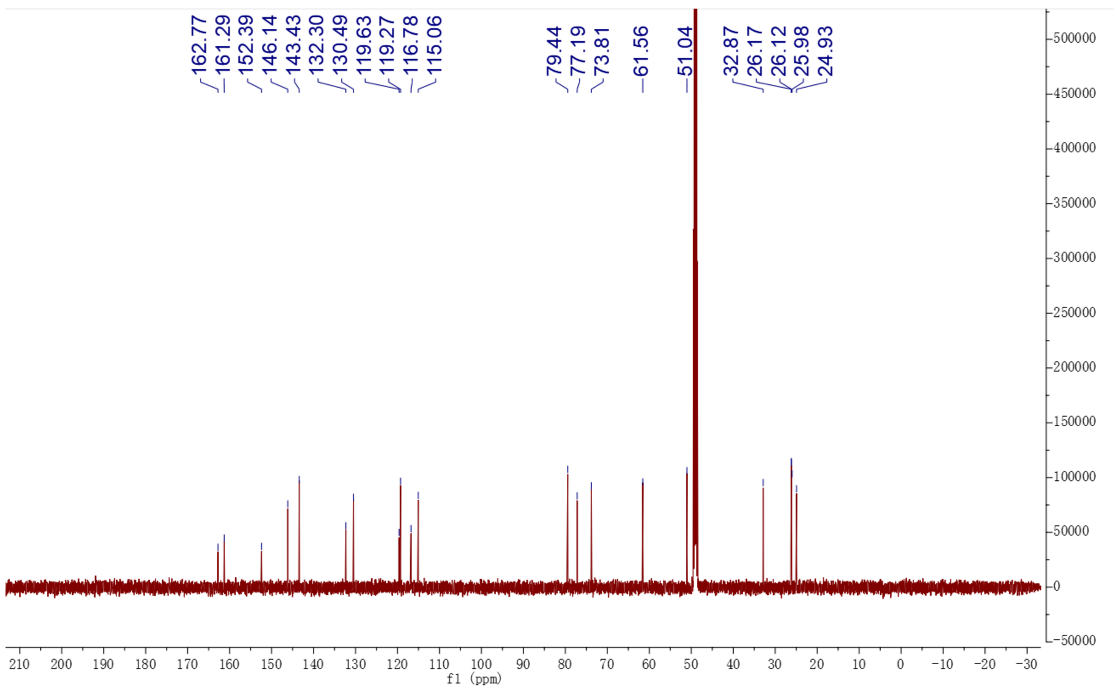

Figure S11. ^{13}C NMR (150 MHz, CD3OD) spectrum of **2**.

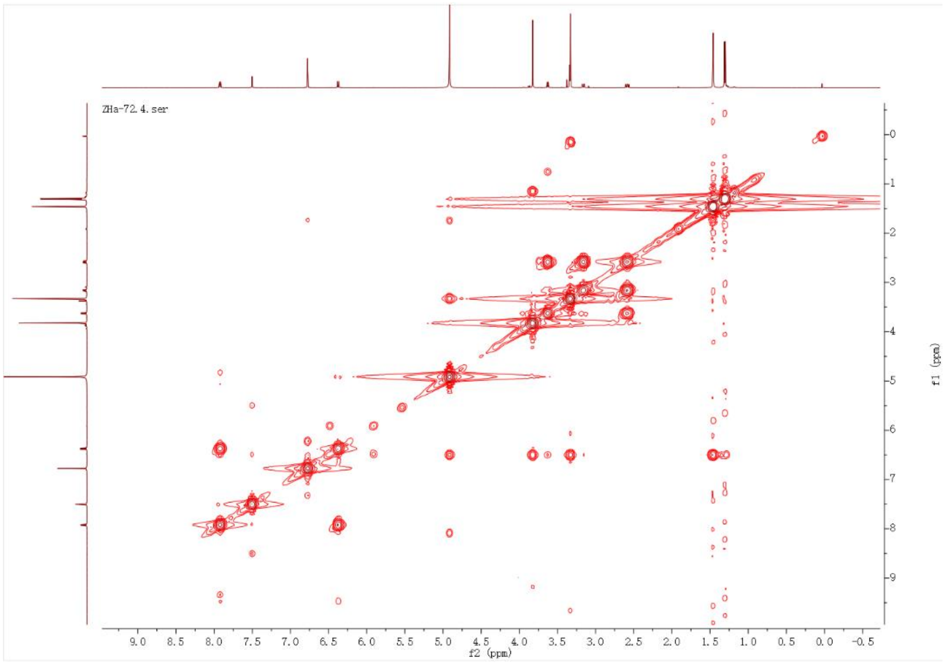

Figure S12. COSY spectrum of **2**.

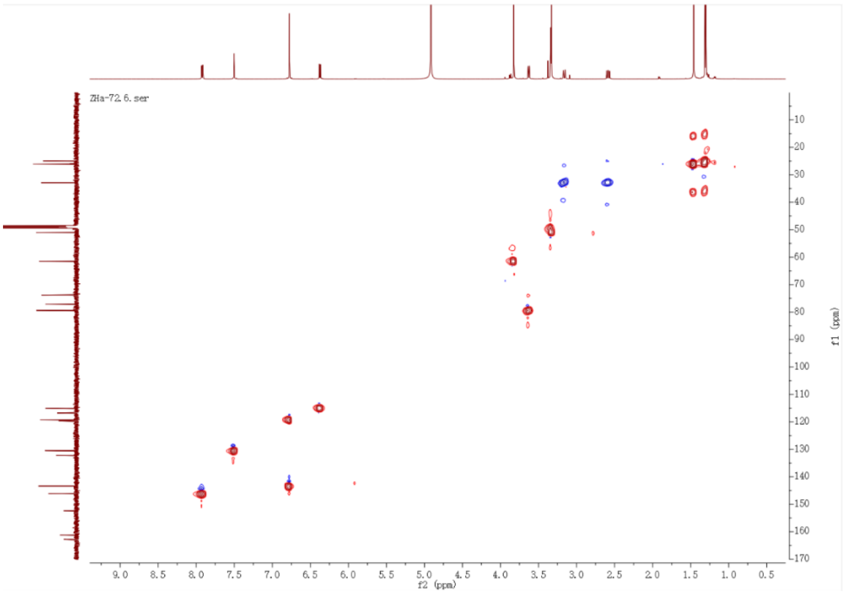

Figure S13. HSQC spectrum of **2**.

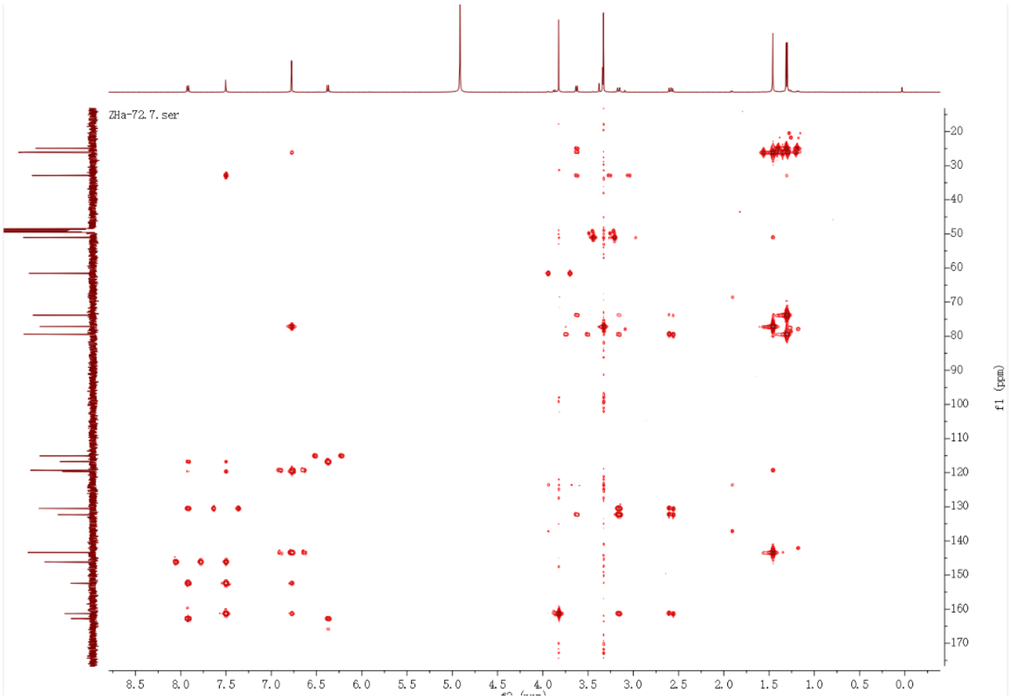

Figure S14. HMBC spectrum of **2**.

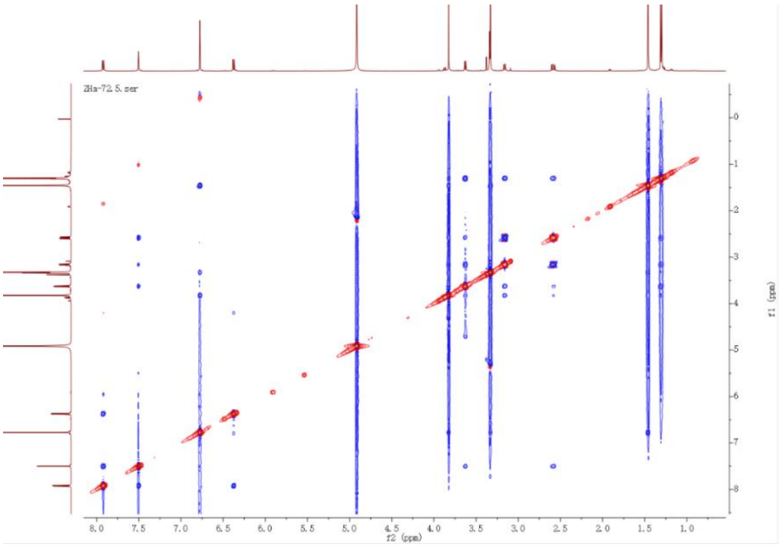

Figure S15. ROESY spectrum of **2**.

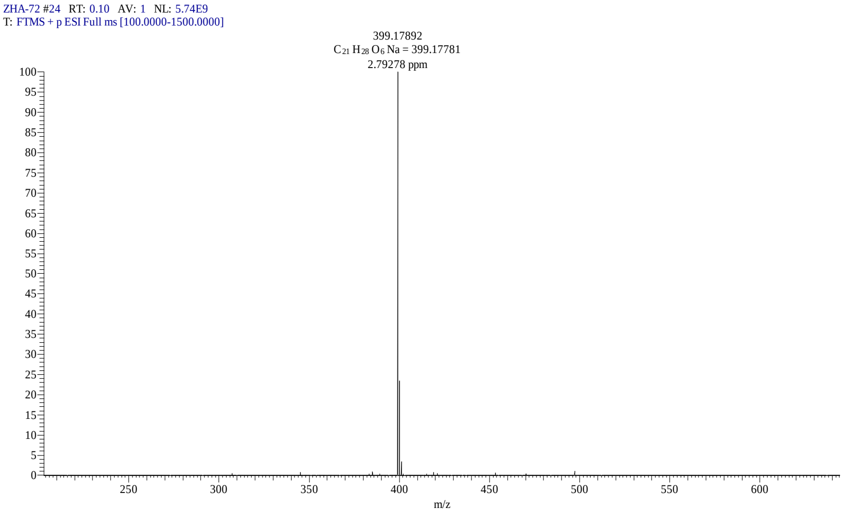

Figure S16. HR-ESI-MS spectrum of **2**.

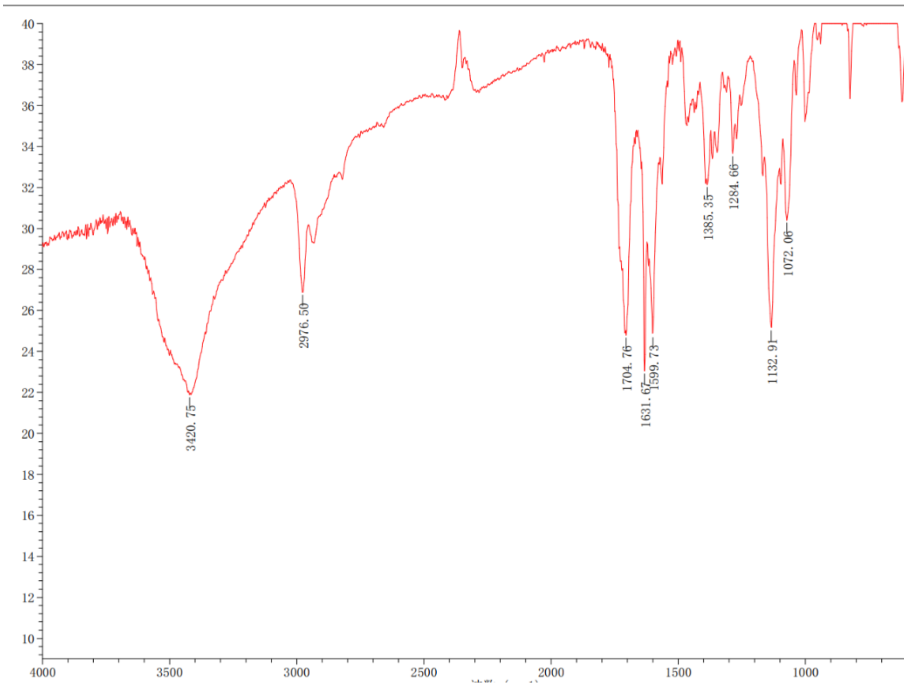

Figure S17. IR spectrum of **2**.

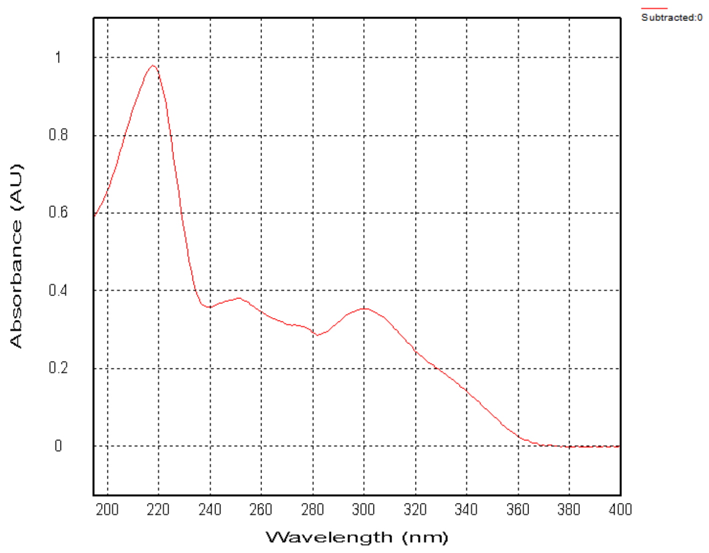

Figure S18. UV spectrum of 2.

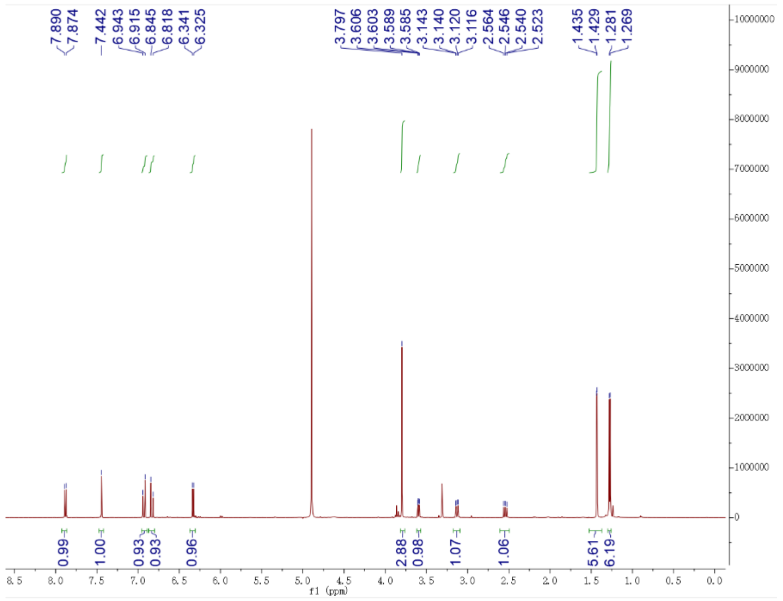

Figure S19. ^{1}H NMR (600 MHz, CD_3OD) spectrum of **3**

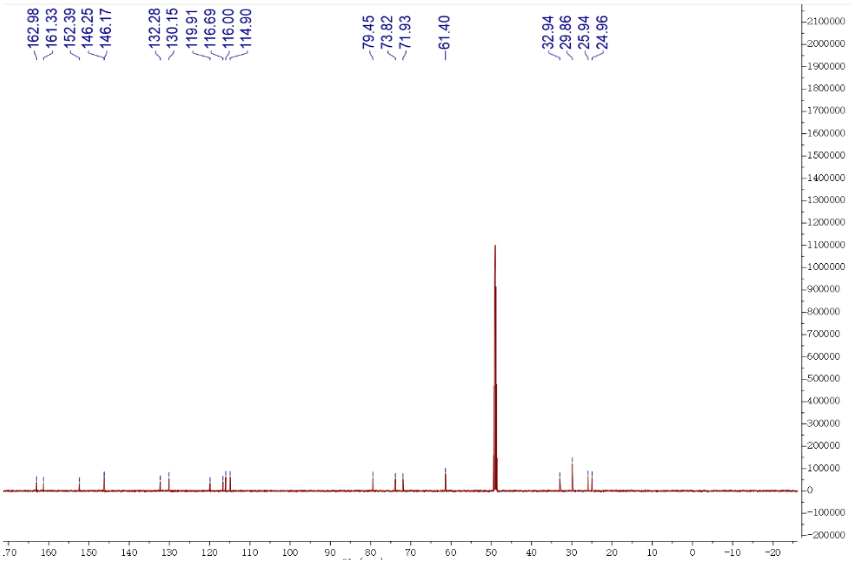

Figure S20. ^{13}C NMR (150 MHz, CD_3OD) spectrum of 3.

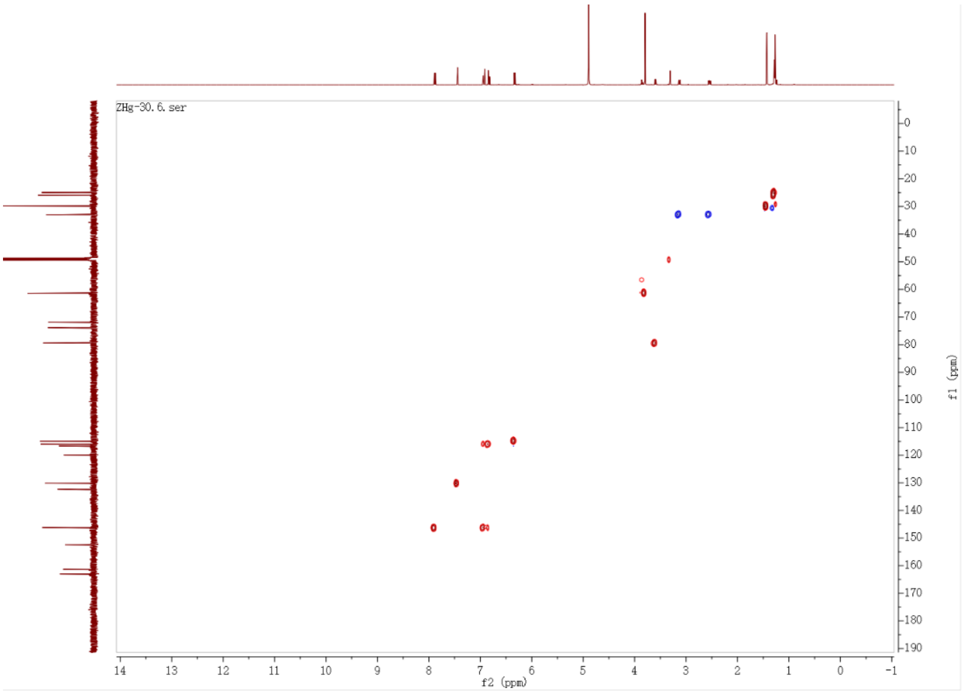

Figure S21. COSY spectrum of **3**.

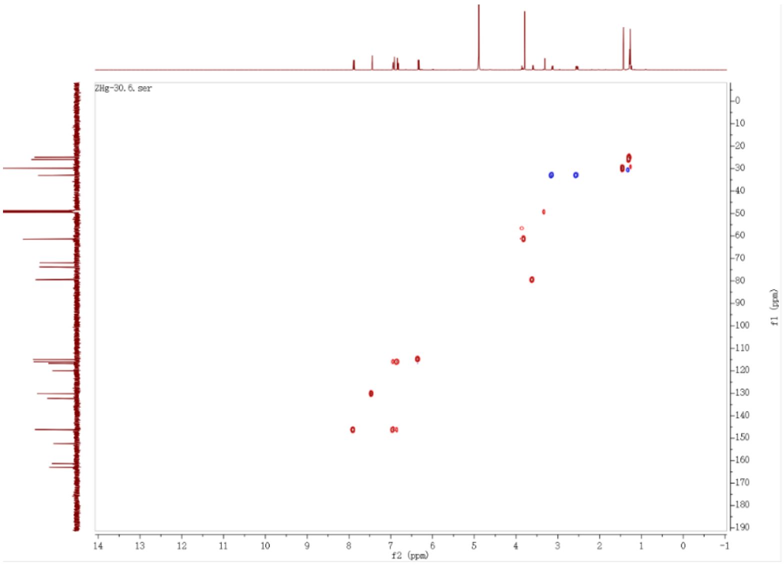

Figure S22. HSQC spectrum of **3.**

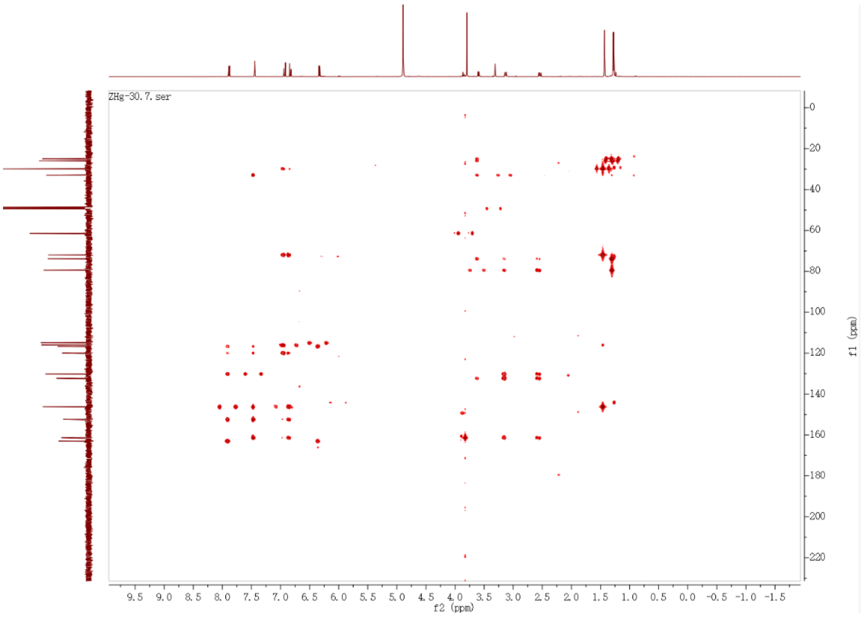

Figure S23. HMBC spectrum of **3**.

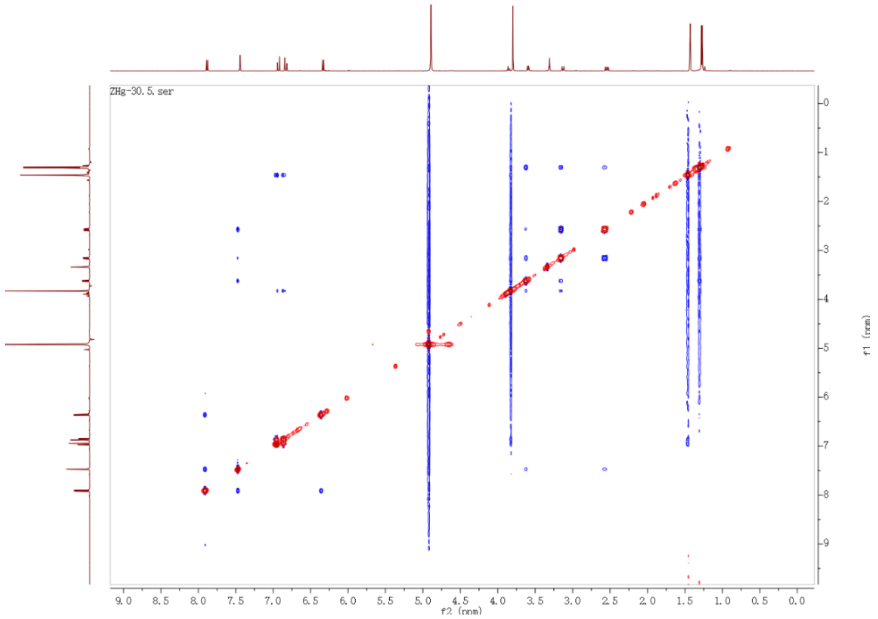

Figure S24. ROESY spectrum of **3**.

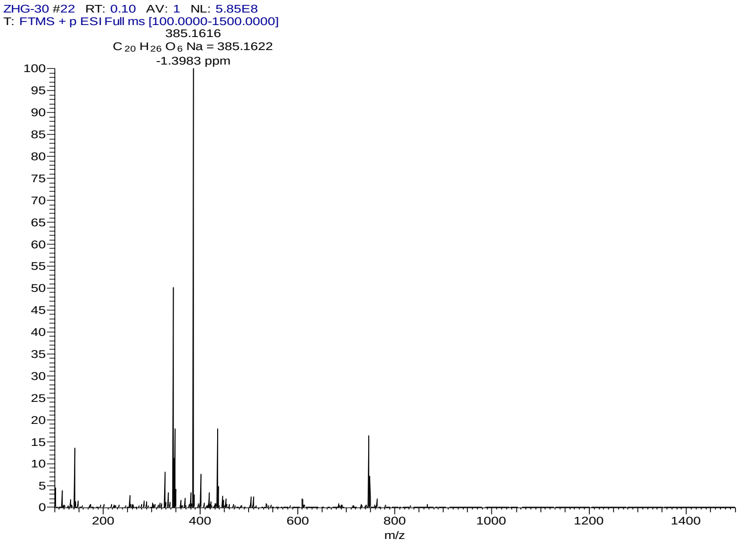

Figure S25. HR-ESI-MS spectrum of **3**.

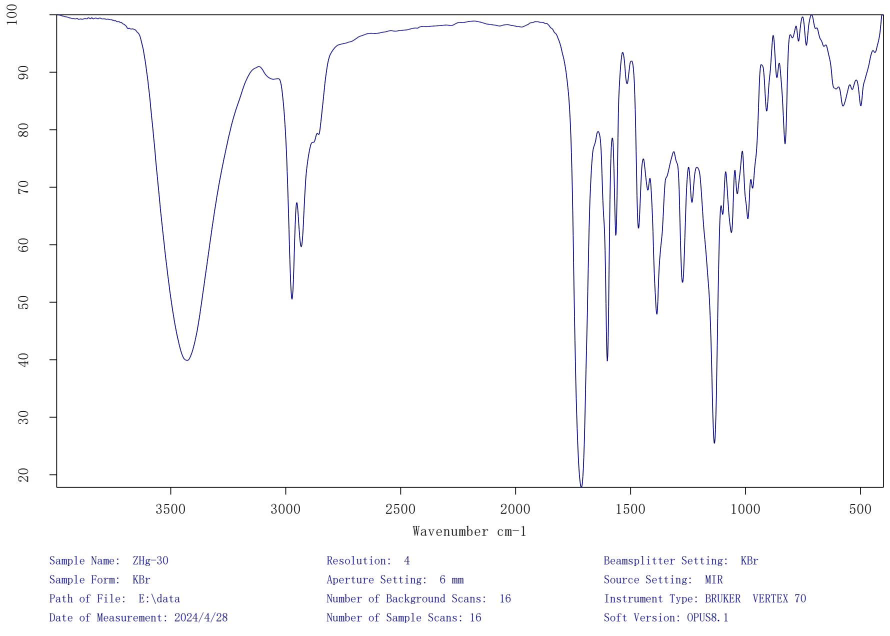

Figure S26. IR spectrum of **3**.

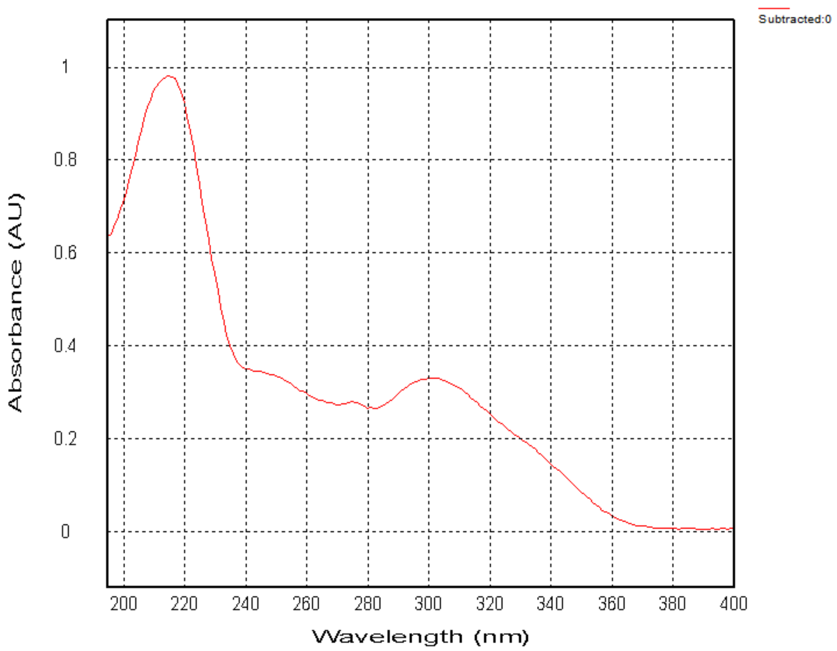

Figure S27. UV spectrum of **3**.

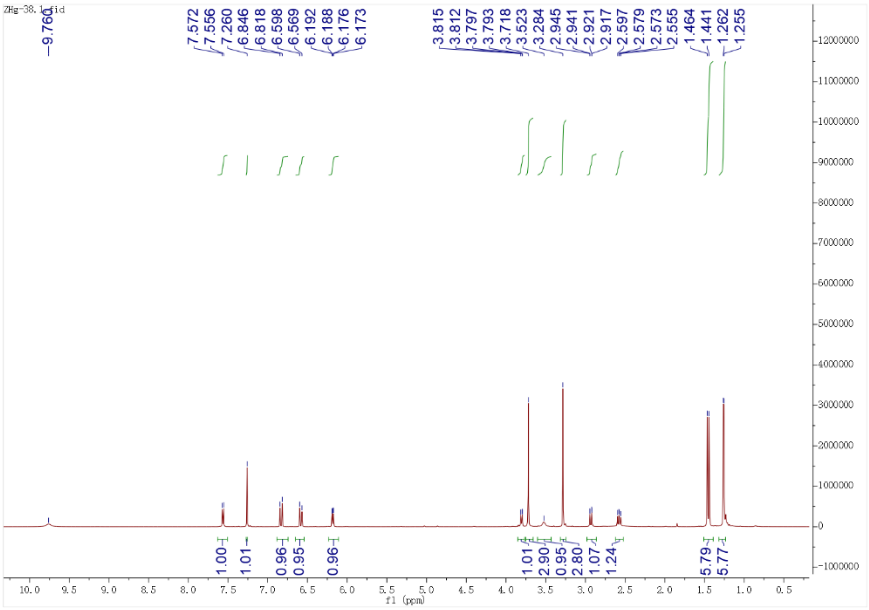

Figure S28. ^{1}H NMR (600 MHz, $CDCl_3$) spectrum of **4**.

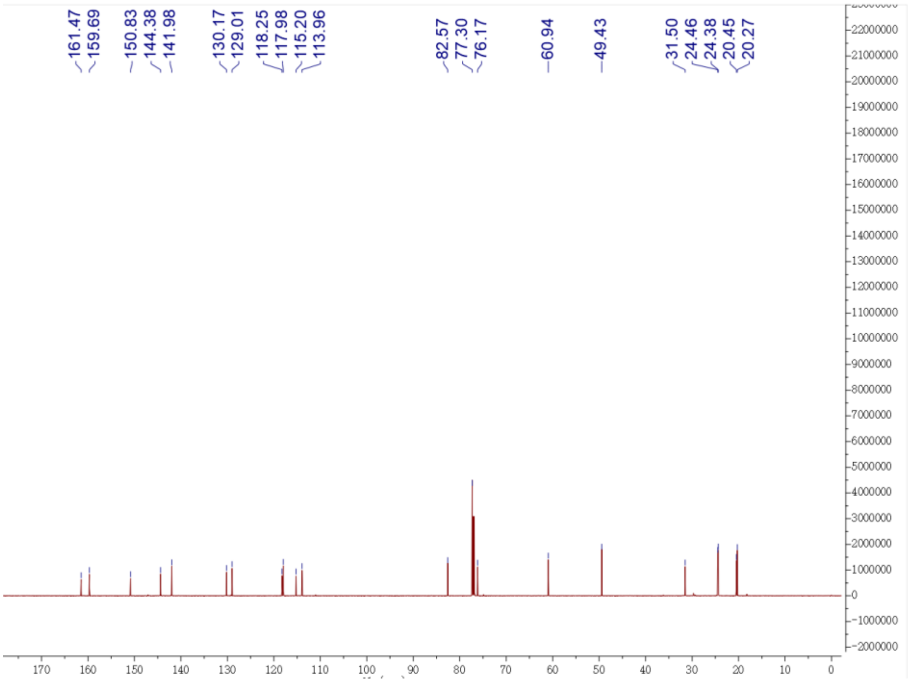

Figure S29. ^{13}C NMR (150 MHz, $CDCl_3$) spectrum of **4**.

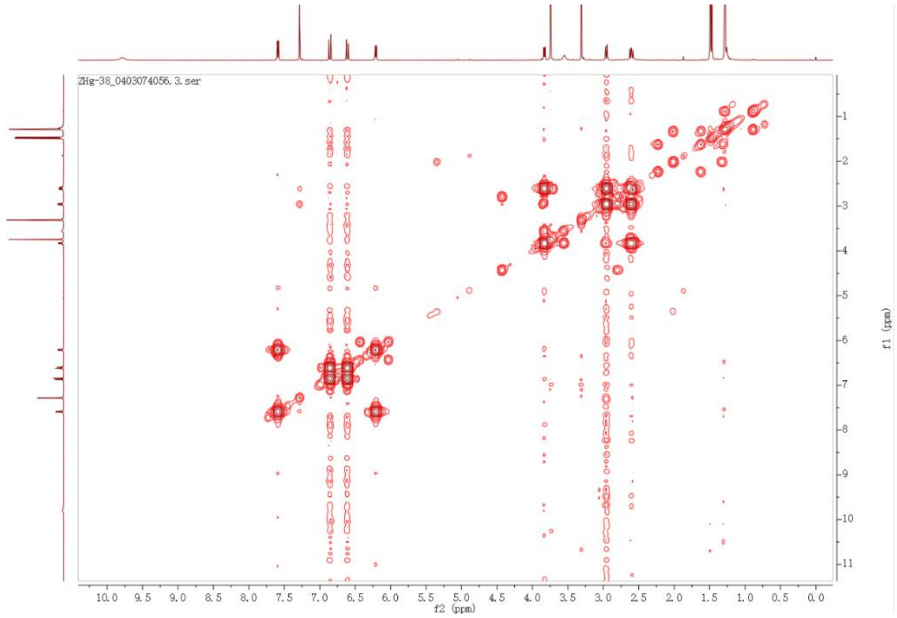

Figure S30. COSY spectrum of **4**.

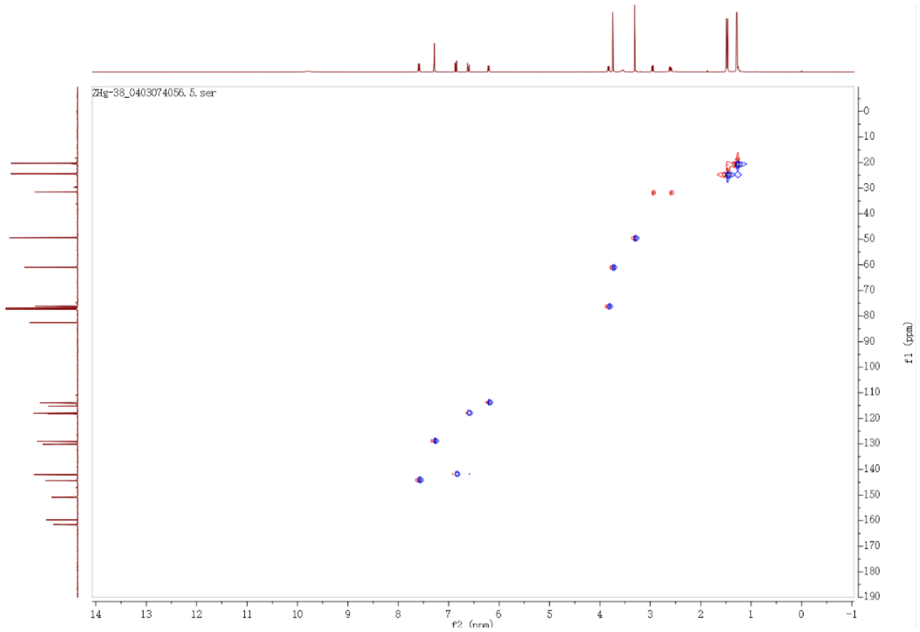

Figure S31. HSQC spectrum of **4**.

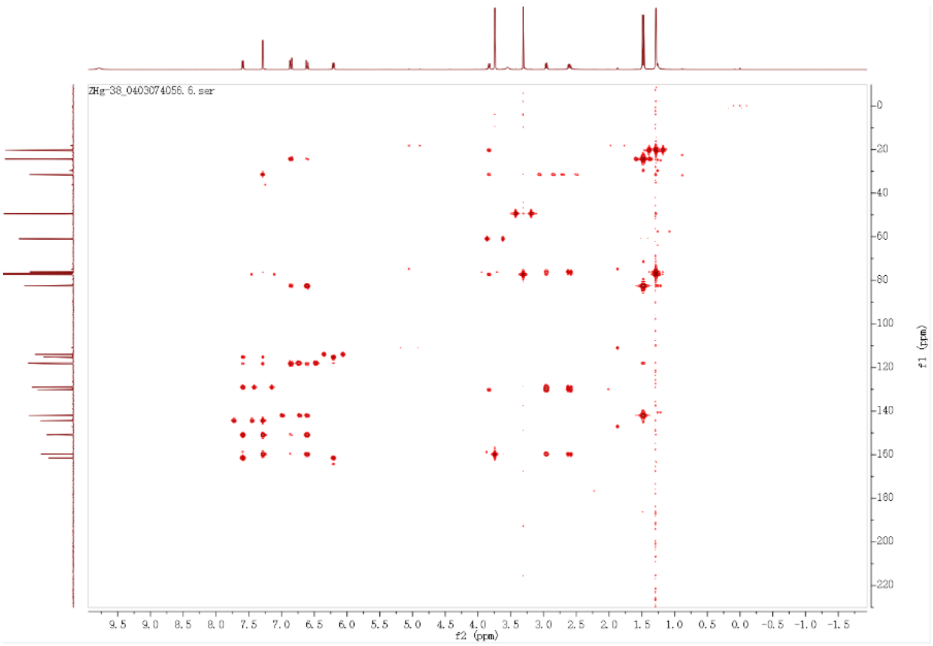

Figure S32. HMBC spectrum of **4**.

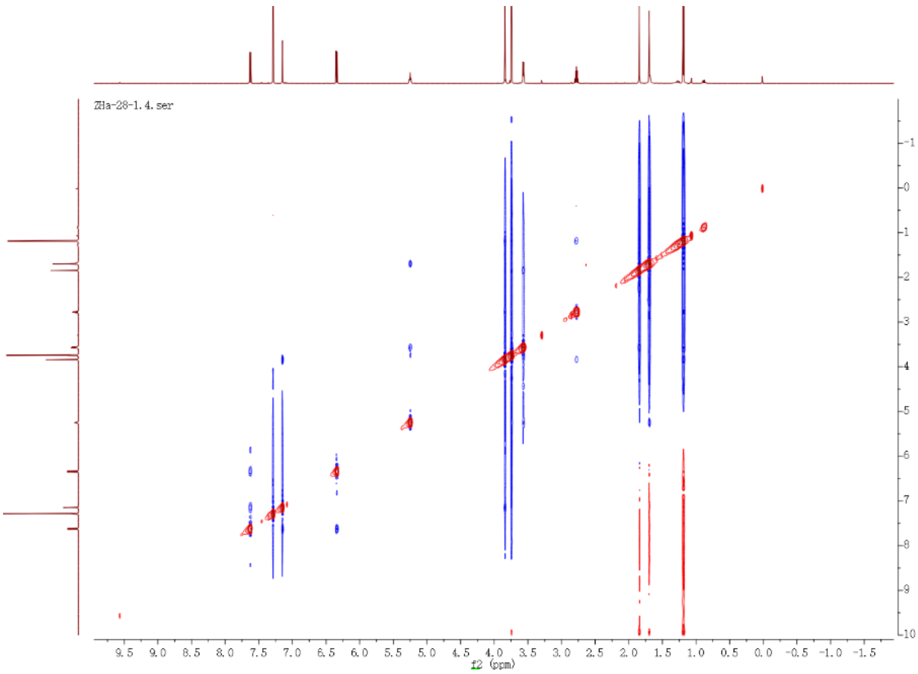

Figure S33. ROESY spectrum of **4**.

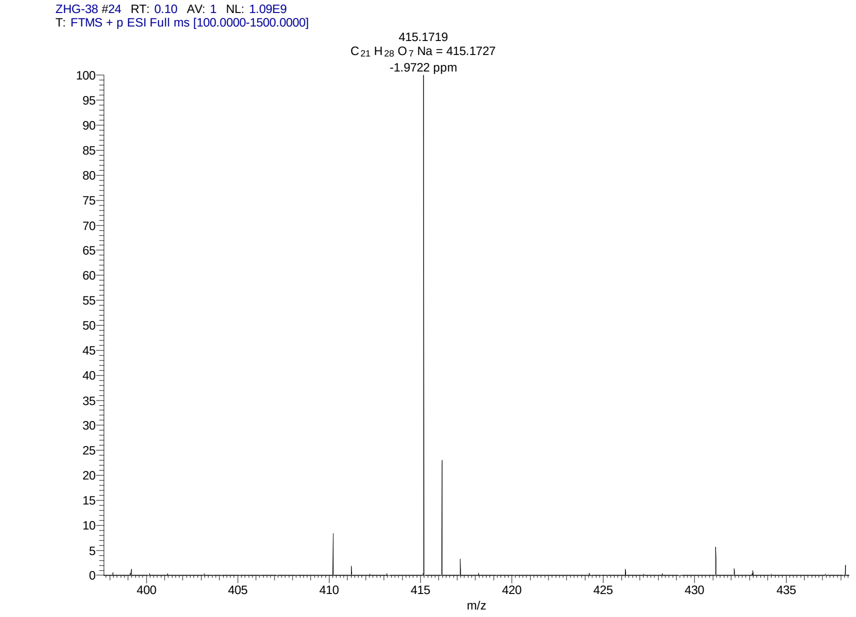

Figure S34. HR-ESI-MS spectrum of **4**.

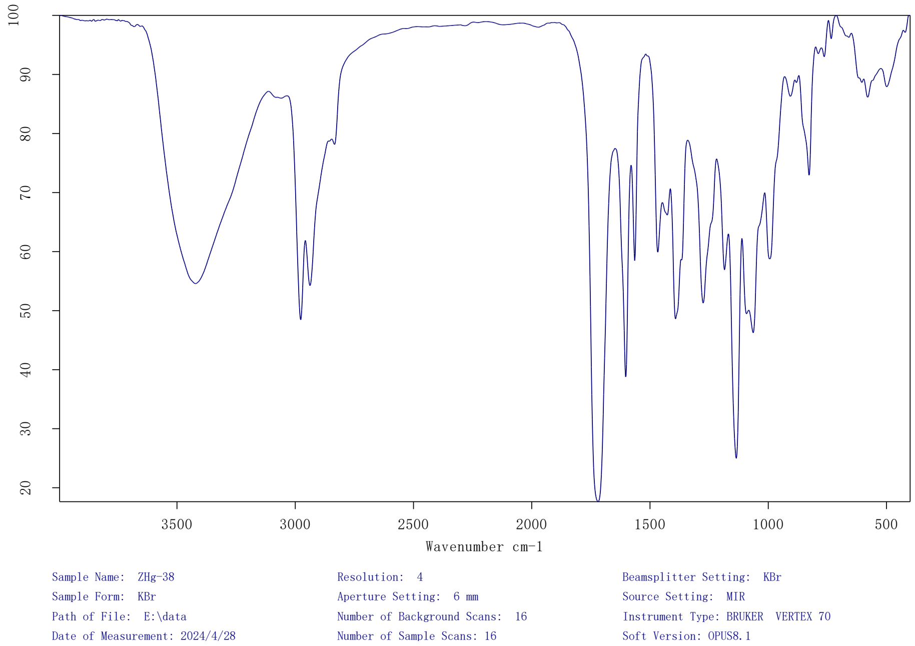

Figure S35. IR spectrum of **4**.

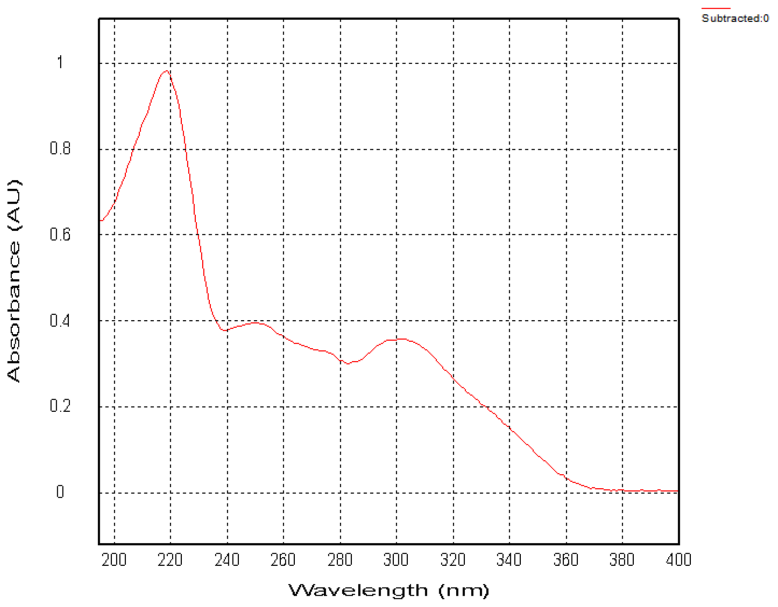

Figure S36. UV spectrum of **4**.

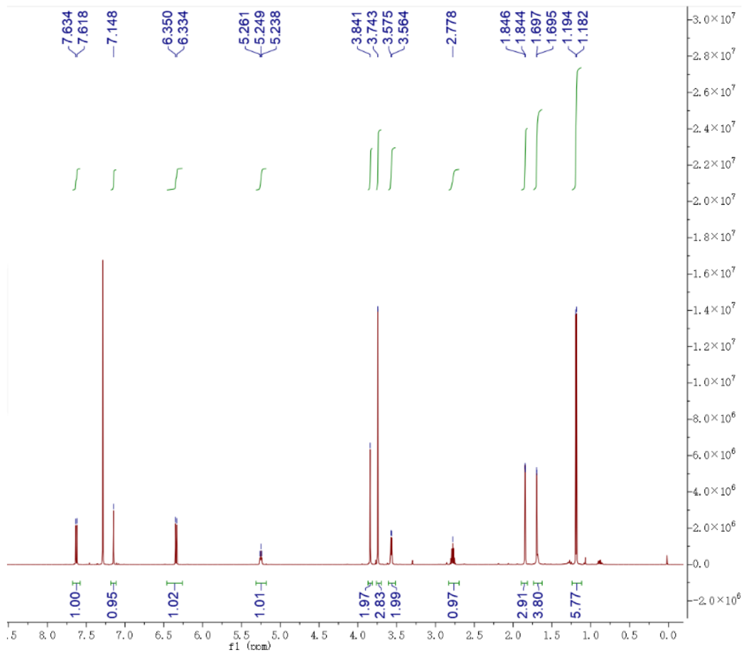

Figure S37. ^{1}H NMR (600 MHz, CD_3OD) spectrum of **5**.

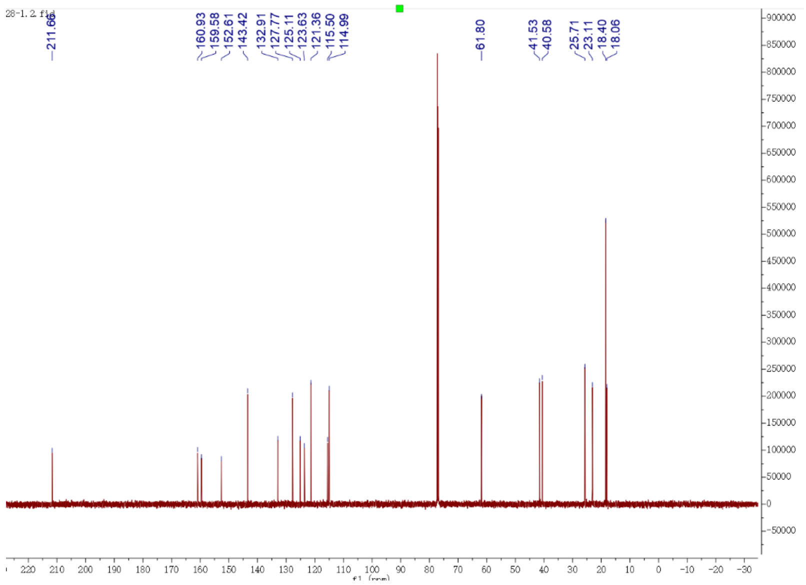

Figure S38. ^{13}C NMR (150 MHz, CD_3OD) spectrum of **5.**

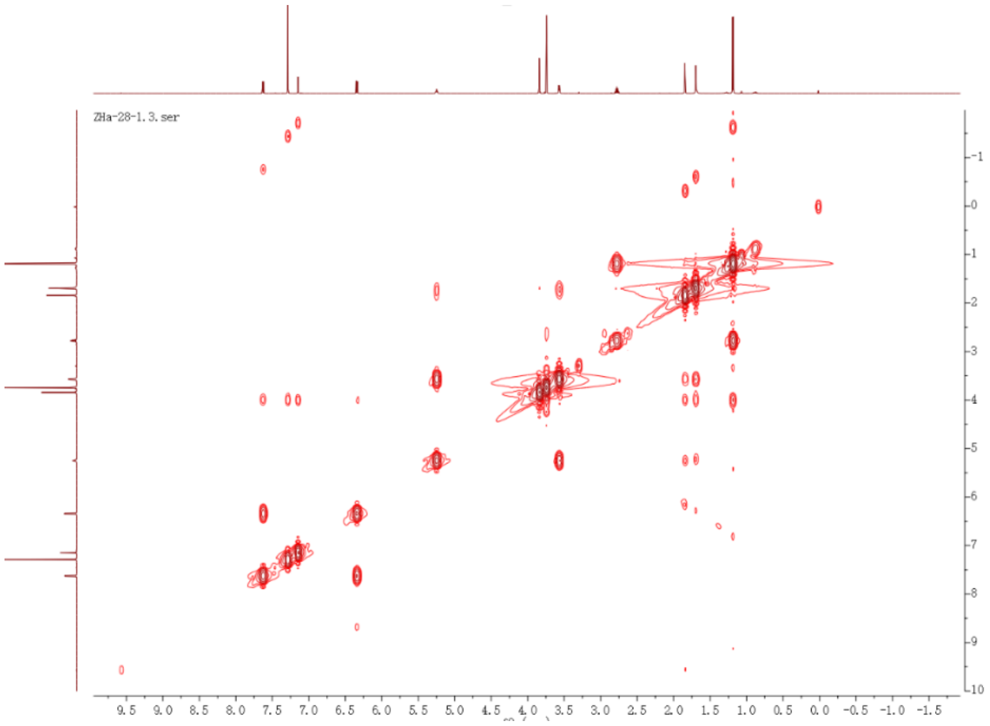

Figure S39. COSY spectrum of **5**.

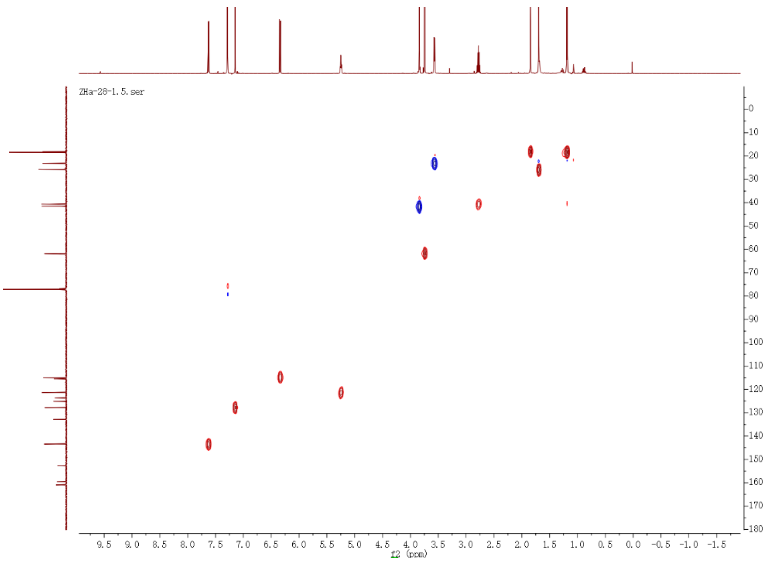

Figure S40. HSQC spectrum of **5**.

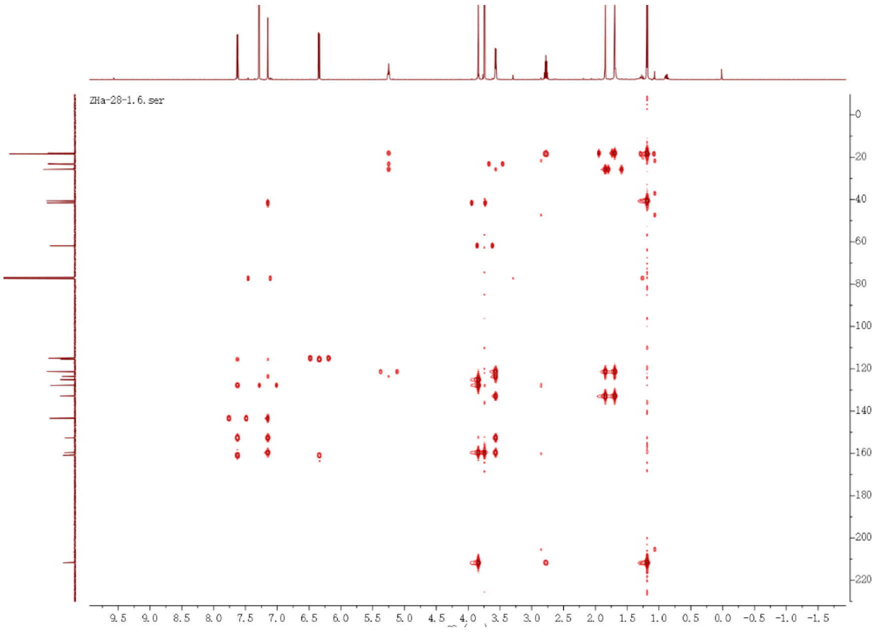

Figure S41. HMBC spectrum of **5**.

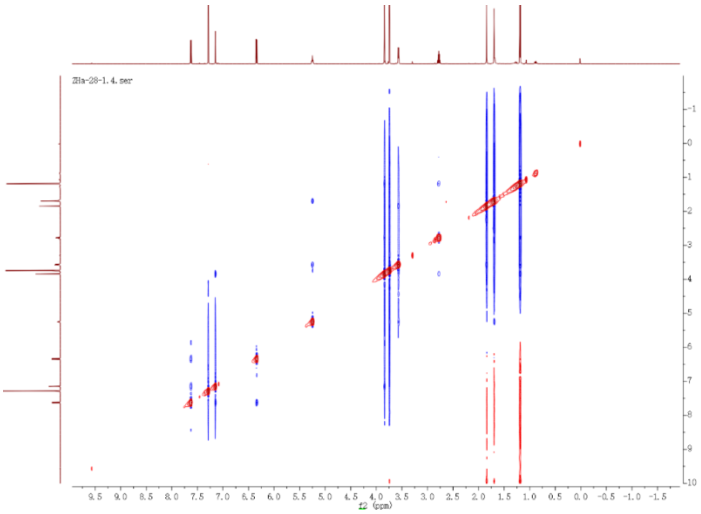

Figure S42. ROESY spectrum of **5**.

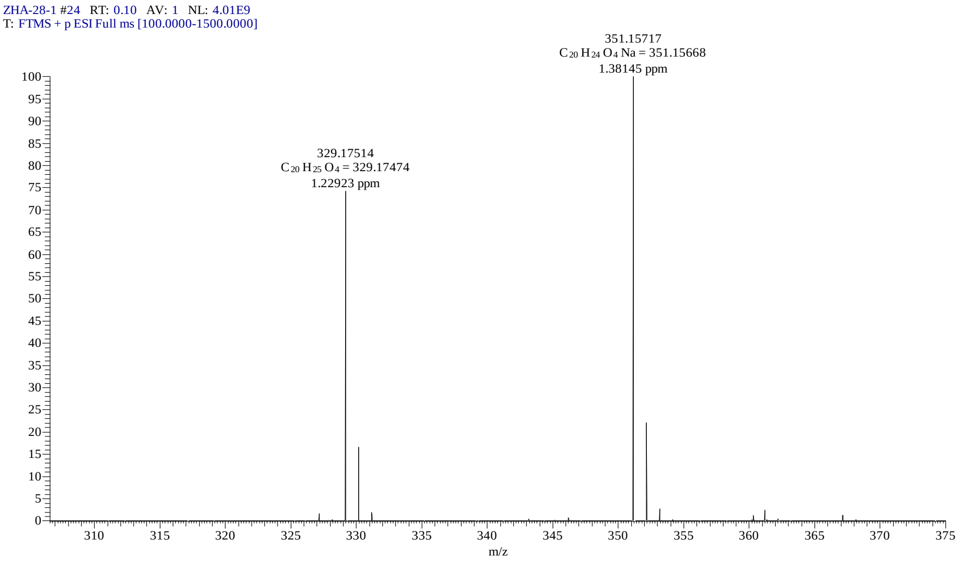

Figure S43. HR-ESI-MS spectrum of **5**.

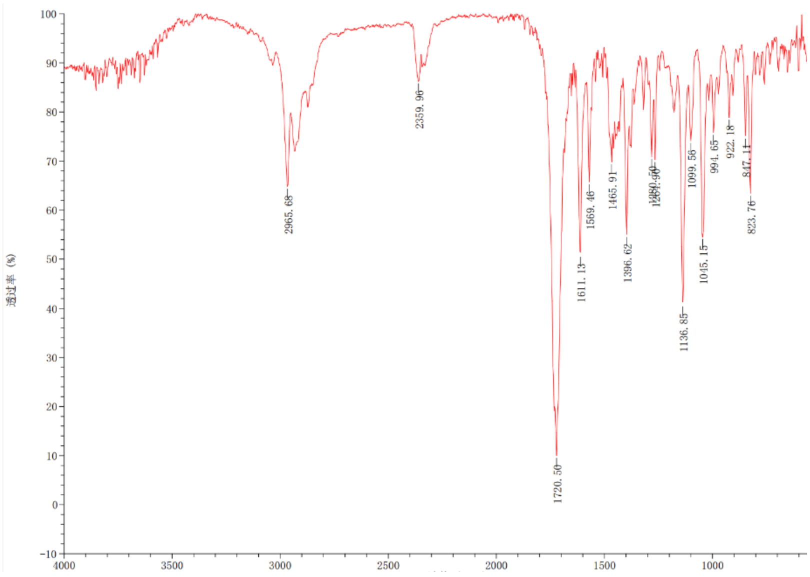

Figure S44. IR spectrum of **5**.

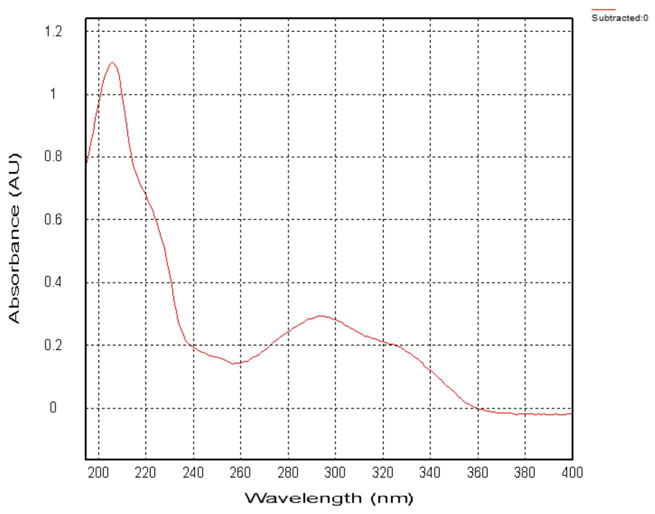

Figure S45. UV spectrum of **5**.

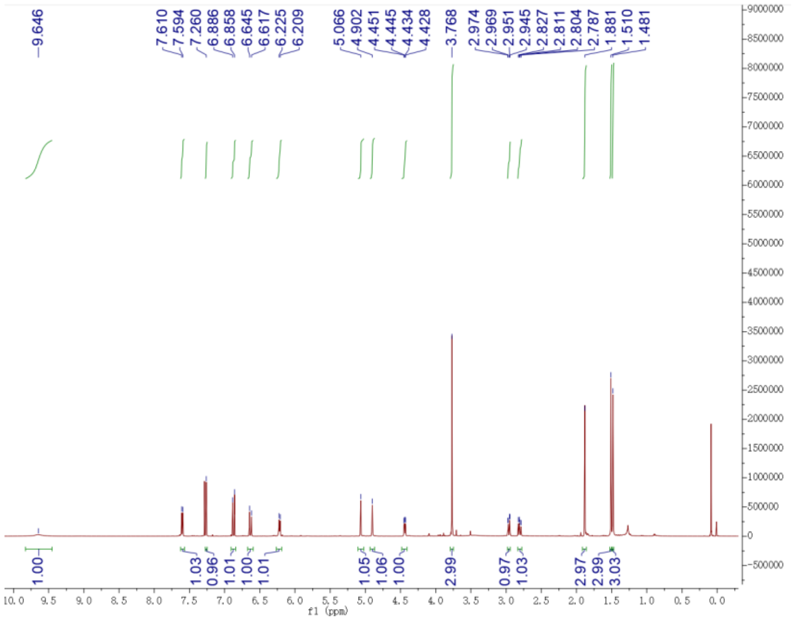

Figure S46. ^{1}H NMR (600 MHz, CD_3OD) spectrum of **6**.

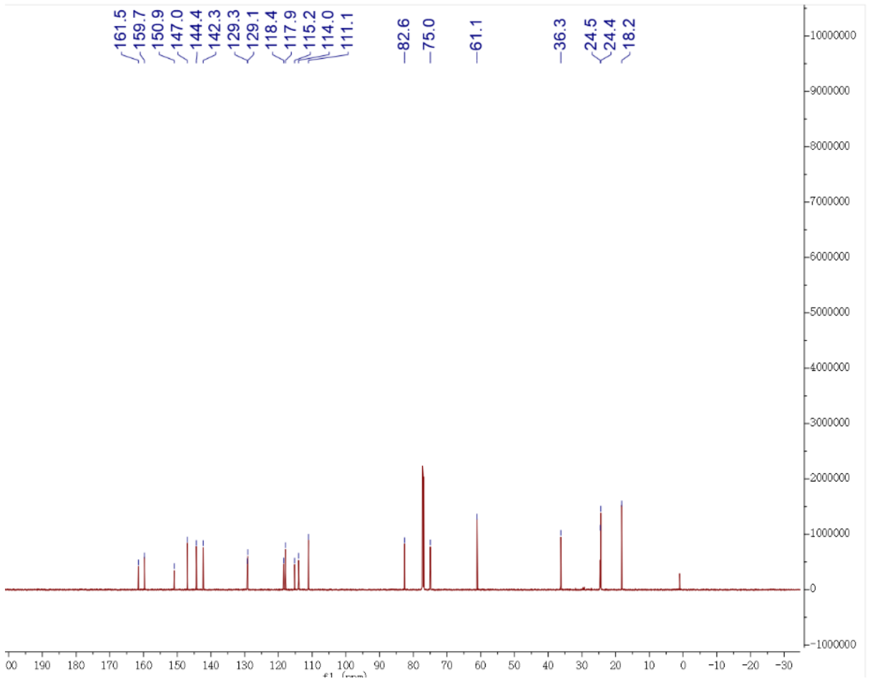

Figure S47. ^{13}C NMR (150 MHz, CD_3OD) spectrum of **6**.

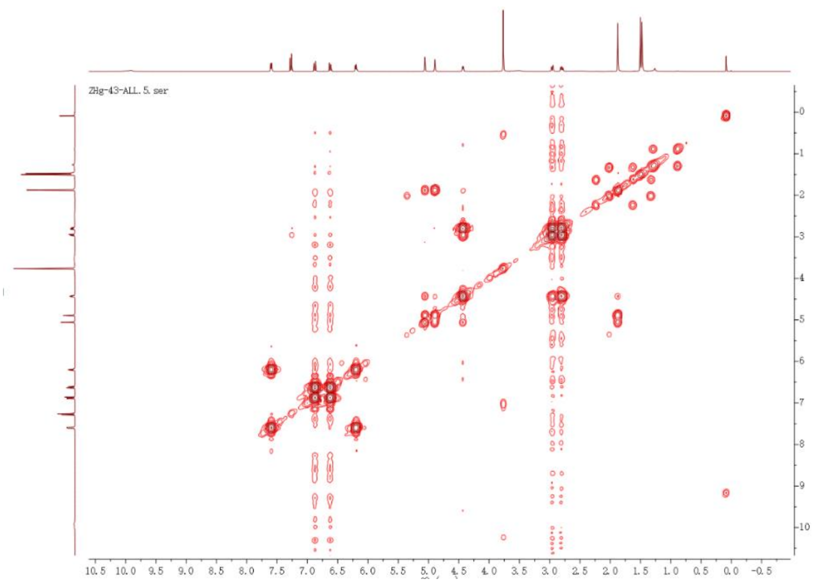

Figure S48. COSY spectrum of **6**.

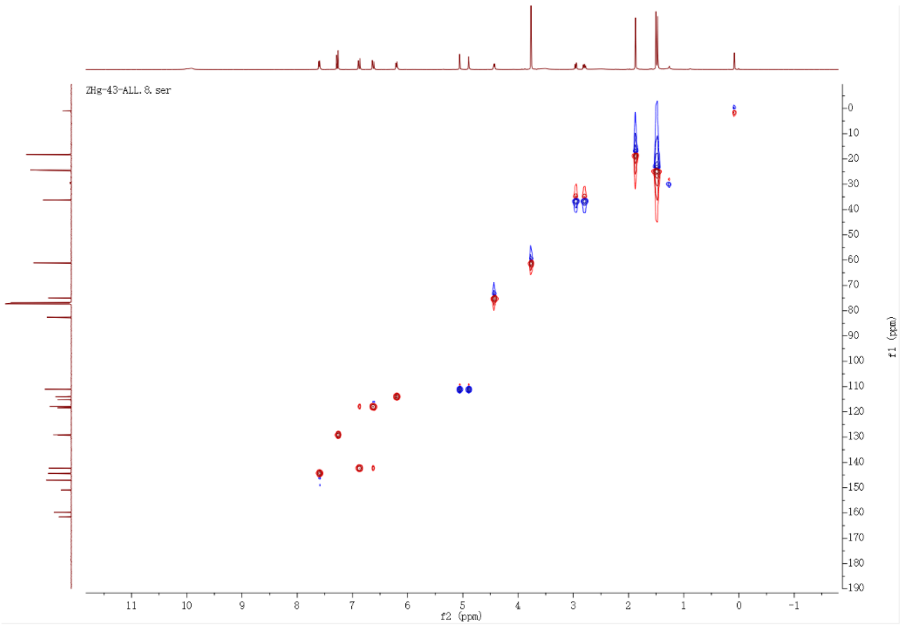

Figure S49. HSQC spectrum of **6**.

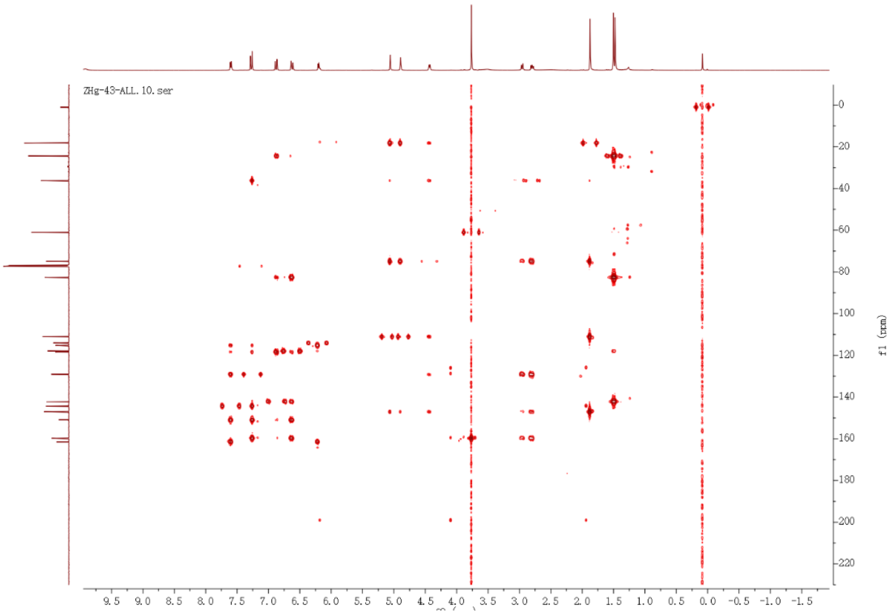

Figure S50. HMBC spectrum of **6**.

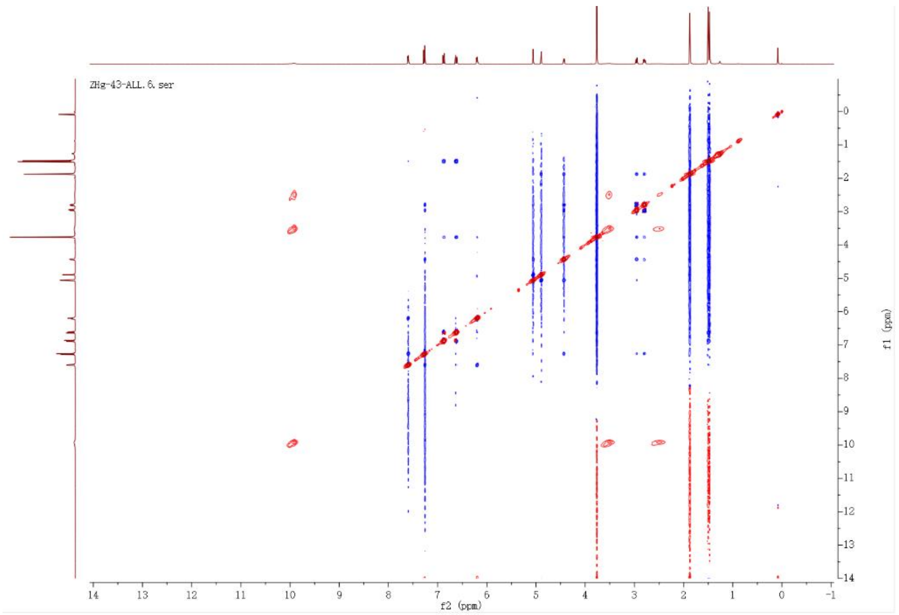

Figure S51. ROESY spectrum of **6**.

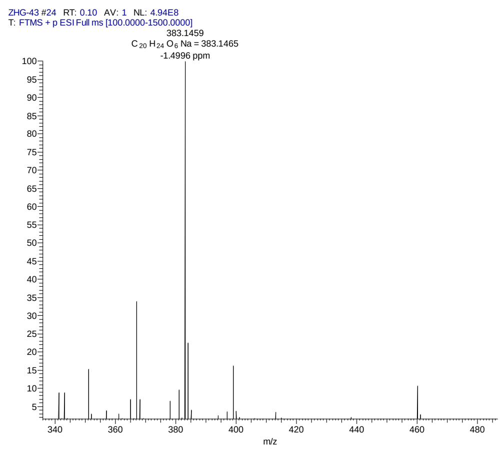

Figure S52. HR-ESI-MS spectrum of **6**.

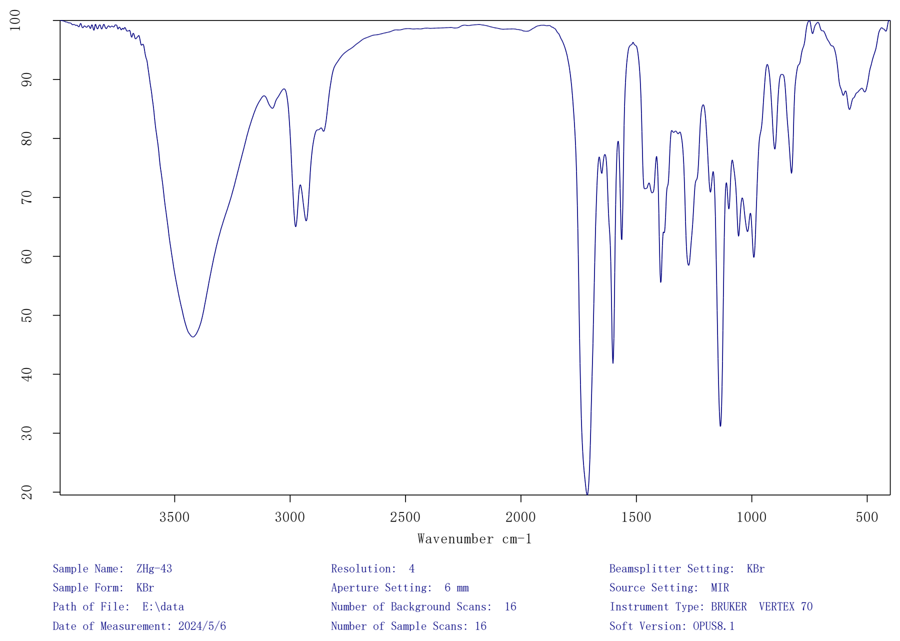

Figure S53. IR spectrum of **6**.

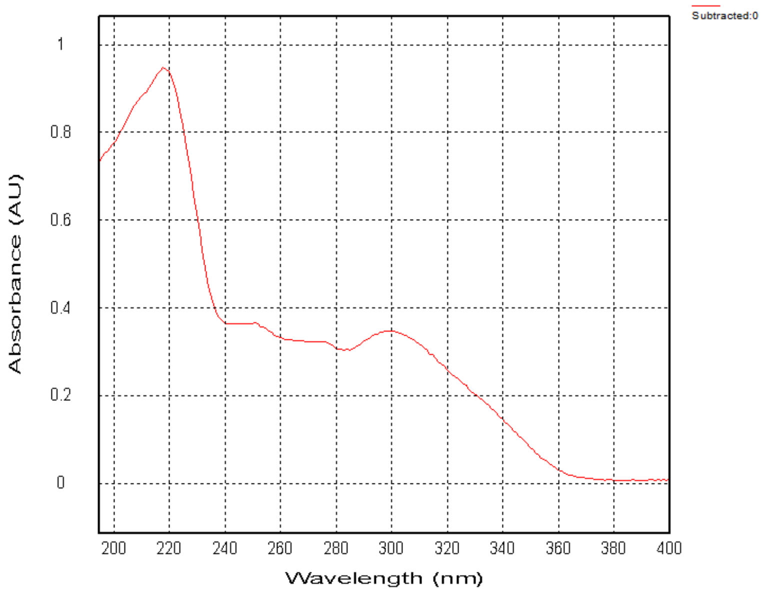

Figure S54. UV spectrum of **6**.

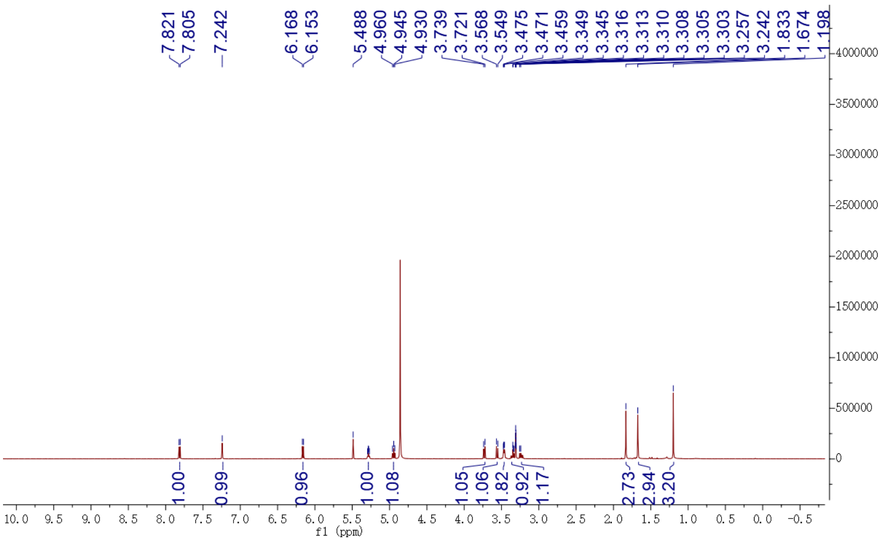

Figure S55. ^{1}H NMR (600 MHz, CD_3OD) spectrum of **7**.

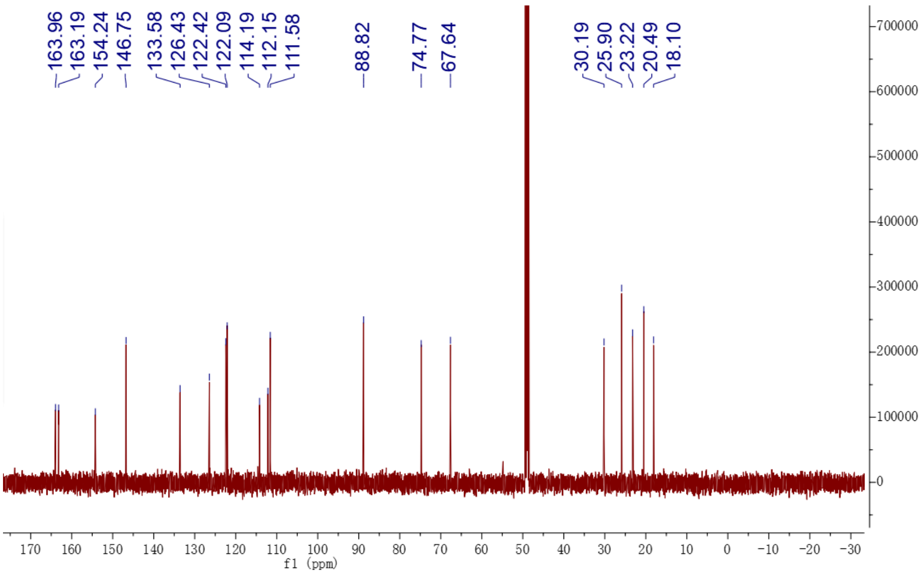

Figure S56. ^{13}C NMR (150 MHz, CD_3OD) spectrum of **7**.

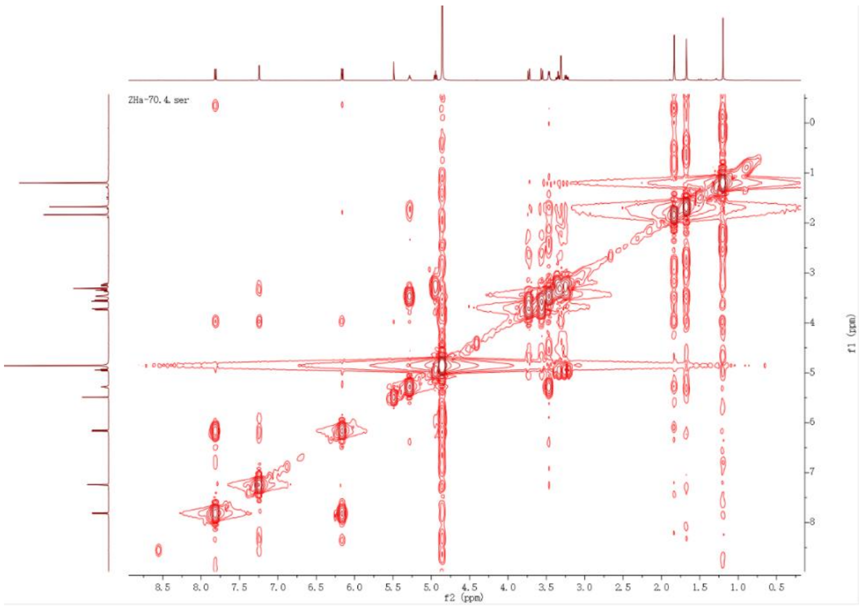

Figure S57. COSY spectrum of **7**.

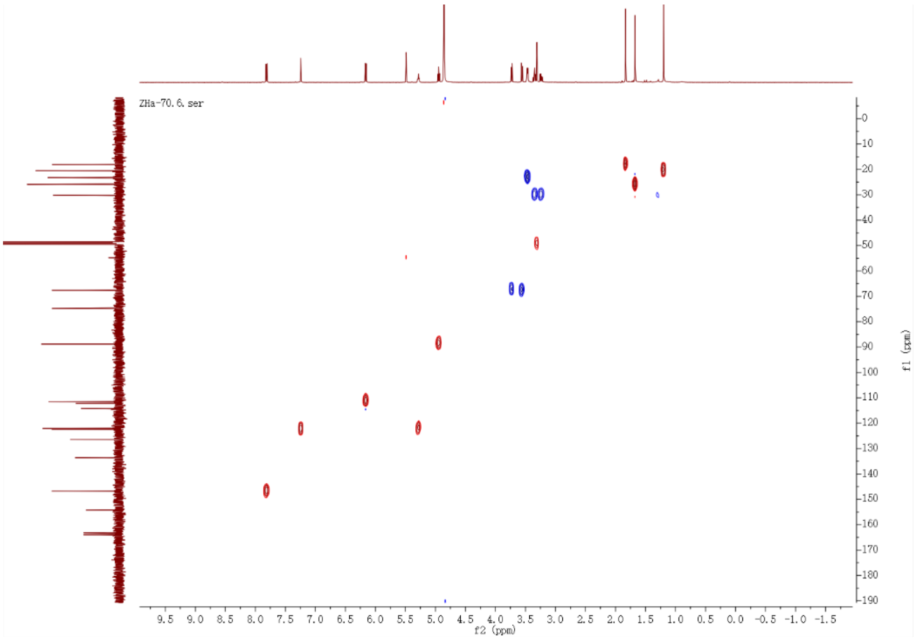

Figure S58. HSQC spectrum of **7**.

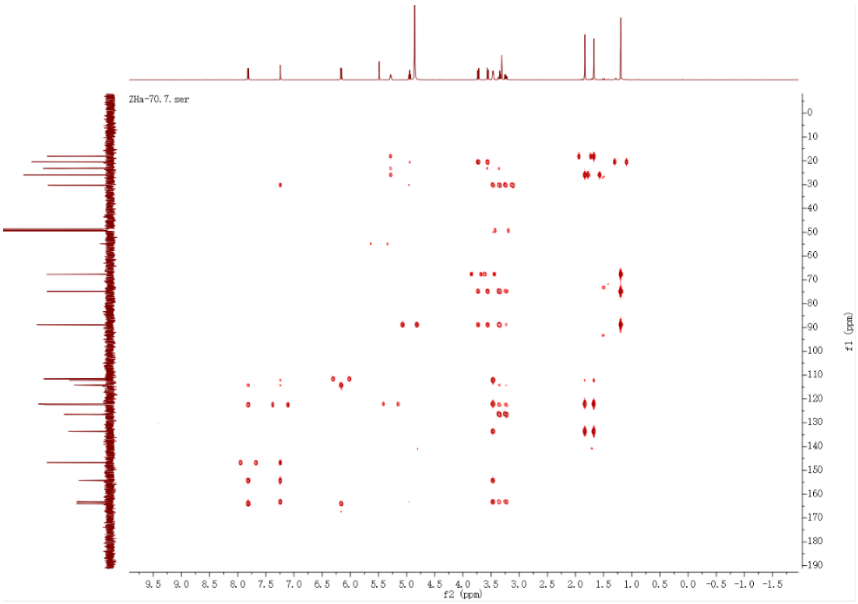

Figure S59. HMBC spectrum of **7**.

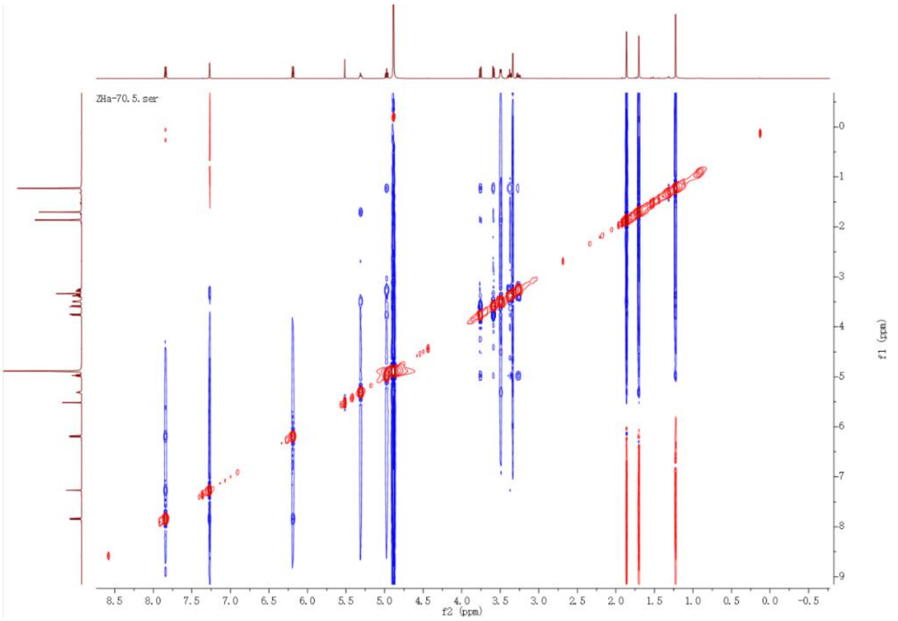

Figure S60. ROESY spectrum of **7**.

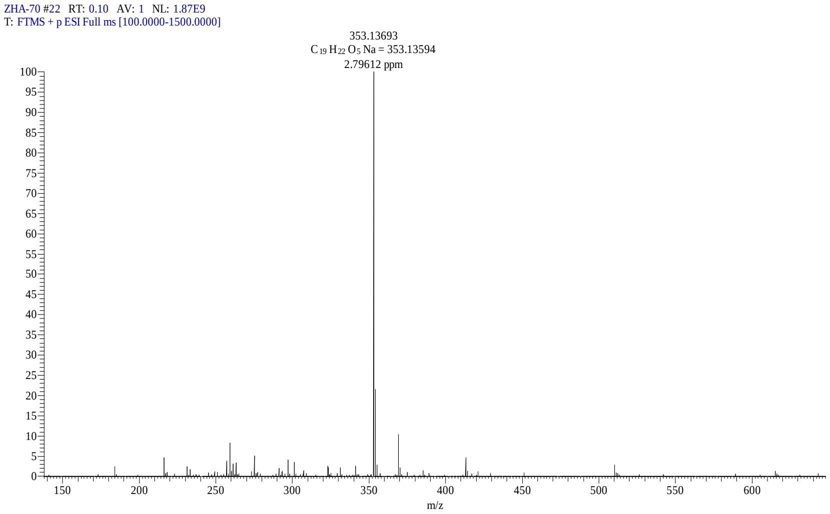

Figure S61. HR-ESI-MS spectrum of **7**.

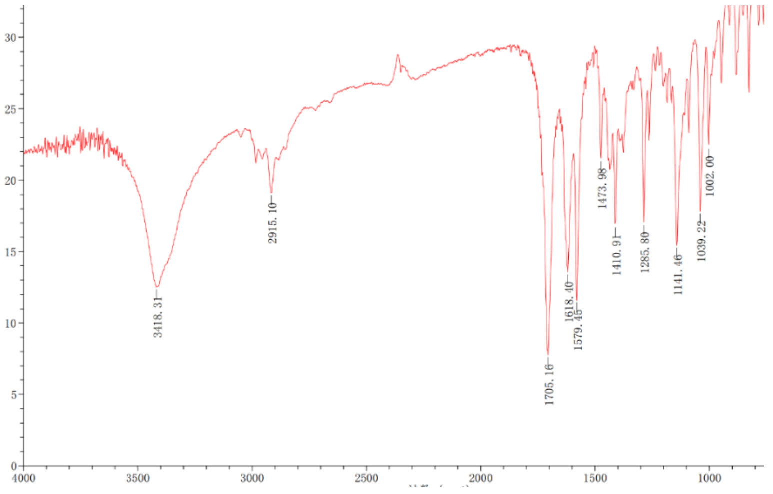

Figure S62. IR spectrum of 7.

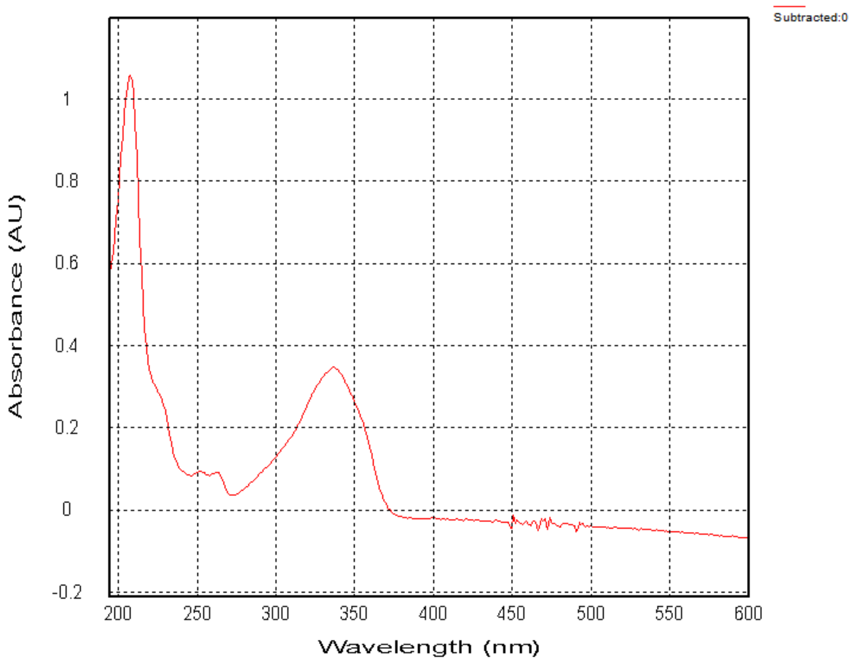

Figure S63. UV spectrum of **7**.

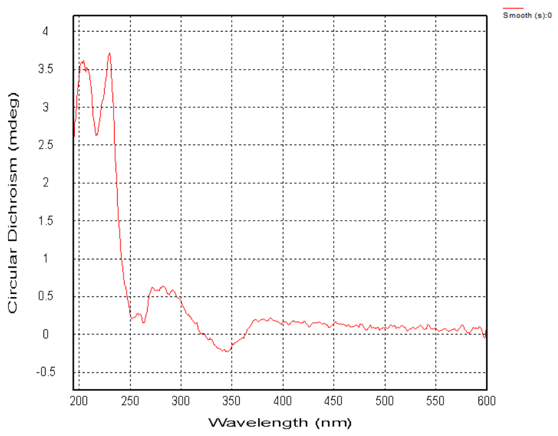

Figure S64. CD spectrum of **7**.

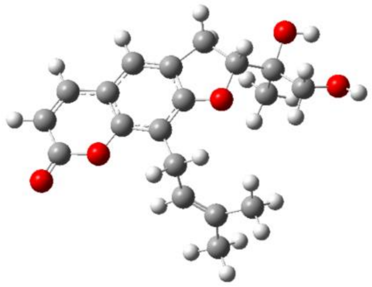

Figure S65. B3LYP/6-311+g (d) optimized lowest energy 3D conformers of **7**.

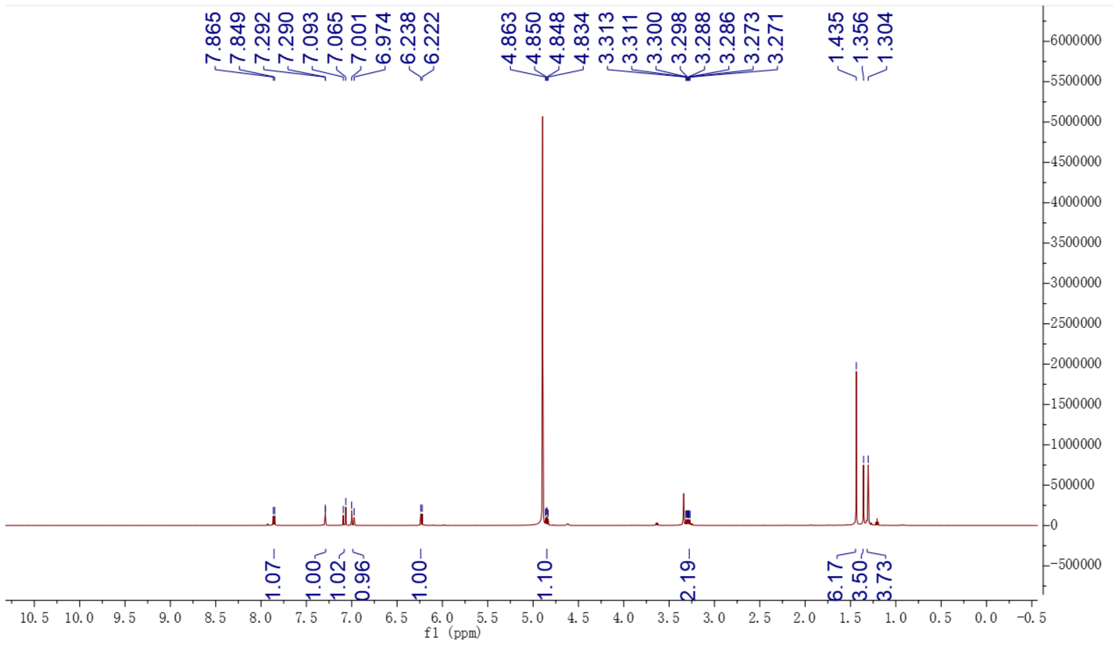

Figure S66. ^{1}H NMR (600 MHz, CD_3OD) spectrum of **8**.

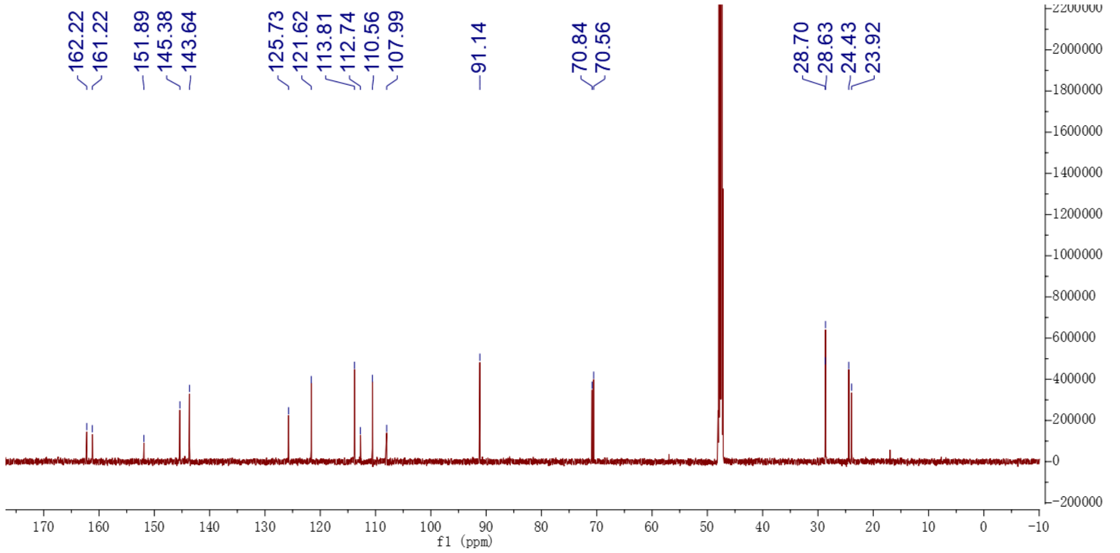

Figure S67. ^{13}C NMR (150 MHz, CD_3OD) spectrum of **8**.

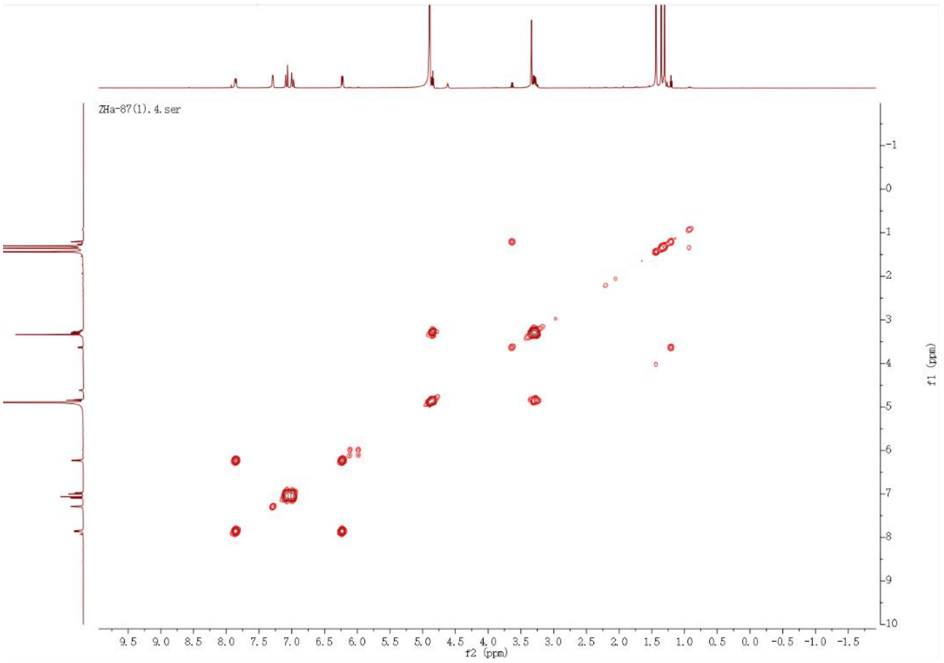

Figure S68. COSY spectrum of **8**.

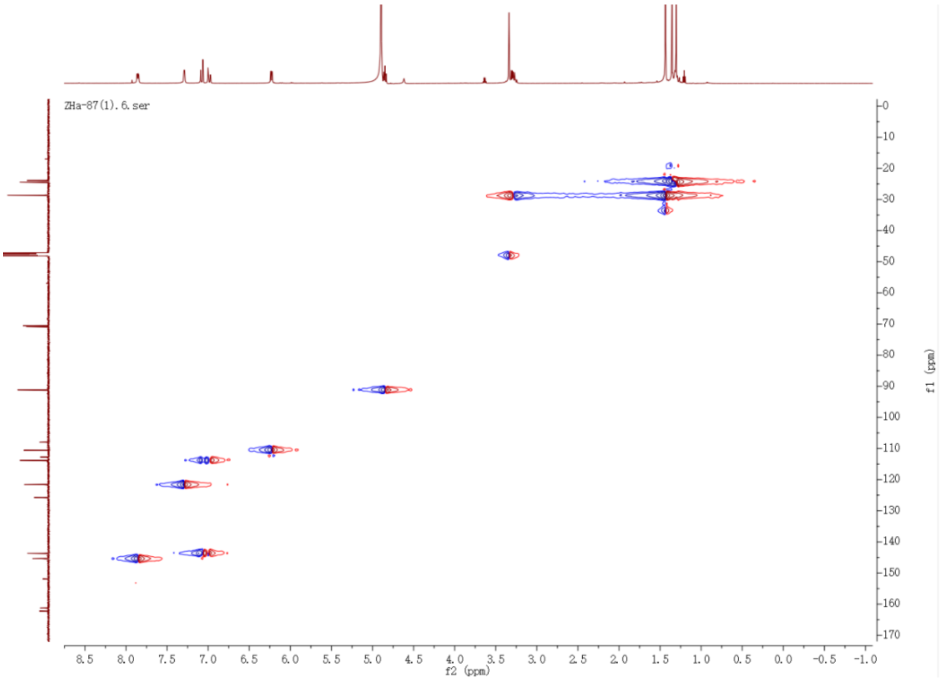

Figure S69. HSQC spectrum of **8**.

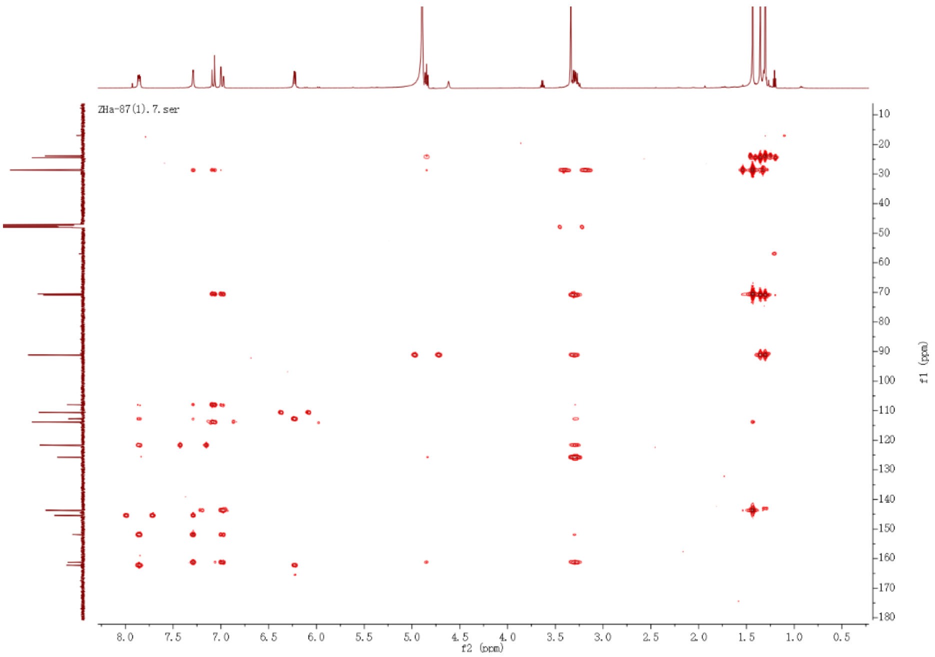

Figure S70. HMBC spectrum of **8**.

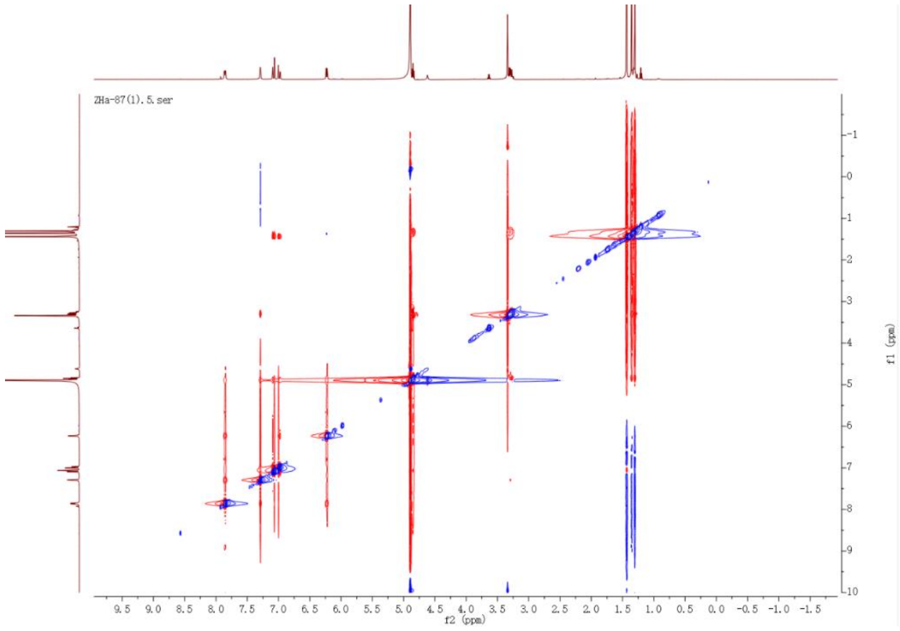

Figure S71. ROESY spectrum of **8**.

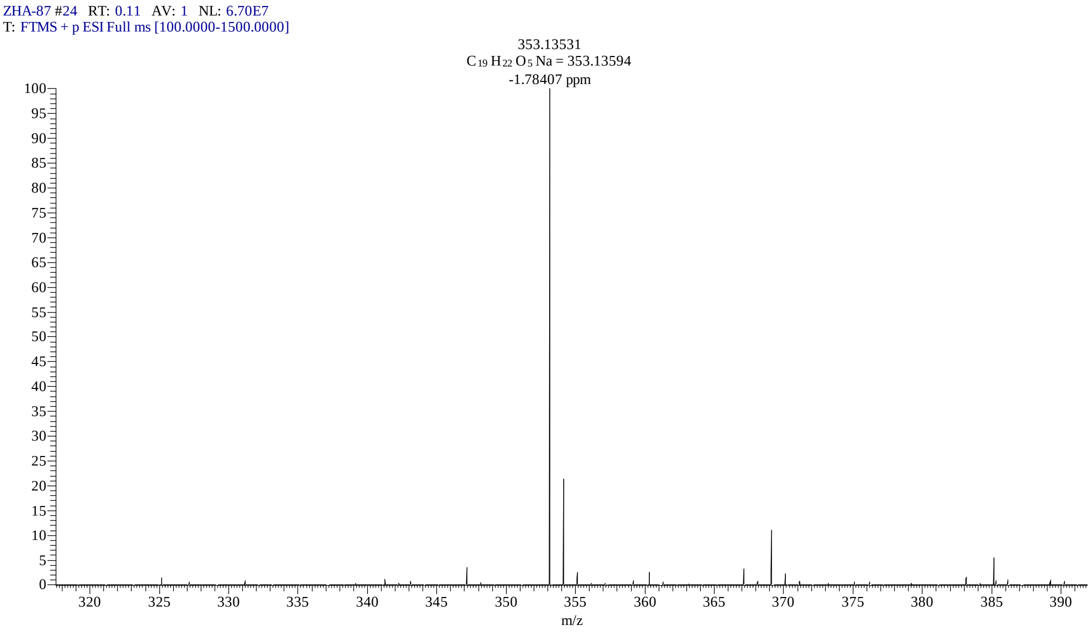

Figure S72. HR-ESI-MS spectrum of **8**.

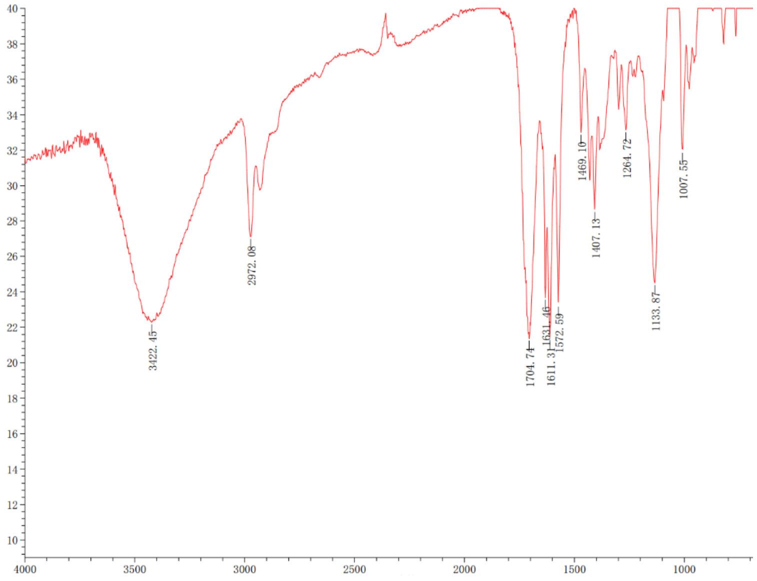

Figure S73. IR spectrum of **8**.

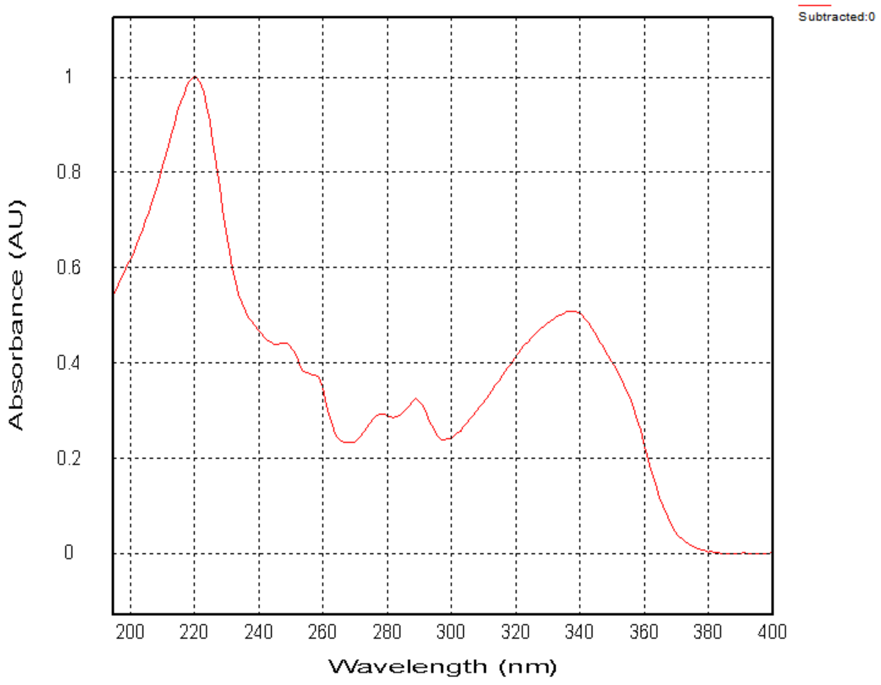

Figure S74. UV spectrum of **8**.

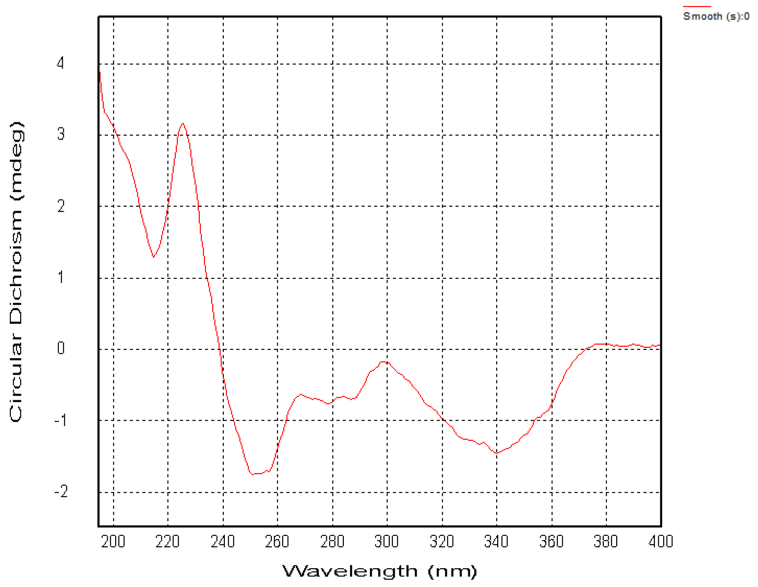

Figure S75. CD spectrum of **8**.

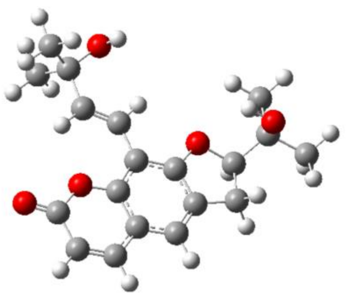

Figure S76. B3LYP/6-311+g (d) optimized lowest energy 3D conformers of **8**.

Table S1. Calculated ECD spectrum of 7

Standard orientation:

Center Number	Atomic Number	Atomic Type	Coordinates (Angstroms) X	Y	Z
1	6	0	-0.021510	-2.012483	0.005674
2	6	0	0.028748	-0.689626	-0.465411
3	6	0	-1.085011	0.135755	-0.610904
4	6	0	-2.304669	-0.456564	-0.240959
5	6	0	-2.409742	-1.782316	0.236438
6	6	0	-1.236649	-2.561744	0.359582
7	8	0	-3.421296	0.324268	-0.369935
8	6	0	-4.712561	-0.112966	-0.057617
9	6	0	-4.819627	-1.477278	0.432526
10	6	0	-3.721697	-2.262830	0.569970
11	8	0	-5.621738	0.672833	-0.217782
12	6	0	-0.988471	1.562751	-1.112479
13	6	0	-0.923282	2.568397	0.017259
14	6	0	0.030819	3.482227	0.262711
15	6	0	-0.093091	4.426215	1.436806
16	6	0	1.274886	3.684428	-0.568815
17	6	0	1.383986	-2.563543	0.039641
18	6	0	2.203490	-1.419166	-0.612599
19	8	0	1.293679	-0.286875	-0.775041
20	6	0	3.448330	-0.950976	0.166075
21	6	0	4.211515	0.090139	-0.670949
22	8	0	5.481235	0.281461	-0.032646
23	6	0	3.116664	-0.394106	1.556669
24	8	0	4.252148	-2.133032	0.269820
25	1	0	-1.310433	-3.580954	0.729876
26	1	0	-5.818486	-1.817469	0.676751
27	1	0	-3.821708	-3.281197	0.938622
28	1	0	-0.118561	1.643598	-1.767204
29	1	0	-1.876451	1.774459	-1.719896
30	1	0	-1.778000	2.536007	0.692243
31	1	0	-1.010385	4.251229	2.005957
32	1	0	0.759975	4.318253	2.120490
33	1	0	-0.094563	5.472156	1.101499
34	1	0	2.171139	3.622239	0.062934
35	1	0	1.380772	2.956393	-1.375132
36	1	0	1.279637	4.687664	-1.015952
37	1	0	1.492337	-3.491391	-0.528794
38	1	0	1.721373	-2.773204	1.059367

39	1	0	2.522960	-1.708828	-1.619915
40	1	0	4.352230	-0.298505	-1.688402
41	1	0	3.648001	1.029181	-0.716548
42	1	0	6.056179	0.802523	-0.605477
43	1	0	2.671265	-1.167678	2.187266
44	1	0	4.033058	-0.047159	2.041539
45	1	0	2.421500	0.447431	1.485179
46	1	0	5.153171	-1.841871	0.478546

Table S2. Calculated ECD spectrum of 8

Standard orientation:

Center Number	Atomic Number	Atomic Type	Coordinates (Angstroms) X	Y	Z
1	6	0	1.510643	-1.795357	-0.227182
2	6	0	0.944992	-0.508368	-0.201329
3	6	0	-0.426837	-0.233474	-0.100179
4	6	0	-1.237282	-1.394180	-0.021530
5	6	0	-0.710154	-2.705753	-0.055918
6	6	0	0.688241	-2.897528	-0.157900
7	8	0	-2.580610	-1.206491	0.081839
8	6	0	-3.526425	-2.245217	0.155418
9	6	0	-2.981607	-3.587147	0.107694
10	6	0	-1.642865	-3.793608	0.009846
11	8	0	-4.691127	-1.922673	0.244606
12	6	0	-0.883452	1.159265	-0.117017
13	6	0	-2.129460	1.643200	0.036252
14	6	0	-2.479502	3.118878	-0.027924
15	6	0	-3.200519	3.424360	-1.352527
16	6	0	-3.370268	3.492354	1.171989
17	6	0	3.010661	-1.645421	-0.282140
18	6	0	3.176415	-0.145113	-0.609854
19	8	0	1.885631	0.474083	-0.278899
20	6	0	4.274251	0.610644	0.166162
21	6	0	4.286196	2.094739	-0.231523
22	6	0	5.639863	-0.031404	-0.097170
23	8	0	4.038697	0.476191	1.573697
24	8	0	-1.310375	3.953721	-0.037972
25	1	0	1.083888	-3.909708	-0.173226
26	1	0	-3.695556	-4.400009	0.156797
27	1	0	-1.253836	-4.809059	-0.021322
28	1	0	-0.086492	1.875542	-0.296057
29	1	0	-2.971200	0.978018	0.195190

30	1	0	-3.501334	4.476336	-1.377698
31	1	0	-2.529829	3.227570	-2.193143
32	1	0	-4.091213	2.798444	-1.461476
33	1	0	-3.636630	4.552389	1.121141
34	1	0	-2.847380	3.306647	2.117736
35	1	0	-4.290339	2.898661	1.178787
36	1	0	3.483241	-2.280973	-1.036465
37	1	0	3.460602	-1.875476	0.691173
38	1	0	3.321013	0.007892	-1.687036
39	1	0	5.051679	2.620073	0.346388
40	1	0	3.316792	2.563362	-0.039334
41	1	0	4.512365	2.214389	-1.296908
42	1	0	5.661177	-1.068203	0.248453
43	1	0	6.410595	0.522274	0.445842
44	1	0	5.885206	-0.008146	-1.164061
45	1	0	3.183719	0.885149	1.773812
46	1	0	-0.861543	3.860956	0.814234

Table S3. 675 tagets of isolated componds.

NO.	Target	NO.	Target	NO.	Target	NO.	Target
1	HSD17B3	170	CXCL8	339	ADH1C	508	HIPK4
2	CA12	171	CSNK2A1	340	F13A1	509	ERN1
3	CA9	172	ACE2	341	CHRM4	510	PRPF4
4	GSK3B	173	IDE	342	CHRM2	511	PIK3C2G
5	ADORA1	174	TTR	343	PDE9A	512	MAP3K13
6	RGS4	175	CXCR1	344	HRH2	513	ICK
7	CHRM3	176	FABP1	345	TDP2	514	MAST1
8	RPS6KB1	177	BCAT2	346	MAPK9	515	HUNK
9	AURKA	178	NOTUM	347	ACHE	516	PRPF4B
10	GRM5	179	PTPRC	348	BACE1	517	DSTYK
11	TNKS2	180	ITGAV	349	CBR1	518	YES1
12	HSD11B1	181	HSPA1A	350	ALOX5	519	MAP2K3
13	GABRA5	182	GRK2	351	AURKB	520	PRKAA2
14	CCNB1	183	MKNK2	352	AKR1B1	521	MAP2K6
15	FNTB	184	MKNK1	353	FLT4	522	BLK
16	CCNB2	185	MMP12	354	PLK4	523	MAP3K20
17	GABRG2	186	GRK6	355	EPHB4	524	TGFBR2
18	CDK1	187	APEX1	356	NUAK1	525	ERBB2
19	P2RX7	188	SORT1	357	SQLE	526	MAP3K5
20	GRM1	189	KDM6B	358	MAOA	527	KCNK3
21	KCNA5	190	KDM4C	359	AKT1	528	PRCP

22	PDE10A	191	ABCC1	360	XDH	529	CFD
23	CA2	192	HCAR2	361	AKR1C3	530	ABCB1
24	CA5A	193	PAM	362	ESR2	531	PKM
25	MET	194	MMP13	363	NFKB1	532	GRIN2A
26	SLC8B1	195	MMP9	364	PLK1	533	CCNT1
27	MST1R	196	MMP2	365	TEK	534	RHOA
28	FNTA	197	MAP2K1	366	HDAC3	535	AXL
29	CYP19A1	198	CASP1	367	ADRA2C	536	TYRO3
30	NQO1	199	AKR1A1	368	HDAC2	537	OPRM1
31	NQO2	200	MTNR1A	369	ADRA2B	538	SLC6A2
32	XPO1	201	CTSA	370	ADRA1B	539	TACR2
33	KIT	202	KDM4E	371	HDAC1	540	SMYD2
34	FLT3	203	KDM4A	372	CSNK1D	541	MAP2K4
35	INSR	204	KDM4D	373	PTGER1	542	MAPK13
36	RET	205	SLCO1B1	374	MTOR	543	MAP2K7
37	CCNB3	206	SRD5A2	375	PIK3CG	544	F2R
38	FGFR3	207	KDM5C	376	PRKDC	545	MAPK12
39	KDR	208	HAO2	377	HSF1	546	AMPD3
40	NTRK1	209	PARP15	378	GYS1	547	CCR9
41	JAK2	210	PARP10	379	BRS3	548	GRIN1
42	LYN	211	MMP8	380	TGFBR1	549	CTSD
43	FGFR4	212	SLC16A3	381	PTK2B	550	HSD17B2
44	ALK	213	CXCR2	382	CLK4	551	PER2
45	FGR	214	FOLH1	383	GSK3A	552	POLB
46	NTRK2	215	GSTA1	384	PDE8B	553	TOP1
47	TIE1	216	GSTP1	385	PGR	554	TRPV4
48	ROS1	217	PYGL	386	MAPKAPK2	555	GCGR
49	NTRK3	218	PYGM	387	CSNK1G1	556	CETP
50	ADORA2A	219	PTPsigma	388	ADRA1A	557	KLK5
51	QPCT	220	MPI	389	PPOX	558	MMP1
52	TNKS	221	PHOSPHO1	390	PTGER4	559	OPRL1
53	THRB	222	DHODH	391	PTGER2	560	KCNE1
54	CYP17A1	223	CAPN1	392	PTGER3	561	GPR88
55	CTSC	224	PLEC	393	HCRTR2	562	CX3CR1
56	PDE2A	225	PPARD	394	HCRTR1	563	PDE1B
57	CTSK	226	GPR35	395	BCHE	564	SMO
58	CTSS	227	DDO	396	PTGS1	565	PRMT3
59	CTSL	228	CISD1	397	PTGS2	566	P2RX3
60	MAPK11	229	AKR1C1	398	CNR2	567	KCNQ1
61	CTSB	230	JAK3	399	PTAFR	568	PTP4A3
62	PABPC1	231	PDPK1	400	HRH3	569	IKBKE
63	PIM1	232	MAPK14	401	ADORA3	570	BTK
64	EPHX2	233	CHEK2	402	MB	571	KCNK9
65	PIM2	234	DUSP3	403	RNASEH1	572	DYRK2

66	PIM3	235	REN	404	GSR	573	SIRT3
67	CYP11B1	236	CDK9	405	ESR1	574	PLA2G2A
68	CYP11B2	237	LTA4H	406	QDPR	575	PTK6
69	GRM4	238	SRD5A1	407	CCNA1	576	LIPG
70	HMOX1	239	FABP4	408	SNCA	577	CHUK
71	HTR6	240	HSP90AA1	409	METAP2	578	HTR7
72	CSF1R	241	HAO1	410	ALDH5A1	579	VHL
73	ABL1	242	GRIK1	411	ABAT	580	CCKBR
74	FLT1	243	KMO	412	ICMT	581	KIF11
75	PDGFRB	244	MAP3K8	413	TRPV1	582	FKBP5
76	IGF1R	245	SLC16A1	414	MDM2	583	MMP14
77	DHFR	246	CCNC	415	MYLK	584	MMP7
78	GABRB3	247	CDK8	416	PSEN2	585	ADAM10
79	HTR2C	248	DAPK3	417	NPY5R	586	ABCC9
80	LCK	249	RAF1	418	PFKFB3	587	TUBB1
81	SYK	250	ALDH2	419	NPBWR1	588	CDK7
82	SRC	251	ALDH1A1	420	CACNA2D1	589	TACR1
83	ZAP70	252	ALDH3A1	421	VCP	590	CENPE
84	BMX	253	CD38	422	HTR5A	591	DRD1
85	IDO1	254	PSMB1	423	CACNA1B	592	CCNH
86	BRAF	255	NAAA	424	LRRK2	593	LTB4R
87	MERTK	256	CRHR1	425	AVPR2	594	SLC6A7
88	AR	257	C1R	426	AVPR1A	595	LIMK2
89	CASP3	258	CBFB	427	ITK	596	ADA
90	CASP7	259	DCTPP1	428	RGS8	597	COMT
91	ADRA2A	260	PGGT1B	429	ALOX5AP	598	HDAC6
92	DNMT3A	261	ABCG2	430	MAPKAPK5	599	TNK2
93	MAPK10	262	CHRNA5	431	MAPKAPK3	600	PPARG
94	CDK5	263	CHRNB2	432	IRAK4	601	SORD
95	PDE5A	264	CHRNA4	433	ACACB	602	TTL
96	SLC6A3	265	FGFR1	434	DYRK1B	603	PDE3B
97	PDE3A	266	EGLN3	435	BRD4	604	IL6ST
98	PDE4A	267	ALPL	436	ADAMTS5	605	F9
99	PDE11A	268	PTK2	437	ADAMTS4	606	RELA
100	PDE7A	269	TLR4	438	ROCK2	607	KCNMA1
101	PDE7B	270	KIF20A	439	KCNJ5	608	CASR
102	CNR1	271	MAPK8	440	KCNJ3	609	RIPK2
103	ADORA2B	272	PIK3R1	441	PSENEN	610	EGLN1
104	GPBAR1	273	GABRB2	442	APH1A	611	LDHA
105	CCNA2	274	TSPO	443	APH1B	612	VEGFA
106	CLK2	275	CCR1	444	NCSTN	613	CTSV
107	PARP1	276	CCR4	445	PSEN1	614	TYMS
108	CCNE2	277	CCR2	446	PDE4D	615	CASP6
109	CHEK1	278	PIK3CD	447	TYMP	616	AURKC

110	CCND1	279	CDC7	448	IKBKB	617	ABL2
111	MAP3K11	280	ELANE	449	HLCS	618	ECE2
112	CDK2	281	HSP90AB1	450	SIRT2	619	NAT1
113	CDK4	282	CTRB1	451	ICAM1	620	ALPG
114	CCNE1	283	TNNI3K	452	ACE	621	PLAA
115	MAP3K9	284	LIPE	453	SELE	622	ITGB2
116	PRKCB	285	IDH1	454	TERT	623	ITGAL
117	GLI2	286	CHRM5	455	FABP3	624	IMPDH1
118	GLI1	287	GAK	456	PLA2G10	625	SLC5A2
119	CDK5R1	288	BACE2	457	TRPA1	626	PAK1
120	CYP1A2	289	TLR9	458	PRKCG	627	DPP4
121	CA1	290	PLAU	459	SLC6A4	628	CDK6
122	DYRK1A	291	MC4R	460	ADH1A	629	CDK3
123	CA14	292	FAAH	461	CYP2C19	630	UPP1
124	TOP2A	293	F2	462	PSMB5	631	HTR1A
125	MAOB	294	PRSS1	463	PKN2	632	APP
126	CA7	295	ADRA1D	464	CDC42	633	TK1
127	CA6	296	PRKCA	465	ADH7	634	STK38
128	CA4	297	KCNN1	466	EDNRA	635	TAOK2
129	CA13	298	ROCK1	467	VCAM1	636	OXSR1
130	GABRA3	299	MBD2	468	SIGMAR1	637	MAK
131	GABRA1	300	P2RY1	469	PRKD1	638	STK39
132	MIF	301	ALOX15	470	NR4A1	639	CDKL5
133	CLK1	302	PRKCD	471	DBF4	640	CDKL3
134	DAO	303	PRKCE	472	AHR	641	MAP3K6
135	CES1	304	PRKCH	473	BRD3	642	MAP3K15
136	CREBBP	305	PRKCQ	474	CACNA1I	643	BDKRB1
137	MCHR1	306	MAPK1	475	RORC	644	SCD
138	EPHB3	307	HMGCR	476	BRPF1	645	MAP3K14
139	PARP2	308	DRD2	477	SLC27A1	646	MAPK3
140	GABRA2	309	DRD3	478	PDGFRA	647	HTR2B
141	EGFR	310	NR3C1	479	CSK	648	HTR1D
142	HTT	311	CSNK1E	480	HCK	649	HDAC4
143	CA3	312	CHRNA3	481	FGFR2	650	CCR5
144	CA5B	313	CHRNB4	482	PANK3	651	RPS6KA2
145	BDKRB2	314	TKT	483	GPR139	652	TDP1
146	TAAR1	315	CDC25A	484	BAD	653	SLC28A2
147	WEE1	316	PTPRF	485	S1PR1	654	SLC29A1
148	MGLL	317	NR3C2	486	DUT	655	ADK
149	EIF2AK2	318	SCN9A	487	IMPDH2	656	IGFBP3
150	USP10	319	SERPINA6	488	FPR1	657	PRKACA
151	USP13	320	PDE4B	489	SHH	658	YARS
152	NOS1	321	NUDT1	490	FKBP1A	659	ST6GAL1
153	NOS2	322	ILK	491	CAMK2D	660	GBA

154	NOS3	323	MMP3	492	UTS2R	661	MANBA
155	JUN	324	OPRK1	493	CHRNA7	662	HSPA8
156	GRM2	325	AHCY	494	OPRD1	663	GRK1
157	TBXAS1	326	KCNA3	495	KCNH2	664	GAPDH
158	FADS1	327	TYK2	496	F10	665	OGA
159	PTPN1	328	CHRM1	497	BRD2	666	HSPA5
160	CYP2A6	329	MCL1	498	PLAT	667	IL2
161	CDC25B	330	CCND2	499	PLA2G7	668	HK2
162	CES2	331	TGM2	500	GRIA2	669	HK1
163	MPO	332	JAK1	501	TACR3	670	GAA
164	GCK	333	CCND3	502	SCN2A	671	PNP
165	CTSH	334	PARP3	503	SCN10A	672	TNF
166	CTSF	335	BRD9	504	PTGFR	673	LGALS3
167	PIK3CB	336	MTNR1B	505	ATM	674	LGALS9
168	PIK3CA	337	EPHX1	506	OXTR	675	ADAM17
169	ADH1B	338	MALT1	507	GRK7		

Table S4. 3162 targets of RA

NO.	Targets of RA	NO.	Targets of RA	NO.	Targets of RA	NO.	Targets of RA
1	PTPN22	792	TSPAN33	1583	MAP3K3	2374	MK280144-285
2	CIITA	793	ANPEP	1584	ETFA	2375	MK280144-354
3	IL6	794	HSPA8	1585	ITGB7	2376	MN308685
4	IL10	795	TNFRSF8	1586	HPSE	2377	NONHSAG001753.2
5	HLA-DRB1	796	HAS2	1587	HSD11B2	2378	RF00017-4933
6	TNF	797	BST2	1588	LCN2	2379	RF00017-6504
7	IRF5	798	ETS2	1589	SDC4	2380	RF00026-969
8	SLC22A4	799	MAP2K4	1590	SEMA4A	2381	lnc-CEP76-3
9	CD244	800	BCL2L11	1591	COL11A2	2382	lnc-FAM109A-1
10	NFKBIL1	801	FADS2	1592	GAS6	2383	lnc-FAM133B-2
11	PADI4	802	INPP5B	1593	GYPC	2384	lnc-FBXO43-3
12	MIF	803	OS9	1594	SERPINB2	2385	lnc-GATA3-19-001
13	MIR146A	804	HCP5	1595	SFRP1	2386	lnc-GATA3-19-002
14	AP4B1-AS1	805	SOD2-OT1	1596	SPHK2	2387	lnc-GATA3-20
15	MIR155	806	PRF1	1597	NOD1	2388	lnc-IGSF3-3
16	STAT4	807	CD1A	1598	QKI	2389	lnc-PSMB9-14
17	TLR1	808	YY1	1599	COPS5	2390	lnc-PSMD5-5
18	ZFAS1	809	IDO1	1600	HLA-E	2391	lnc-RAB5B-2
19	H19	810	IL24	1601	HPX	2392	lnc-RPLP1-6
20	MIR132	811	ENG	1602	PTN	2393	lnc-TLE3-16
21	SNHG29	812	MIR320A	1603	VTCN1	2394	piR-30934
22	SUPT20H	813	PGR-AS1	1604	GRP	2395	piR-31508-004
23	HOTAIR	814	MAP2K6	1605	PRELP	2396	piR-32810-131
24	CHRNG	815	RPS6KA4	1606	TFF2	2397	piR-34839
25	IL2RA	816	CASP7	1607	HOXD9	2398	piR-36182-002

26	MALAT1	817	SLAMF1	1608	MRPL28	2399	piR-36455
27	UCA1	818	GIN1	1609	TNFSF9	2400	piR-37170-024
28	MIR150	819	KCP	1610	IL9R	2401	piR-38259
29	CRP	820	HSALNG0049424	1611	MDGA2	2402	piR-38580-086
30	SNHG28	821	TAC3	1612	TNFSF8	2403	piR-39892
31	IL1B	822	NFE2L2	1613	KIR2DL1	2404	piR-48007
32	CTLA4	823	A2M	1614	ERV3-1	2405	piR-48156
33	FBXL19-AS1	824	TRAF2	1615	DELEC1	2406	piR-50346
34	IL17A	825	TXNDC5	1616	MIR574	2407	piR-51327
35	FCRL3	826	GATA4	1617	MIRLET7B	2408	piR-51449
36	TRAF1	827	HSP90AB1	1618	MIR199A1	2409	piR-51518
37	HLA-DQB1	828	LOC128462409	1619	MIR199B	2410	piR-55194-146
38	CCR6	829	FLT1	1620	MIRLET7G	2411	piR-56480-015
39	CD40	830	ERBB3	1621	MIR29C	2412	piR-61240-151
40	IL1RN	831	BATF	1622	SNHG14	2413	CM034955-135
41	TNFRSF1B	832	JDP2	1623	MIR15A	2414	CM034955-136
42	IL2	833	PHTF1	1624	MIR188	2415	HE856162
43	MACIR	834	SMIM20	1625	MIR551B	2416	HSALNG0049239
44	IL18	835	LGALS1	1626	MIR652	2417	HSALNG0049451
45	MMP3	836	TNFRSF12A	1627	MIR496	2418	HSALNG0086584
46	IL6ST	837	IL12RB1	1628	LINC01736	2419	HSALNG0086587
47	IL1A	838	TBX21	1629	LOC101927636	2420	HSALNG0140824
48	MTHFR	839	AICDA	1630	MIR1184-1	2421	HSALNG0143084
49	IL2RB	840	CCL7	1631	MIR6089	2422	LOC124903019
50	TNFRSF1A	841	CD70	1632	ITPR3	2423	LOC124903756
51	IFNG	842	GHRL	1633	TLR6	2424	RF00017-8932
52	TNFSF11	843	RPS12P4	1634	PWAR1	2425	lnc-HLA-DOB-1
53	FCGR3A	844	C3	1635	RAF1	2426	lnc-LHFPL6-7
54	IL4	845	IFNA1	1636	NOTCH1	2427	piR-32214-583
55	SAA1	846	TERT	1637	TGFBR2	2428	piR-39341-038
56	COMP	847	PRKDC	1638	PLK1	2429	piR-43583-375
57	IL1R1	848	AOX1	1639	HDAC3	2430	piR-48222-181
58	ACP5	849	RO60	1640	ALDOA	2431	piR-55072-002
59	HLA-B	850	ARAP1	1641	UBA1	2432	HSALNG0049404-002
60	MMP1	851	REL-DT	1642	CDK1	2433	piR-35002-057
61	CCL2	852	ASPN	1643	SIRT3	2434	LRRC56
62	MIF-AS1	853	HDAC1	1644	CASP4	2435	IGK
63	IL6-AS1	854	AHSG	1645	CYP11B2	2436	CCND1
64	LOC126859963	855	RORC	1646	EFNB1	2437	NTRK2
65	SERPINH1	856	ELAVL1	1647	GNB3	2438	TGFBR1
66	PTGS2	857	BANK1	1648	MDM4	2439	RPS6KA3
67	LTA	858	FCRLA	1649	NTN1	2440	EP300
68	BLK	859	SPTAN1	1650	EIF2S3	2441	MERTK

69	HLA-DQA1	860	TRPV1	1651	NRP2	2442	NOTCH2
70	CCR5	861	QPCT	1652	APOC3	2443	DYRK1A
71	CXCL8	862	PFKFB3	1653	DLL1	2444	EGLN1
72	IL1RAPL2	863	PLCL1	1654	EFNA5	2445	EIF2AK3
73	MMP13	864	TXNDC11	1655	HSPB8	2446	FZD4
74	TNFRSF11B	865	NRM	1656	MUTYH	2447	ITGA6
75	SLC11A1	866	LOC100506023	1657	SIRT6	2448	PKM
76	FCGR2A	867	CFTR	1658	DAXX	2449	RARB
77	IL15	868	ITGAX	1659	FOXM1	2450	CAV1
78	PHF19	869	DNASE1	1660	HIPK2	2451	CYBB
79	REL	870	THPO	1661	MAPKAP1	2452	MYB
80	IL6R	871	CCL22	1662	PSMA4	2453	ODC1
81	PADI2	872	MYD88	1663	S100A11	2454	REN
82	TLR4	873	BRAF	1664	SEMA7A	2455	SMPD1
83	VCAM1	874	MDM2	1665	SNAI2	2456	COL4A1
84	IL21	875	ADM	1666	SOCS2	2457	EIF4G1
85	ICAM1	876	CCL3L1	1667	ADGRG1	2458	F5
86	VEGFA	877	KIR2DL2	1668	BMP5	2459	FURIN
87	S100A9	878	IFNA2	1669	IL36RN	2460	KAT2B
88	TTR	879	NTF3	1670	ITGA9	2461	MRE11
89	FCGR3B	880	IRAK4	1671	MTNR1A	2462	SLC2A3
90	HMGB1	881	CHRM3	1672	NEDD9	2463	C1R
91	FLG	882	FLNC	1673	SEMA3F	2464	DDIT3
92	CD40LG	883	ATP6V1F	1674	APOA4	2465	HK2
93	NLRP1	884	FLNC-AS1	1675	CLEC3B	2466	HUWE1
94	IL7	885	TPI1P2	1676	EXOC5	2467	IHH
95	BGLAP	886	SIMALR	1677	IL13RA1	2468	ITGB5
96	FSTL1	887	ENSG00000271553	1678	IRAK2	2469	KLKB1
97	FOXP3	888	ENSG00000242162	1679	MYO3A	2470	KRT8
98	IL23R	889	ENSG00000224163	1680	PGLYRP1	2471	LIFR
99	CSF2	890	lnc-IRF5-3	1681	PIAS3	2472	LOXL2
100	ITGAV	891	HSALNG0061260	1682	AGXT2	2473	LTA4H
101	SLC19A1	892	OPRM1	1683	CD109	2474	PRMT5
102	MBL2	893	CSN1S1	1684	CEBPD	2475	CD34
103	HLA-DMA	894	LDLR	1685	CMKLR1	2476	METAP2
104	TAGAP	895	PLA2G4A	1686	CRYGD	2477	OSMR
105	MMP9	896	HDAC4	1687	EXOC4	2478	EFEMP1
106	CCR2	897	MALT1	1688	RNASE2	2479	INPP5D
107	MAFB	898	RFC1	1689	SIGIRR	2480	KPNA2
108	TIMP1	899	TLR5	1690	SLC6A11	2481	ORAI1
109	CD28	900	CD209	1691	SOCS6	2482	PSMB1
110	TNFSF13B	901	MIR22HG	1692	TNFAIP1	2483	SFTPA1
111	HLA-DPB1	902	MIR27A	1693	TNIP2	2484	SOX5
112	TLR2	903	MIR221	1694	UNC5B	2485	USP5

113	S100A8	904	SNORD15A	1695	CERS6	2486	AGTR2
114	CCL5	905	ADAM15	1696	GSTM4	2487	CD96
115	CSF1	906	ANGPTL4	1697	LMO4	2488	CNDP2
116	HP	907	CCL8	1698	PPP1R9A	2489	CNP
117	FAS	908	MIR23A	1699	TLR10	2490	DRD3
118	OLAH	909	TPO	1700	CD99	2491	FSCN1
119	DDX39B	910	PAX5	1701	CEACAM8	2492	LY96
120	ATIC	911	MITF	1702	FGF21	2493	NLRP12
121	NR4A2	912	IGHE	1703	IL36G	2494	PDIA3
122	PDCD1	913	NCF2	1704	MTREX	2495	ROBO3
123	ADIPOQ	914	LSP1	1705	NHLH2	2496	SFTPB
124	PTPRC	915	GDF5	1706	NKIRAS2	2497	SLC7A5
125	TNFAIP3	916	LPA	1707	RIT2	2498	TRIM32
126	MMP2	917	MIR5196	1708	ANGPTL2	2499	AFF2
127	TNFRSF13C	918	ENSG00000274029	1709	CCL16	2500	CD63
128	TNFRSF11A	919	GPANK1	1710	CCL24	2501	CLCF1
129	MICB	920	TIMP3	1711	CEP72	2502	GDF11
130	SELE	921	RAC1	1712	FOXJ1	2503	IVNS1ABP
131	IL13	922	TTN	1713	GBP6	2504	S100A6
132	HLA-DMB	923	IFNGR1	1714	IRX1	2505	TREX1
133	TNFAIP6	924	IGF2	1715	LVRN	2506	ACKR1
134	MAPK14	925	TXN	1716	N4BP1	2507	BCL3
135	RELB	926	PADI3	1717	SPSB1	2508	KHDRBS1
136	S100A12	927	COX5A	1718	YTHDF2	2509	KLF10
137	CCL3	928	THBS1	1719	AHRR	2510	NPPC
138	IL37	929	ICA1	1720	CLEC4E	2511	PLA2G2D
139	ADAM17	930	KLRD1	1721	ENOX1	2512	SFTPC
140	PPP1CA	931	IL1F10	1722	IFNK	2513	SPRY1
141	CXCL10	932	IGHM	1723	RCHY1	2514	STAB1
142	TNIP1	933	MIR211	1724	SPA17	2515	ASIC3
143	CHI3L1	934	TRPV4	1725	SPAG16	2516	ASRGL1
144	NOS2	935	HSPA1L	1726	WFDC1	2517	FLT3LG
145	BTNL2	936	PFKL	1727	ARAP2	2518	G3BP1
146	SOCS1	937	ARHGEF3	1728	CST5	2519	IKZF2
147	PRTN3	938	CCN4	1729	FNDC5	2520	LPXN
148	SYK	939	TRHDE	1730	GALNT18	2521	LSM4
149	FCGR2B	940	ZNF175	1731	CD24	2522	MAST3
150	IL17RA	941	PHACTR3	1732	STATH	2523	MKKS
151	CD4	942	FNDC1	1733	KIR2DS4	2524	SERPINB1
152	SIAE	943	ZNF311	1734	KIR2DS1	2525	TSC22D3
153	SPP1	944	LINC00158	1735	KIR2DS3	2526	UHRF1
154	CARD8	945	C5AR1	1736	KIR3DS1	2527	DSPP
155	NR3C1	946	IL20RB	1737	TNXA	2528	FUT1
156	CXCR5	947	AGPAT1	1738	LY6G6E	2529	MRC1

157	DHODH	948	ATF6B	1739	MIRLET7C	2530	NFIL3
158	MMP12	949	LINC00593	1740	LINC00304	2531	NLRC5
159	RETN	950	PCAT29	1741	MIR141	2532	AFF1
160	PPP1CB	951	HSALNG0049427	1742	MIR203A	2533	C1D
161	HSPD1	952	ATP1A1	1743	MIR181A2	2534	CD53
162	PPP1CC	953	IL10RB	1744	MIR375	2535	CPQ
163	BST1	954	EOMES	1745	MIR9-1	2536	KIR3DL1
164	CD79A	955	SERPINC1	1746	SNORD3A	2537	MT3
165	TRAF6	956	ATF2	1747	MIR137	2538	PMAIP1
166	NFKBIA	957	CTSL	1748	MIR31HG	2539	SCG2
167	GPI	958	MIR145	1749	MIR589	2540	CHI3L2
168	JUN	959	MIR26B	1750	HAGLR	2541	ORM2
169	MAPK8	960	MAP2K3	1751	IGH	2542	OXA1L
170	IL23A	961	F2	1752	MIR323A	2543	POU2AF1
171	ITGB2	962	MMP15	1753	MIR671	2544	SNAPIN
172	MAP3K8	963	EGR1	1754	LINC02899	2545	STXBP6
173	PRRC2A	964	TLR8	1755	MIR382	2546	CHST4
174	CD44	965	CSK	1756	MIR573	2547	CKLF
175	CCR3	966	GUSB	1757	MIR769	2548	DEFA3
176	FASLG	967	SEMA4D	1758	LINC02099	2549	PDLIM2
177	CXCR3	968	CD59	1759	MIR300	2550	PPARGC1B
178	CCRL2	969	PTHLH	1760	MIR363	2551	SULT1A3
179	MEFV	970	KLRC1	1761	RNY3	2552	GPR15
180	CXCL12	971	MIR204	1762	SVIL-AS1	2553	TRIM3
181	NAMPT	972	MIR212	1763	TSPOAP1-AS1	2554	ZBTB10
182	HSPA4	973	DNMT1	1764	LINC01504	2555	DERL3
183	CD36	974	NCAM1	1765	OSER1-DT	2556	DLEU2
184	FOLR2	975	SEMA3A	1766	MIR4478	2557	FER1L4
185	PIK3CG	976	FCAR	1767	PGM5P2	2558	RMRP
186	LRRK2	977	EBI3	1768	BDP1P	2559	MIRLET7D
187	HSPA5	978	CD200R1	1769	TTF2	2560	LINC01554
188	MPO	979	PMEL	1770	BSN	2561	MIR192
189	TFRC	980	VAV1	1771	ATP6V1G2	2562	MIR210
190	CRH	981	RIPK1	1772	F2R	2563	SNHG5
191	MAPK1	982	BACH2	1773	COL10A1	2564	HOTAIRM1
192	STAT1	983	EZH2	1774	NKX2-1	2565	MIR20A
193	CCR1	984	MAPK9	1775	AMPH	2566	MIR302D
194	BTLA	985	CLU	1776	KRT7	2567	MIR331
195	CLEC16A	986	F13A1	1777	A2ML1	2568	CASC15
196	GZMB	987	CALCRL	1778	CD1B	2569	MIR19B1
197	COL2A1	988	BPI	1779	DSC1	2570	MIR29B2
198	FPGS	989	OSCAR	1780	CD1C	2571	MIR486-1
199	OSM	990	MIR124-1	1781	CD1E	2572	CHKB-CPT1B
200	CXCL1	991	MIR522	1782	PSMB11	2573	MIR20B

201	TLR3	992	CEP57	1783	EPPK1	2574	MIR421
202	IL16	993	STAG1	1784	PLA2G3	2575	MIR613
203	APOH	994	GRHL2	1785	FCRL6	2576	P2RX5-TAX1BP3
204	CX3CR1	995	PTTG1	1786	DSP	2577	RNY4
205	NPSR1	996	TPD52	1787	BAX	2578	TRBV11-2
206	CD69	997	SLAMF6	1788	CTSS	2579	MIR588
207	ELANE	998	ABHD6	1789	ADAMTSL1	2580	THRIL
208	CCL20	999	RPP14	1790	F2RL2	2581	EPS15-AS1
209	SERPINA3	1000	VPS37C	1791	THY1	2582	MIR1246
210	ITGAM	1001	ARL14	1792	ITCH	2583	MIR4701
211	AGER	1002	DGUOK-AS1	1793	TG	2584	TRL-TAG1-1
212	BSG	1003	LOC100506403	1794	PPIL2	2585	LOC102724971
213	CD5	1004	PDGFRB	1795	PSMG2	2586	PIK3R1
214	SAA4	1005	FGF1	1796	ZNF365	2587	FBL
215	IL11	1006	VEGFC	1797	NKX2-3	2588	CASP10
216	CXCR4	1007	CDKN3	1798	CTNNB1	2589	STAT2
217	PTGS1	1008	PTGER2	1799	GSK3B	2590	WEE1
218	CD80	1009	VEGFD	1800	SETD2	2591	CSF2RB
219	BMP6	1010	CCL17	1801	NR1H2	2592	EGR2
220	ICOSLG	1011	ROCR	1802	CD6	2593	S1PR2
221	LTF	1012	LINC01082	1803	MYDGF	2594	EIF4H
222	CCR7	1013	CBL	1804	PRLR	2595	CLIC1
223	MMP14	1014	IGFBP3	1805	AREG	2596	ANAPC4
224	IL32	1015	PGR	1806	SLC22A1	2597	FSTL4
225	LINC02605	1016	HNRNPA2B1	1807	SEMA5A	2598	EGFL8
226	CD86	1017	LPAR1	1808	ANXA4	2599	TMT1B
227	STEAP4	1018	MAP3K2	1809	CD200	2600	TSPAN32
228	TNFRSF6B	1019	FCN2	1810	SFRP5	2601	MCCD1
229	TLR9	1020	PTPRT	1811	CIP2A	2602	STAT5B
230	SH2D1A	1021	SOD3	1812	NREP	2603	XRCC5
231	IL22	1022	RNGTT	1813	MIR590	2604	SIGLEC5
232	SOCS3	1023	KLRB1	1814	MIR575	2605	NTRK3
233	PRSS2	1024	KIR2DL3	1815	LOC654780	2606	EPHB2
234	IFNB1	1025	LCE3C	1816	LINC02381	2607	CASR
235	TIMP2	1026	LCE3B	1817	ENSG00000259514	2608	JAG1
236	CX3CL1	1027	MIR222	1818	KMT2A	2609	SMO
237	GNAQ	1028	MIR181D	1819	POLH	2610	STK11
238	ZFP36	1029	DSG1	1820	COX4I1	2611	AGT
239	CSF1R	1030	GABARAPL3	1821	HCFC1	2612	BCHE
240	HAS1	1031	TIRAP	1822	AARS2	2613	CTH
241	LECT2	1032	FLT4	1823	DHX16	2614	KAT5
242	TNFRSF10A	1033	LAIR1	1824	HSPA6	2615	KCNQ1
243	TNFSF13	1034	HNF1A-AS1	1825	IQGAP1	2616	VCP
244	SAA2	1035	MIR381	1826	RCAN1	2617	DNM1L

245	TNFRSF9	1036	RNASET2	1827	TCF7	2618	MSH6
246	CAST	1037	TLE3	1828	VPS11	2619	NRXN1
247	CCL21	1038	FBXW2	1829	REV1	2620	PRMT1
248	LIF	1039	GPR31	1830	SLC22A7	2621	STK4
249	CTSB	1040	LINC02539	1831	CDC5L	2622	TNNI3
250	MMP8	1041	LINC02132	1832	SPAG1	2623	ACTA2
251	C5	1042	ENSG00000249141	1833	VTI1B	2624	ACTG1
252	ALOX5	1043	ENSG00000269918	1834	NLRX1	2625	BRD4
253	IL18R1	1044	ENSG00000272221	1835	XPO5	2626	CPT1A
254	ENO1	1045	ENSG00000272980	1836	CAPN11	2627	FECH
255	TRB	1046	CM021573-029	1837	CNTRL	2628	GPX4
256	IL10RA	1047	CM034956-344	1838	GTPBP2	2629	HSF1
257	CTSK	1048	TERC	1839	MYO1B	2630	NPHS1
258	CR1	1049	PCSK9	1840	SLC35B2	2631	NQO1
259	SYVN1	1050	TSPO	1841	ABCC10	2632	NR1D1
260	CCR4	1051	SCGB1A1	1842	RPS25	2633	PARK7
261	ZNF334	1052	SHBG	1843	CEP192	2634	VCL
262	CCL4	1053	MIR151A	1844	RSPH3	2635	WNT1
263	HTRA1	1054	MAPK13	1845	TMEM30B	2636	ABCC8
264	CILP	1055	NFKBIB	1846	YIPF3	2637	B4GALNT1
265	CTSG	1056	SHH	1847	HECTD4	2638	CA4
266	B2M	1057	FKBP5	1848	CCDC116	2639	CETP
267	ADORA3	1058	GHSR	1849	SUSD6	2640	CSTB
268	PLB1	1059	GDF15	1850	CCDC153	2641	CYSLTR2
269	ANXA5	1060	TIA1	1851	CENATAC	2642	FAAH
270	ITGAL	1061	E2F2	1852	TMEM179	2643	FGF23
271	FOS	1062	FCN1	1853	HSPA7	2644	GHR
272	IKBKB	1063	PDCD5	1854	C21orf140	2645	GSTM3
273	NFKB1	1064	APLN	1855	LINC00824	2646	KDM4B
274	JAK3	1065	MOB3B	1856	SPATA31F2P	2647	LDHB
275	PTPN2	1066	CASC2	1857	PSMB8-AS1	2648	MAP3K11
276	TNFSF14	1067	MIR125B1	1858	CENATAC-DT	2649	MC2R
277	CXCL9	1068	GRN	1859	LINC02621	2650	MC4R
278	FCGR1A	1069	BTK	1860	LINC02865	2651	MSTN
279	TGFB1	1070	FLNB	1861	ENSG00000255176	2652	NTF4
280	KLRK1	1071	LY6G5B	1862	ENSG00000258989	2653	PML
281	ANXA1	1072	KCNIP4	1863	NEFHP1	2654	PTPRS
282	CXCL5	1073	CFH	1864	RNU6-921P	2655	RIPK2
283	ITGA4	1074	CEBPB	1865	RNU6-959P	2656	SPTBN1
284	IL21R	1075	TCN2	1866	RPS2P29	2657	TALDO1
285	PLA2G2A	1076	PECAM1	1867	ENSG00000259598	2658	TCF3
286	CHUK	1077	LGALS8	1868	LOC124901197	2659	TGFBI
287	IL18BP	1078	TUG1	1869	RNY4P14	2660	THBS2
288	SELL	1079	DLAT	1870	GAPDHP64	2661	UNG

289	GAS5	1080	LALBA	1871	RNA5SP184	2662	YWHAQ
290	SRF	1081	CCL1	1872	RNU6-299P	2663	CRYAA
291	IL22RA1	1082	IL31	1873	ENSG00000271581	2664	CYP7B1
292	PLA2G10	1083	VPREB1	1874	ENSG00000280216	2665	PPM1A
293	JAM3	1084	HNRNPC	1875	H3P5	2666	SAT1
294	SIGLEC1	1085	MIR483	1876	lnc-FCGR2B-2	2667	SERPINA6
295	CDH11	1086	CASTOR1	1877	ENSG00000273112	2668	SERPINF1
296	GOLGB1	1087	RSBN1	1878	LOC124903162	2669	ST3GAL3
297	TNFRSF4	1088	IGF1R	1879	CM034952-180	2670	STUB1
298	DHFR	1089	SMAD3	1880	ENSG00000220614	2671	CARD9
299	IL26	1090	TNFRSF10B	1881	HSALNG0007924	2672	CEACAM1
300	CCN6	1091	MAPK3	1882	HSALNG0087578	2673	CLIP1
301	SFTPD	1092	ALPP	1883	HSALNG0094037	2674	DPEP1
302	ACAN	1093	RNU6-1	1884	HSALNG0135499	2675	DVL2
303	ADAMTS4	1094	HSPG2	1885	HSALNG0149400	2676	GNS
304	AIRE	1095	DSG3	1886	LOC124901449	2677	HABP2
305	IL1RAP	1096	SNRNP70	1887	RF00017-1278	2678	MKI67
306	TAP2	1097	PRKCA	1888	CM034965-071	2679	MMP20
307	ICOS	1098	APRT	1889	ENSG00000288975	2680	NONO
308	LTBR	1099	SDC1	1890	HSALNG0013961	2681	PLTP
309	DEK	1100	PGF	1891	HSALNG0021696	2682	REV3L
310	TNFRSF18	1101	GBA1	1892	HSALNG0049428	2683	ROBO1
311	FPR2	1102	CREB1	1893	HSALNG0063445	2684	SIRT5
312	STAT3	1103	ITGA5	1894	HSALNG0087579-001	2685	TRPS1
313	CSF3	1104	EPHB1	1895	HSALNG0087579-002	2686	ABCC5
314	IL17B	1105	PDE3A	1896	HSALNG0087581	2687	CASP5
315	ANKRD55	1106	MAP2K7	1897	HSALNG0101767	2688	CDA
316	PRG4	1107	SPHK1	1898	HSALNG0144237	2689	COL5A2
317	VDR	1108	BAK1	1899	RF00017-3303	2690	CPA6
318	C4A	1109	ENPP2	1900	RF00017-4668	2691	CRABP2
319	TP53	1110	ICAM2	1901	RF00017-5477	2692	CRHR1
320	MICA	1111	CD276	1902	lnc-HLA-DRB1-8	2693	DCK
321	HLA-DRA	1112	SLCO1C1	1903	lnc-SLC35G5-2	2694	EYA4
322	CAT	1113	PWAR4	1904	piR-52008-011	2695	GADD45A
323	ABCB1	1114	MST1	1905	piR-52916-031	2696	GDAP1
324	TPMT	1115	POU2F1	1906	HSALNG0001266	2697	GDF2
325	CD247	1116	TNFSF18	1907	HSALNG0001267	2698	HMMR
326	HAPLN1	1117	CEP170B	1908	LOC107986672	2699	IRF9
327	XIST	1118	GMCL2	1909	RF00019-027	2700	KAT7
328	NOD2	1119	ABHD16A	1910	VKORC1	2701	KMT2C
329	LACC1	1120	JAK1	1911	WNT5A	2702	KREMEN1
330	HLA-DQA2	1121	BCL2L1	1912	TPSAB1	2703	MBD4

331	IL3	1122	ZFP36L1	1913	HSPE1	2704	NCOR1
332	APOB	1123	PINX1	1914	CXCL6	2705	OPRK1
333	MUC5B	1124	RAVER1	1915	MIR340	2706	OPTN
334	NLRP3	1125	TMEM151B	1916	MIR497	2707	PDGFA
335	HLA-DRB4	1126	GATA3-AS1	1917	FGFR2	2708	PDGFC
336	PRL	1127	TAGAP-AS1	1918	PCNA	2709	PER2
337	TYMS	1128	LINC02934	1919	PLA2G6	2710	PON2
338	IL19	1129	LOC105374780	1920	HEXA	2711	PPAT
339	LINC01672	1130	ENSG00000246477	1921	NCF1	2712	PSMB7
340	PRKCQ	1131	HSALNG0074148-002	1922	TKT	2713	PTPN13
341	HMGB2	1132	lnc-C5-1	1923	GPX1	2714	PTPN3
342	IFNG-AS1	1133	HSALNG0015539	1924	UGDH	2715	RAD23A
343	PLA2G7	1134	piR-61101-324	1925	TNFRSF10D	2716	REST
344	IL12B	1135	LOC124903741	1926	TYROBP	2717	SMS
345	ALB	1136	TMPO	1927	SST	2718	ACD
346	TNFSF12	1137	UNC13D	1928	TAB3	2719	ACKR3
347	HLA-C	1138	SMAD2	1929	MANF	2720	ASCC1
348	AFF3	1139	ABCG2	1930	CLEC4A	2721	ASH2L
349	OLIG3	1140	SPARC	1931	DNAH8	2722	BMP3
350	CDK6	1141	FAP	1932	SLAMF8	2723	CAV2
351	IL33	1142	VIPR1	1933	FCGBP	2724	CD84
352	TNFRSF13B	1143	SEMA3C	1934	MIAT	2725	DDAH1
353	CXCL13	1144	COL9A2	1935	MIR138-2	2726	EREG
354	IRAK1	1145	TANK	1936	MIR4707	2727	F11R
355	TYK2	1146	LILRA3	1937	MIR5695	2728	GJB6
356	RBPJ	1147	TAF3	1938	SDHB	2729	HGFAC
357	RAG2	1148	CYP3A4	1939	RPS26	2730	LPAR3
358	TNFRSF14	1149	NTRK1	1940	ILF3	2731	MAP3K4
359	PSMB9	1150	CYLD	1941	HLA-F-AS1	2732	MTMR3
360	C4B	1151	C1QBP	1942	PADI1	2733	NLRP2
361	LEP	1152	DRD1	1943	CDKN2A	2734	NOX4
362	NFKB2	1153	BAD	1944	HSPB1	2735	OPRD1
363	RUNX1	1154	GRK5	1945	C1S	2736	PCDH15
364	ETS1	1155	ATG16L1	1946	HEXB	2737	PCSK6
365	PON1	1156	MED1	1947	SCARB1	2738	PRDX4
366	ACE	1157	PCBP1	1948	MASP2	2739	PYCARD
367	MMEL1	1158	CIRBP	1949	B4GALT1	2740	RASGRF1
368	LINC00426	1159	GGT6	1950	GP6	2741	RPTOR
369	LINC02384	1160	SMTNL2	1951	MB	2742	SDHAF2
370	DPP4	1161	XRCC4	1952	PIM2	2743	SLC8A3
371	BCL2	1162	LPIN2	1953	NFAT5	2744	TFF1
372	SPRED2	1163	TMSB4X	1954	PDPN	2745	THOP1
373	IGKC	1164	ADGRE5	1955	GABPA	2746	WNT9A
374	CXCR2P1	1165	AXL	1956	ID3	2747	ADIPOR2

375	PVT1	1166	STAT5A	1957	SNTB1	2748	BRD1
376	CD8A	1167	BCL6	1958	SNHG32	2749	CAPZA1
377	GSTM1	1168	WARS1	1959	SMAD5-AS1	2750	CENPJ
378	FGFR1	1169	PPIA	1960	MEG8	2751	DDAH2
379	TAP1	1170	CYB5A	1961	SNHG6	2752	DHRS3
380	BLTP1	1171	IFI16	1962	SNORA75	2753	FABP2
381	GCH1	1172	LTB4R	1963	RPS2P14	2754	MINK1
382	HLA-DRB5	1173	SLAMF7	1964	ANXA11	2755	NIN
383	MIR125A	1174	LGALS3BP	1965	SCARNA5	2756	RND3
384	HLA-G	1175	MATN1	1966	CHST11	2757	SEL1L
385	IL4R	1176	IL36A	1967	SLC2A10	2758	SERPINA7
386	CCN1	1177	MIR155HG	1968	CAPZB	2759	SMG1
387	NFKBIE	1178	MIR106B	1969	RYR1	2760	TRIM2
388	CD19	1179	MIR34C	1970	CTSD	2761	TRIM63
389	CD226	1180	MIR449A	1971	NT5E	2762	TTPA
390	CD274	1181	MIR373	1972	ALPL	2763	UNC5C
391	WAS	1182	MIR425	1973	LEPR	2764	VARS2
392	ICAM3	1183	MIR766	1974	RPS6KB1	2765	VNN2
393	KIF5A	1184	LETR1	1975	NR2F2	2766	BHLHE40
394	CR2	1185	TNC	1976	ANXA2	2767	BHLHE41
395	MEG3	1186	PPARA	1977	CD46	2768	C9orf72
396	NOS3	1187	ALOX5AP	1978	KCNN4	2769	CCN3
397	MS4A1	1188	PF4	1979	NFATC2	2770	CLDN5
398	IGF1	1189	CD52	1980	PRDX2	2771	CPVL
399	PRKCH	1190	IKZF1	1981	TBX5	2772	FGL1
400	TNPO3	1191	HRAS	1982	RAP1A	2773	FSTL3
401	GATA3	1192	BMP2	1983	P2RY11	2774	HIPK3
402	CERNA3	1193	FOXO1	1984	LAP3	2775	ITGAE
403	HLA-DQB2	1194	TYRO3	1985	SDC3	2776	KLK11
404	IL17F	1195	OGG1	1986	SLC46A1	2777	MAP1LC3A
405	MMP19	1196	EZR	1987	DPP7	2778	MBD2
406	RAG1	1197	FADD	1988	JUNB	2779	MC3R
407	TACR3	1198	CISH	1989	PROCR	2780	MEOX2
408	AIF1	1199	CUL1	1990	LILRA2	2781	MTDH
409	CALCA	1200	FCN3	1991	CEMIP	2782	NUCB2
410	HIF1A	1201	RAMP2	1992	SIGLEC9	2783	PITRM1
411	BIRC5	1202	SPI1	1993	IL17C	2784	PPP1R15A
412	DKK1	1203	OLR1	1994	CCL15	2785	PSME3
413	FGF2	1204	TACR2	1995	MUC7	2786	PTGER1
414	TNFSF4	1205	AIM2	1996	OIP5	2787	REG1A
415	NAT2	1206	CD160	1997	THNSL2	2788	RUBCN
416	ZAP70	1207	IL17RC	1998	MIR29A	2789	SC5D
417	B3GAT1	1208	PRRT1	1999	MIR34B	2790	STK16
418	GSTP1	1209	MIR214	2000	LINC-PINT	2791	SULF2

419	SH2B3	1210	TRA	2001	MIR138-1	2792	TIGIT
420	TTC7A	1211	MIR199A2	2002	MIR92A1	2793	TPST1
421	PARN	1212	MIR99B	2003	MIR24-1	2794	TRAIP
422	CD2	1213	RUNX1-IT1	2004	RAB4B-EGLN2	2795	TRIM22
423	CALR	1214	KC6	2005	MIR448	2796	TRPV2
424	HLA-DOA	1215	LOC111365141	2006	TRE-TTC3-1	2797	BOK
425	IRF4	1216	EXOSC10	2007	PEX5	2798	ELL
426	SERPINE1	1217	ADAMTS12	2008	XRCC6	2799	FAM13A
427	NOTCH4	1218	CAMP	2009	FGB	2800	GPBAR1
428	MYC	1219	GAST	2010	KLF5	2801	KHDRBS3
429	ADA	1220	IGES	2011	ADAMTSL2	2802	LAT2
430	CYP21A1P	1221	QDPR	2012	MMP28	2803	NCR1
431	HMOX1	1222	ERAP2	2013	LY6G5C	2804	NCR2
432	MTRR	1223	CASP9	2014	CNNM3-DT	2805	SEC14L2
433	RAD51B	1224	IL36B	2015	TOR1A	2806	SEMA3B
434	TRIM21	1225	GRM5	2016	MAGT1	2807	BAIAP2L1
435	FAM167A	1226	NDUFA13	2017	PLA2G1B	2808	BICD1
436	TSBP1	1227	PTPRM	2018	IRGM	2809	CENPA
437	GGH	1228	PDCD1LG2	2019	HDAC6	2810	CTBS
438	INS	1229	MTF1	2020	INSR	2811	ESCO2
439	MTR	1230	RPL31	2021	FGFR4	2812	FCRL2
440	ELMO2	1231	CDK5RAP2	2022	HSD11B1	2813	GP2
441	FN1	1232	OLFM4	2023	SQSTM1	2814	GSDME
442	IRF8	1233	SIRT4	2024	DICER1	2815	IL13RA2
443	MIR21	1234	PPT2	2025	GFAP	2816	IL25
444	KCNJ11	1235	RABEP1	2026	HDAC9	2817	KCNK5
445	RELN	1236	HSPA1B	2027	KEAP1	2818	MIA3
446	NTAN1	1237	ITLN1	2028	LRP1	2819	MSMB
447	HAVCR2	1238	PPM1L	2029	PRKN	2820	NLRP6
448	LINC-ROR	1239	CASC3	2030	BIRC2	2821	PGLYRP2
449	HLA-A	1240	CLYBL	2031	FTO	2822	SLCO1A2
450	SUMO4	1241	FADS3	2032	IRS1	2823	SUCNR1
451	ADRB2	1242	PPIL4	2033	LAMC2	2824	SV2B
452	HTR2A	1243	RNASEH2B	2034	TGFB3	2825	TOR2A
453	CD81	1244	FAM124A	2035	TPI1	2826	BTG3
454	TNXB	1245	PHRF1	2036	BIRC3	2827	CCL25
455	GSTT1	1246	ANO8	2037	CNR1	2828	CCNG2
456	PXK	1247	DDA1	2038	COL4A2	2829	CYP4F22
457	CD14	1248	FLACC1	2039	PRKAA1	2830	EPC1
458	ESR2	1249	AHNAK2	2040	PTGER3	2831	GNLY
459	CD83	1250	GUCY1B2	2041	RUNX2	2832	HSPB6
460	DCLRE1C	1251	SMIM21	2042	ARNT	2833	IER3
461	TNFSF15	1252	BMS1P20	2043	C1QC	2834	IFI27
462	UBASH3A	1253	LINC03033	2044	CD74	2835	IFNLR1

463	PLAT	1254	HLA-S	2045	FGF5	2836	LILRA5
464	VWF	1255	RPL17P22	2046	GAL	2837	LRRC15
465	LGALS9	1256	IRAK3	2047	HBEGF	2838	MAPRE3
466	ARID5B	1257	PLAA	2048	MMP16	2839	PDZD2
467	ITPA	1258	CXCL11	2049	C1QB	2840	PIWIL2
468	MTOR	1259	S100A1	2050	E2F1	2841	PIWIL4
469	ATG5	1260	LPL	2051	HYAL2	2842	SAP30BP
470	RTKN2	1261	PGK1	2052	ID2	2843	SCG5
471	IL5	1262	PIK3R2	2053	TICAM1	2844	TRO
472	PLD4	1263	FOLR1	2054	ALCAM	2845	UBD
473	TMX2-CTNND1	1264	RB1	2055	BCL2L2	2846	ZC3HC1
474	LTB	1265	PIN1	2056	DHX9	2847	ATP6V0E2
475	CP	1266	SPRY2	2057	GAB2	2848	CBLN2
476	LBH	1267	CNR2	2058	NR2C2	2849	CHID1
477	LGALS3	1268	IREB2	2059	SLIT3	2850	DPH6
478	HLA-DRB6	1269	MTNR1B	2060	IL17RB	2851	EBAG9
479	MIR223	1270	CHGA	2061	MCAM	2852	EMCN
480	NPY	1271	FOXC1	2062	RPL7	2853	ENGASE
481	COG6	1272	ADRB3	2063	ADAMTS9	2854	GADD45B
482	SOD1	1273	FABP4	2064	BNIP3	2855	GBP5
483	PRDM1	1274	ABCC11	2065	CDKAL1	2856	GLCCI1
484	PSTPIP1	1275	MPG	2066	RECK	2857	MAL
485	CCL13	1276	MSRA	2067	TFF3	2858	TNIP3
486	CCL19	1277	WDR26	2068	TRIM38	2859	BLTP3A
487	IL12A	1278	CPEB4	2069	SCUBE2	2860	CCL14
488	H2AC18	1279	SH2D2A	2070	CLEC2D	2861	CGAS
489	TLR7	1280	TIMD4	2071	DNAJA2	2862	CNTNAP4
490	PICSAR	1281	CNMD	2072	LAMP3	2863	FOXJ3
491	VIP	1282	RASGRP4	2073	A1BG	2864	IFNL2
492	KIT	1283	NKIRAS1	2074	CHAD	2865	MAGEA1
493	CASP8	1284	MIR149	2075	CTF1	2866	MRPL17
494	BDNF-AS	1285	MIR191	2076	LILRA1	2867	PTTG1IP
495	CCL11	1286	MIR370	2077	CCL23	2868	TAS2R38
496	F3	1287	MIR346	2078	MIR27B	2869	TESPA1
497	VIM	1288	MIR106A	2079	MIR93	2870	VGLL3
498	CFLAR	1289	MIR103A1	2080	MIR19A	2871	VSTM1
499	WAKMAR2	1290	MIR410	2081	SNHG1	2872	ALKBH5
500	GPT	1291	MIR7-2	2082	MIR424	2873	C1orf87
501	SRC	1292	MIR532	2083	MIR423	2874	DERL1
502	EPO	1293	ZNF667-AS1	2084	BACE1-AS	2875	EBF4
503	ESR1	1294	LINC00638	2085	RNY5	2876	LY6G6C
504	APOM	1295	LINC01193	2086	LOC102723407	2877	METRNL
505	XDH	1296	VPS33B-DT	2087	ST2	2878	MMD
506	IMPDH2	1297	PPL	2088	IDDM8	2879	PALS2

507	C5-OT1	1298	EVPL	2089	LOC106780803	2880	PCNP
508	TNFSF10	1299	IGHV4-38-2	2090	LOC106780804	2881	TTC38
509	MECP2	1300	IGFBP2	2091	DDC	2882	CLEC5A
510	AR	1301	CD22	2092	AKT3	2883	CST2
511	TF	1302	PDE4A	2093	ACHE	2884	IFI44L
512	UBE2L3	1303	MDC1	2094	HRH2	2885	LEAP2
513	EGFR	1304	RPL3	2095	HNRNPDL	2886	CASP12
514	PIK3CD	1305	SKIC2	2096	TGFA	2887	NKAPL
515	LCK	1306	CDH22	2097	C4BPB	2888	TNFAIP8L2
516	MTCO3P1	1307	XCR1	2098	ARHGAP45	2889	ENHO
517	MMP7	1308	DCAF5	2099	TMEM258	2890	KLLN
518	FLI1	1309	MIR629	2100	IFNAR2	2891	OR6C4
519	S100A4	1310	POU5F1	2101	IFNAR1	2892	IGSF22
520	CD27	1311	FOXF1	2102	ZEB1	2893	DLEU1
521	CXCL16	1312	RBM17	2103	CDK12	2894	PBOV1
522	PPARG	1313	CEP68	2104	GALT	2895	FAM215A
523	PRKCD	1314	CLIC6	2105	KCNE1	2896	LINC00305
524	PIP4K2C	1315	PUS10	2106	MAPKAPK5	2897	MIR10B
525	IRF1	1316	SEL1L3	2107	IL11RA	2898	MIRLET7A1
526	POMC	1317	PSORS1C2	2108	STAMBP	2899	UMAD1
527	KITLG	1318	B3GALT9	2109	TMPRSS6	2900	HAND2-AS1
528	TNFRSF17	1319	ETS1-AS1	2110	ACSF3	2901	IGHV1-69
529	LBR	1320	LINC02664	2111	ACTG2	2902	MAPKAPK5-AS1
530	CENPB	1321	LOC101929538	2112	CDK10	2903	MIR125B2
531	CYP21A2	1322	TSBP1-AS1	2113	GART	2904	MIR208B
532	CD1D	1323	ENSG00000272501	2114	SETD1A	2905	MIR219A1
533	IL27	1324	ENSG00000232638	2115	STX4	2906	MIR29B1
534	CD55	1325	ENSG00000232693	2116	VANGL2	2907	MIR326
535	GTF2I	1326	TREHP1	2117	BRAP	2908	MIR378A
536	LSM2	1327	ENSG00000269667	2118	CBFA2T3	2909	MIR431
537	AKT1	1328	ENSG00000284825	2119	CTDP1	2910	MIR99A
538	BRD2	1329	ENSG00000285163	2120	IGSF3	2911	PAPPA-AS1
539	PERP	1330	SETP16	2121	TET3	2912	GUSBP1
540	PTGER4	1331	ENSG00000285040	2122	ABCF1	2913	MIR15B
541	IKZF3	1332	HSALNG0074148-001	2123	EXOC2	2914	MIR25
542	SLC22A5	1333	HSALNG0150715	2124	FIGNL1	2915	MIR26A2
543	HAVCR1	1334	HSALNG0150716	2125	HLA-F	2916	MIR301A
544	MMP10	1335	NONHSAG005179.2-002	2126	HSD17B8	2917	MIR339
545	CCN2	1336	lnc-RTKN2-5	2127	MVP	2918	MIR342
546	NPPB	1337	piR-56037-112	2128	PPIP5K2	2919	MIRLET7A2
547	ATM	1338	CM034954-069	2129	PPP1R10	2920	MIRLET7BHG
548	HLA-DQB1-AS1	1339	CM034966-334	2130	RAB5B	2921	SNORD118
549	PLAUR	1340	HSALNG0049423	2131	RNF40	2922	TSPEAR-AS2

550	SLC17A5	1341	RF00951-116	2132	SON	2923	MIR101-1
551	MIR126	1342	lnc-HLA-DQA1-9	2133	SRCAP	2924	MIR128-1
552	PSORS1C3	1343	lnc-HLA-DRB1-7	2134	SRSF5	2925	MIR129-2
553	SERPINA1	1344	lnc-ICAM3-1	2135	SWAP70	2926	MIR154
554	DRAIC	1345	piR-32314-017	2136	BMP2K	2927	MIR181A1
555	HLA-DRB9	1346	CM021583-048	2137	FYCO1	2928	MIR215
556	CFI	1347	lnc-ICAM3-2	2138	BOLA3	2929	MIR33B
557	IBSP	1348	piR-33103-025	2139	COX6C	2930	MIR582
558	CD38	1349	piR-39858-769	2140	DCTN4	2931	MIR92B
559	F2RL1	1350	CXCR1	2141	PAPOLG	2932	SNHG17
560	IGFBP1	1351	IL2RG	2142	PBX2	2933	CACNA1G-AS1
561	TEK	1352	MSN	2143	SESN3	2934	DIAPH2-AS1
562	MIR499A	1353	TGM2	2144	SLC37A1	2935	LINC00342
563	YDJC	1354	PLG	2145	SRPK3	2936	MIR218-1
564	PSORS1C1	1355	PTK2B	2146	FNIP1	2937	MIR432
565	CCL18	1356	CYCS	2147	MPPE1	2938	MIR449B
566	PLAU	1357	FOXO3	2148	NUDT12	2939	MIR485
567	GPSM3	1358	TXNRD1	2149	RBM22	2940	MIR487B
568	RNPC3	1359	ADIPOR1	2150	SLC36A1	2941	MIR7-3
569	HGF	1360	ITGA1	2151	SLU7	2942	MIR760
570	APOE	1361	PLA2G5	2152	SNAPC4	2943	MIRLET7F1
571	RPLP1	1362	CLEC12A	2153	WTAP	2944	FOXD2-AS1
572	ITGB1	1363	CXCL2	2154	ATP10B	2945	HAR1B
573	SELP	1364	KLRC2	2155	BOLL	2946	LINC00452
574	EMSLR	1365	MIR7-3HG	2156	EMSY	2947	LINC01503
575	TAB1	1366	CCL15-CCL14	2157	GATAD1	2948	MIR1915
576	IL15RA	1367	HLA-DRB2	2158	IKZF4	2949	MIR224
577	PTH	1368	SMAD7	2159	SDF2L1	2950	MIR3074
578	P2RX7	1369	PIBF1	2160	SLC25A28	2951	MIR495
579	HPRT1	1370	MFAP4	2161	TRMT112	2952	MIR500A
580	IL20	1371	RPS6KA5	2162	ABT1	2953	MIR518A1
581	GC	1372	ADAM10	2163	BCL7C	2954	MIR7-1
582	TAC1	1373	TGFB2	2164	CYRIB	2955	RNY1
583	DDX6	1374	GGT1	2165	GPR174	2956	SAA3P
584	SUOX	1375	ABCA1	2166	ORAI3	2957	IGKV2D-29
585	B3GNT2	1376	TYMP	2167	RNF166	2958	LINC00649
586	XK	1377	BMP7	2168	SERBP1	2959	MIATNB
587	CD163	1378	TCF7L2	2169	SNAPC1	2960	MIR1286
588	ZFAT-AS1	1379	CHRNA7	2170	SPIRE2	2961	MIR3127
589	HFE	1380	CNTF	2171	CCDC69	2962	MIR4516
590	HLA-DOB	1381	CTHRC1	2172	FAM76B	2963	MT-TT
591	APOA1	1382	LAG3	2173	GSDMC	2964	RNU1-1
592	EDN1	1383	UCN	2174	KIF26A	2965	FAS-AS1
593	MIR146B	1384	MIR3928	2175	LRRC2	2966	GAS6-DT

594	CASP3	1385	IFN1@	2176	MAGI3	2967	GCSIR
595	NFATC1	1386	APP	2177	RGS22	2968	HISLA
596	AMPD1	1387	CYP2D6	2178	TMEM50B	2969	LINC00630
597	HLA-DPA1	1388	HTR3A	2179	TTC33	2970	LINC00968
598	CD58	1389	FCGRT	2180	ZNF689	2971	LINC01010
599	PRDX5	1390	MFGE8	2181	TXNDC16	2972	MIR376B
600	CYP2C19	1391	HOXD13	2182	ZKSCAN3	2973	MIR4423
601	LEPQTL1	1392	MIR30A	2183	ZSCAN12	2974	MIR500B
602	SNORD44	1393	MUSK	2184	FBRS	2975	MIR545
603	TNFRSF25	1394	AQP5	2185	HMGN4	2976	MIR941-2
604	ADAD1	1395	DPYSL5	2186	PHETA1	2977	CFTR-AS1
605	THBD	1396	CENPC	2187	PRR14	2978	LCMT1-AS1
606	ANGPT1	1397	EPHB4	2188	RPP25L	2979	LINC01483
607	IL34	1398	EPHA2	2189	ZKSCAN8	2980	MIR1298
608	MIR543	1399	HDAC2	2190	ZNF764	2981	MIR523
609	IL9	1400	CXCR2	2191	ZNF778	2982	MIR642B
610	TACR1	1401	MST1R	2192	ZSCAN9	2983	NRXN1-DT
611	PSMB8	1402	RPS19	2193	INO80E	2984	MIR297
612	PLCL2	1403	EPHA1	2194	PHF24	2985	MIR376A2
613	ZPBP2	1404	NGFR	2195	ZSCAN31	2986	MIR4508
614	MBP	1405	NRP1	2196	SPATA2L	2987	LINC01195
615	ARL15	1406	DDR1	2197	ZNF688	2988	MIR3162
616	TAPBP	1407	IL1R2	2198	ABHD16B	2989	MIR3196
617	CDKN1A	1408	SERPINF2	2199	FAM133B	2990	MIR378G
618	MIR22	1409	MDK	2200	SFTA2	2991	CLYBL-AS3
619	SIRT1	1410	DNASE2	2201	SLX1A	2992	LOC102723883
620	SLX1A-SULT1A3	1411	BBC3	2202	C14orf180	2993	MIR1261
621	CA2	1412	ID1	2203	CFAP119	2994	MIR378D1
622	FLT3	1413	TNFRSF10C	2204	TMEM116	2995	MIR4739
623	MSH5	1414	MPIG6B	2205	ZSCAN23	2996	MIR550B1
624	ATXN2	1415	HEXD	2206	DEFB136	2997	MIR5571
625	U2AF1	1416	SFTA3	2207	TTC34	2998	ENSG00000231769
626	PADI6	1417	MIR197	2208	HTD2	2999	ENSG00000236525
627	PTX3	1418	HCCAT5	2209	MUC22	3000	ENSG00000261433
628	GSDMB	1419	MKNK1	2210	MIR9-2HG	3001	ENSG00000261573
629	ANGPT2	1420	POLR1C	2211	HCG27	3002	ENSG00000197585
630	CPT2	1421	SENP1	2212	LEF1-AS1	3003	ENSG00000261976
631	GNRHR	1422	RBM5	2213	MIR4435-2HG	3004	ENSG00000266680
632	CHD7	1423	BTN3A2	2214	JAZF1-AS1	3005	HSPE1P1
633	NLGN3	1424	AGRN	2215	SNORD117	3006	RPL31P31
634	WIPF1	1425	PLP1	2216	MIR181A1HG	3007	CYP4F23P
635	IRF2BP2	1426	LRBA	2217	SNAI3-AS1	3008	LOC101928120
636	LIN54	1427	ITGB3	2218	TMEM161B-DT	3009	MRPS35P1
637	ZNF354A	1428	MAP3K5	2219	ADAM1A	3010	CDR1-AS

638	USP50	1429	DCN	2220	BOLA3-DT	3011	LOC111674472
639	FRG2C	1430	DHCR7	2221	C2orf74-DT	3012	MS
640	TSIX	1431	ASIC1	2222	LINC00977	3013	LOC110973015
641	ATOD1	1432	CDH13	2223	PRKCQ-AS1	3014	NTT
642	SYNGR1	1433	IRF3	2224	TNFRSF14-AS1	3015	GLA
643	IL7R	1434	TRAF3IP2	2225	ACOXL-AS1	3016	CYP27B1
644	DNTT	1435	CD9	2226	C20orf181	3017	ADAMTS5
645	ELMO1	1436	CA3	2227	HCFC1-AS1	3018	RNASE3
646	CXCR6	1437	LTB4R2	2228	LINC01307	3019	PCDHGC4
647	KIR2DS2	1438	ORM1	2229	LINC02649	3020	SACS
648	MIR498	1439	CD68	2230	MIR3142HG	3021	MAF
649	IFNGR2	1440	SELENOS	2231	CD101-AS1	3022	PTPRN
650	TMEM187	1441	VSIG4	2232	IFNAR2-IL10RB	3023	SMARCA4
651	ABCC1	1442	XCL1	2233	LINC01303	3024	ACTN1
652	CASP1	1443	MIR10A	2234	LINC02098	3025	NEU1
653	ANXA3	1444	OIP5-AS1	2235	PLCL2-AS1	3026	SKI
654	POU3F1	1445	MIR338	2236	LINC01980	3027	GOT1
655	RELA	1446	MIR361	2237	LINC02060	3028	NDRG1
656	SOD2	1447	MIR650	2238	LINC02571	3029	DRD4
657	MIR16-1	1448	TRD	2239	LINC02757	3030	RACK1
658	AHR	1449	UGT1A1	2240	LNCRNA-IUR	3031	SKP1
659	STAT6	1450	LAT	2241	LOC100287329	3032	DSCAM
660	CYP1A2	1451	LCAT	2242	ZNF747-DT	3033	SH3PXD2A
661	EGF	1452	TNNT2	2243	LINC00709	3034	UBR5
662	GZMA	1453	PLOD2	2244	LINC02676	3035	ARID3A
663	MIR133B	1454	AMH	2245	LOC102723878	3036	ICAM4
664	CYTOR	1455	CYP3A5	2246	SMG1P5	3037	MEF2B
665	RPA3	1456	MMP17	2247	UQCRHP1	3038	UMODL1
666	ANGEL2	1457	PREP	2248	ENSG00000269954	3039	CTIF
667	PRKCB	1458	PROK2	2249	LINC02341	3040	HIVEP3
668	ZNF774	1459	IAPP	2250	MIR6891	3041	BORCS8-MEF2B
669	MAP3K7	1460	SPN	2251	PCNPP1	3042	LNCPRESS1
670	NEAT1	1461	NCR3	2252	STK19B	3043	LINC01475
671	WDFY4	1462	GLS	2253	TXNDC11-AS1	3044	EEF1B2P6
672	ENSG00000083622	1463	RXRB	2254	DDX18P1	3045	TOP1
673	PARP1	1464	PEX13	2255	ENSG00000217702	3046	FBN1
674	LINC02656	1465	ZFYVE26	2256	ENSG00000228005	3047	SNRPA
675	GSN	1466	CCHCR1	2257	ENSG00000228509	3048	ERAP1
676	DNASE1L3	1467	LIMD1	2258	ENSG00000254084	3049	HRH1
677	CD101	1468	NAB1	2259	ENSG00000255422	3050	GAD2
678	FCGR2C	1469	UPK2	2260	ENSG00000267199	3051	CFP
679	KLF12	1470	KIN	2261	ENSG00000271904	3052	CCK
680	IL17D	1471	PHLDB1	2262	ENSG00000272449	3053	ADAMTS7
681	CYP2C9	1472	TCF19	2263	ENSG00000272540	3054	COMT

682	CCL26	1473	VPS52	2264	RNU6-474P	3055	TSHR
683	CST3	1474	BCL9L	2265	RPL32P23	3056	CPOX
684	SOST	1475	CEP76	2266	RPS12P3	3057	RHOD
685	TIMELESS	1476	FAM98B	2267	ZNF603P	3058	SLC2A1
686	SALL3	1477	FOXR1	2268	ALG1L11P	3059	TSC2
687	NGF	1478	AHSA2P	2269	ENSG00000223808	3060	LIG4
688	TH	1479	LINC02694	2270	ENSG00000224478	3061	MVK
689	LINC01104	1480	HCG22	2271	ENSG00000235237	3062	SLC3A1
690	MCL1	1481	HCG23	2272	ENSG00000259989	3063	TSC1
691	MUC1	1482	SENCR	2273	ENSG00000260267	3064	CHRNA1
692	CDK2	1483	CCR5AS	2274	ENSG00000260304	3065	GRHPR
693	IKBKE	1484	LINC01882	2275	GLULP4	3066	SYP
694	DUSP1	1485	LINC01343	2276	RN7SL51P	3067	AGXT
695	HRH4	1486	LINC03004	2277	RNU4ATAC4P	3068	CHRNE
696	NLRC4	1487	PRDX6-AS1	2278	RNU6-979P	3069	CUBN
697	FCER2	1488	TDH-AS1	2279	ENSG00000201207	3070	DMD
698	CAMLG	1489	IL6ST-DT	2280	ENSG00000287564	3071	SLC2A9
699	FCRL4	1490	LINC02979	2281	ENSG00000288711	3072	MSX1
700	MIR142	1491	RPL3P2	2282	LOC101928272	3073	PRODH
701	ACP1	1492	ENSG00000250264	2283	LOC107984709	3074	SLC22A12
702	TREH	1493	LOC112267968	2284	P2RY10BP	3075	MOCS2
703	ZNF438	1494	ENSG00000226032	2285	POLR2LP1	3076	SLC7A9
704	SSB	1495	LINC02498	2286	RN7SL563P	3077	CDH17
705	XIAP	1496	LINC02786	2287	RNU7-133P	3078	DSC3
706	CYP19A1	1497	RN7SL688P	2288	UQCRC2P1	3079	DST
707	C6orf47	1498	ENSG00000222529	2289	CDKN2AIPNLP3	3080	IRF6
708	CSNK2B	1499	PTPN11P3	2290	ENSG00000285842	3081	HOGA1
709	BAG6	1500	BTF3L4P3	2291	ENSG00000288583	3082	MOCOS
710	TGIF1	1501	ENSG00000284957	2292	RN7SL335P	3083	NHEJ1
711	KRT18P39	1502	LOC124901411	2293	VDAC1P9	3084	PTMA
712	IGHG2	1503	ENSG00000284914	2294	lnc-HLA-C-2	3085	RAPSN
713	AKT2	1504	lnc-IL31RA-5	2295	lnc-KCNE1-1	3086	MOCS1
714	PTEN	1505	CM034956-314	2296	lnc-LEF1-1	3087	UTRN
715	PDGFB	1506	CM034960-050	2297	ENSG00000286742	3088	CALB2
716	MIR140	1507	ENSG00000289525	2298	ENSG00000288064	3089	LYST
717	CUTALP	1508	HSALNG0050281	2299	lnc-FAM76B-1	3090	MMP26
718	HLA-DQB3	1509	HSALNG0076038	2300	lnc-SEH1L-5	3091	SUCO
719	PTPN11	1510	HSALNG0076040	2301	CM021573-028	3092	PAXX
720	HSPA1A	1511	HSALNG0146108	2302	CM034951-025	3093	KIAA2026
721	TAB2	1512	HSALNG0149394	2303	CM034952-323	3094	THM
722	PTGES	1513	HSALNG0150714	2304	CM034972-150	3095	SMAD4
723	NAGLU	1514	MN298114-173	2305	ENSG00000285552	3096	PAK1
724	LGALS2	1515	NONHSAG005179.2-001	2306	ENSG00000285616	3097	ENPP1

725	SPATA31F1	1516	RF00017-4664	2307	ENSG00000289626	3098	GATA1
726	ALOX15	1517	RF00019-050	2308	ENSG00000289970	3099	TPT1
727	TSLP	1518	lnc-IL6ST-2	2309	HSALNG0015258	3100	CFD
728	PPBP	1519	lnc-OLIG3-1	2310	HSALNG0015259	3101	LRPAP1
729	S1PR1	1520	CM034956-345	2311	HSALNG0020226-444	3102	CEACAM5
730	FAM107A	1521	CM034958-060	2312	HSALNG0035397	3103	RICTOR
731	ELN	1522	HSALNG0055144	2313	HSALNG0049258	3104	SERPINB5
732	CALCR	1523	HSALNG0074144	2314	HSALNG0057943	3105	TRADD
733	MIR98	1524	HSALNG0142585	2315	HSALNG0077080-001	3106	RPN2
734	IFIH1	1525	LOC105369568	2316	HSALNG0102112	3107	LMO2
735	TP63	1526	LOC107984788	2317	HSALNG0102114	3108	SARM1
736	ACTR2	1527	NONHSAG031883.2-001	2318	HSALNG0102115	3109	HOXA5
737	RNF182	1528	NONHSAG031883.2-002	2319	HSALNG0102116	3110	DDX18
738	BDNF	1529	RF00001-253	2320	HSALNG0106856-001	3111	MUC16
739	DDR2	1530	PROC	2321	HSALNG0113565	3112	NAV2
740	RHOA	1531	DNMT3B	2322	HSALNG0120134	3113	SLC30A6
741	IL1RL1	1532	EPAS1	2323	HSALNG0120135	3114	ZNF804A
742	MET	1533	ENTPD1	2324	HSALNG0147405	3115	SPZ1
743	LRP5	1534	KRT18	2325	LOC105376481	3116	MIR187
744	YWHAH	1535	ABCC2	2326	LOC124901069	3117	MIR1237
745	ELP1	1536	CD3G	2327	LOC124907867	3118	INE2
746	MIR34A	1537	CFB	2328	LOC645481	3119	E2F3-IT1
747	MIR17	1538	KL	2329	MN298114-111	3120	AGTR1
748	MIR451A	1539	RDX	2330	NONHSAG034948.2	3121	IKBKG
749	LERFS	1540	AQP1	2331	NONHSAG043472.2	3122	CNTNAP2
750	MAP3K14	1541	C1QA	2332	NONHSAG044958.2	3123	HSD3B2
751	JAK2	1542	CYBA	2333	RF00017-5052	3124	PDYN
752	PDE2A	1543	NR1H3	2334	lnc-AFF3-1	3125	TPH1
753	MAPKAPK2	1544	ITGA2	2335	lnc-ANXA6-3	3126	HGD
754	FADS1	1545	XBP1	2336	lnc-CHP2-1	3127	P2RX3
755	GPX3	1546	MMP11	2337	lnc-GIN1-2	3128	PLEK
756	MAP3K1	1547	IGFBP5	2338	lnc-IL2RB-1	3129	PRG2
757	SUMO1	1548	AGFG1	2339	lnc-KIN-10	3130	SPINK5
758	LILRB1	1549	CCR9	2340	lnc-MECP2-2	3131	CCL27
759	TREM1	1550	TCN1	2341	lnc-RAD51B-6	3132	DEFB4A
760	MIR143	1551	DLG5	2342	lnc-RCAN1-1	3133	DEFB103B
761	CDK4	1552	IL18RAP	2343	lnc-SNN-1	3134	PEP
762	CYP17A1	1553	DEFA1	2344	lnc-TNFRSF14-1	3135	NAIL
763	SLCO1B1	1554	HLA-DRB3	2345	piR-43107-154	3136	CSIF

764	GSR	1555	HOTTIP	2346	piR-47864	3137	OCTN1
765	PNP	1556	MIR135B	2347	piR-52740	3138	SLAMF4
766	TIE1	1557	MIR26A1	2348	piR-53431-298	3139	TNFB
767	TRA-TGC7-1	1558	MIR193A	2349	CM034956-273	3140	LYP
768	PSMD5	1559	MIR23B	2350	CM034959-072	3141	IFNB2
769	LINC02357	1560	MIR31	2351	CM034961-235	3142	C13orf31
770	RASGRP1	1561	MIR18A	2352	CM034965-164	3143	PSTPIP
771	YPEL5	1562	CBR3-AS1	2353	HSALNG0017286	3144	MHC2TA
772	CLNK	1563	MIR129-1	2354	HSALNG0017867	3145	DEFCAP
773	ACOXL	1564	MIR494	2355	HSALNG0033465	3146	NALP1
774	ADORA2A	1565	SNORD95	2356	HSALNG0045887	3147	VAMAS1
775	MTHFD1	1566	TRG	2357	HSALNG0049410	3148	AIADK
776	SHMT1	1567	TRC-GCA24-1	2358	HSALNG0067266	3149	HSF
777	XRCC1	1568	IGLV@	2359	HSALNG0068535	3150	PTPN8
778	PRKG1-AS1	1569	ALDH2	2360	HSALNG0068536-001	3151	NKR2B4
779	KDR	1570	SLC29A1	2361	HSALNG0094038	3152	GVHDS
780	NCF4	1571	VDAC1	2362	HSALNG0095990	3153	PAPAS
781	TAX1BP1	1572	IL12RB2	2363	HSALNG0102522	3154	BSF2
782	JAZF1	1573	ORMDL3	2364	HSALNG0106869	3155	JUVAR
783	DUSP22	1574	ZNF318	2365	HSALNG0132884-001	3156	CD2BP1
784	ERBB2	1575	BMP4	2366	HSALNG0141944	3157	C2TA
785	TEC	1576	COL1A1	2367	HSALNG0143727	3158	CARD7
786	RBP4	1577	PLD1	2368	HSALNG0144556	3159	KIAA0926
787	MAPK10	1578	KCNMA1	2369	HSALNG0148972-001	3160	MSPC
788	HAMP	1579	CYP1A1	2370	HSALNG0148972-002	3161	JRRP
789	RARRES2	1580	ENO2	2371	HSALNG0149134	3162	RA
790	IFNL1	1581	HSP90B1	2372	LOC105376483		
791	LST1	1582	CLEC7A	2373	MK280144-020		

Table S5. 288 putative targets of ZDS against RA.

NO.	Target	NO.	Target	NO.	Target	NO.	Target
1	TNF	73	ZAP70	145	TERT	217	TGFBR2
2	MIF	74	GSTP1	146	PRKDC	218	PLK1
3	IL2	75	ADA	147	HDAC1	219	HDAC3
4	MMP3	76	HMOX1	148	RORC	220	CDK1
5	IL6ST	77	ESR2	149	TRPV1	221	SIRT3
6	MMP1	78	PLAT	150	QPCT	222	CYP11B2
7	PTGS2	79	LGALS9	151	PFKFB3	223	MTNR1A

8	BLK	80	MTOR	152	BRAF	224	F2R
9	CCR5	81	LGALS3	153	MDM2	225	CTSS
10	CXCL8	82	KIT	154	IRAK4	226	GSK3B
11	MMP13	83	SRC	155	CHRM3	227	FGFR2
12	TLR4	84	ESR1	156	OPRM1	228	TKT
13	VCAM1	85	XDH	157	HDAC4	229	MB
14	ICAM1	86	IMPDH2	158	MALT1	230	PIM2
15	VEGFA	87	AR	159	TRPV4	231	CTSD
16	TTR	88	EGFR	160	CTSL	232	ALPL
17	ITGAV	89	PIK3CD	161	MAP2K3	233	RPS6KB1
18	MMP9	90	LCK	162	F2	234	HDAC6
19	CCR2	91	MMP7	163	CSK	235	INSR
20	PTPRC	92	PPARG	164	MAPK9	236	FGFR4
21	MMP2	93	PRKCD	165	F13A1	237	HSD11B1
22	SELE	94	AKT1	166	PDGFRB	238	CNR1
23	MAPK14	95	BRD2	167	PTGER2	239	PTGER3
24	ADAM17	96	PTGER4	168	IGFBP3	240	ACHE
25	NOS2	97	ATM	169	PGR	241	HRH2
26	SYK	98	CD38	170	FLT4	242	KCNE1
27	NR3C1	99	TEK	171	TSPO	243	MAPKAPK5
28	DHODH	100	PLAU	172	MAPK13	244	CCND1
29	MMP12	101	P2RX7	173	SHH	245	NTRK2
30	JUN	102	CASP3	174	FKBP5	246	TGFBR1
31	MAPK8	103	CYP2C19	175	BTK	247	MERTK
32	ITGB2	104	TACR1	176	IGF1R	248	DYRK1A
33	MAP3K8	105	CA2	177	MAPK3	249	EGLN1
34	PIK3CG	106	FLT3	178	PRKCA	250	PKM
35	LRRK2	107	ABCC1	179	PDE3A	251	REN
36	HSPA5	108	CASP1	180	MAP2K7	252	C1R
37	MPO	109	RELA	181	JAK1	253	HK2
38	MAPK1	110	AHR	182	ABCG2	254	LTA4H
39	CCR1	111	CYP1A2	183	NTRK1	255	METAP2
40	CX3CR1	112	PRKCB	184	DRD1	256	PSMB1
41	ELANE	113	PARP1	185	BAD	257	DRD3
42	PTGS1	114	MCL1	186	AXL	258	PIK3R1
43	MMP14	115	CDK2	187	LTB4R	259	WEE1
44	TLR9	116	IKBKE	188	ALOX5AP	260	NTRK3
45	CSF1R	117	CYP19A1	189	TYRO3	261	CASR
46	CTSB	118	HSPA1A	190	TACR2	262	SMO
47	MMP8	119	ALOX15	191	QDPR	263	BCHE
48	ALOX5	120	S1PR1	192	GRM5	264	KCNQ1
49	CTSK	121	RHOA	193	PLAA	265	VCP
50	CCR4	122	MET	194	CNR2	266	BRD4
51	ADORA3	123	MAP3K14	195	MTNR1B	267	HSF1

52	ITGAL	124	JAK2	196	FABP4	268	NQO1
53	IKBKB	125	PDE2A	197	PDE4A	269	CA4
54	NFKB1	126	MAPKAPK2	198	CXCR1	270	CETP
55	JAK3	127	FADS1	199	TGM2	271	FAAH
56	PLA2G2A	128	CDK4	200	PTK2B	272	MAP3K11
57	CHUK	129	CYP17A1	201	ADAM10	273	MC4R
58	PLA2G10	130	SLCO1B1	202	TYMP	274	RIPK2
59	DHFR	131	GSR	203	CHRNA7	275	SERPINA6
60	ADAMTS4	132	PNP	204	APP	276	CRHR1
61	ABCB1	133	TIE1	205	EPHB4	277	OPRK1
62	TYMS	134	ADORA2A	206	HDAC2	278	PER2
63	PRKCQ	135	KDR	207	CXCR2	279	OPRD1
64	PLA2G7	136	ERBB2	208	MST1R	280	MBD2
65	CDK6	137	MAPK10	209	MKNK1	281	PTGER1
66	TYK2	138	HSPA8	210	MAP3K5	282	GPBAR1
67	ACE	139	MAP2K4	211	CA3	283	ADAMTS5
68	DPP4	140	IDO1	212	CCR9	284	TOP1
69	FGFR1	141	MAP2K6	213	ALDH2	285	COMT
70	NOS3	142	CASP7	214	SLC29A1	286	PAK1
71	PRKCH	143	HSP90AB1	215	KCNMA1	287	CFD
72	TACR3	144	FLT1	216	RAF1	288	P2RX3

Table S6. Results of KEGG pathway enrichment analysis of ZDS putative targets.

**Pathway ID**	**Pathway name**	**Gene count**	***P* value**	**Gene**
hsa0520	Pathways in cancer	78	3.50E-32	GSK3B, CXCL8, HSP90AB1, FLT3, FLT4, PIK3CD, IGF1R, IKBKB, SHH, CASP7, CCND1, CASP3, AKT1, ITGAV, PIM2, JAK2, JAK3, JAK1, PDGFRB, CHUK, PRKCB, MMP1, MMP2, F2R, PRKCA, F2, MMP9, RHOA, TGFBR1, TGFBR2, AR, SMO, KIT, PPARG, RAF1, IL6ST, MET, PTGER4, CSF1R, HDAC2, PTGER1, HDAC1, GSTP1, PTGER2, PTGER3, PIK3R1, PTGS2, EGFR, RELA, MAPK9, MAPK8, TERT, ERBB2, HMOX1, MAPK1, MAPK3, NTRK1, EGLN1, NQO1, JUN, NOS2, BAD, BRAF, ESR1, MTOR, ESR2, NFKB1, IL2, VEGFA, MAPK10, CDK6, RPS6KB1, CDK4, CDK2, MDM2, FGFR4, FGFR2, FGFR1
hsa04010	MAPK signaling pathway	55	6.61E-27	FLT1, FLT3, FLT4, TNF, IGF1R, IKBKB, CASP3, KDR, AKT1, MAP3K8, MAP3K5, PDGFRB, MAP2K3, MAP2K4, CHUK, PRKCB, PRKCA, IRAK4, TGFBR1, TGFBR2, MAPKAPK2, KIT, MAPKAPK5, RAF1, MET, CSF1R, EGFR, RELA, MAPK9, PAK1, MAPK8,

				MKNK1, ERBB2, MAPK1, MAP2K7, MAPK3, MAP2K6, NTRK1, NTRK2, HSPA8, JUN, INSR, BRAF, MAPK14, NFKB1, MAPK13, VEGFA, MAPK10, TEK, FGFR4, MAP3K14, MAP3K11, FGFR2, FGFR1, HSPA1A
hsa05417	Lipid and atherosclerosis	46	2.35E-25	GSK3B, CXCL8, HSP90AB1, SRC, PIK3CD, PIK3R1, TNF, RELA, ICAM1, IKBKB, MAPK9, CASP7, MAPK8, CASP3, CASP1, AKT1, MAPK1, JAK2, MAP2K7, IKBKE, MAPK3, MAP2K6, MAP3K5, MAP2K3, MAP2K4, HSPA8, JUN, VCAM1, HSPA5, CHUK, MMP1, NOS3, BAD, MMP3, PRKCA, IRAK4, MAPK14, SELE, MMP9, RHOA, NFKB1, MAPK13, MAPK10, PPARG, TLR4, HSPA1A
hsa05161	Hepatitis B	40	2.03E-24	CXCL8, SRC, PIK3CD, PIK3R1, TNF, RELA, IKBKB, MAPK9, MAPK8, CASP3, PTK2B, AKT1, MAPK1, JAK2, MAP2K7, JAK3, IKBKE, JAK1, MAPK3, MAP2K6, MAP2K3, MAP2K4, JUN, CHUK, PRKCB, BAD, PRKCA, BRAF, TYK2, IRAK4, MAPK14, MMP9, TGFBR1, NFKB1, TGFBR2, MAPK13, MAPK10, CDK2, RAF1, TLR4
hsa05418	Fluid shear stress and atherosclerosis	36	1.10E-22	HSP90AB1, SRC, GSTP1, PIK3CD, PLAT, PIK3R1, TNF, RELA, ICAM1, IKBKB, MAPK9, MAPK8, CTSL, KDR, AKT1, HMOX1, ITGAV, MAP2K7, MAP2K6, MAP3K5, NQO1, MAP2K4, JUN, VCAM1, CHUK, NOS3, MMP2, MAPK14, SELE, MMP9, RHOA, NFKB1, MAPK13, VEGFA, MAPK10, TRPV4
hsa05167	Kaposi sarcoma-associated herpesvirus infection	41	2.50E-22	GSK3B, CXCL8, SRC, PIK3CD, PIK3R1, PTGS2, RELA, PIK3CG, ICAM1, IKBKB, MAPK9, MAPK8, CCND1, CASP3, AKT1, MAPK1, JAK2, CCR5, MAP2K7, CCR4, IKBKE, JAK1, MAPK3, MAP2K6, CCR1, MAP2K4, JUN, SYK, CHUK, TYK2, MAPK14, MTOR, NFKB1, MAPK13, VEGFA, MAPK10, CDK6, CDK4, MAPKAPK2, RAF1, IL6ST
hsa04668	TNF signaling pathway	32	2.85E-21	PIK3CD, PIK3R1, PTGS2, TNF, RELA, ICAM1, IKBKB, MAPK9, CASP7, MAPK8, CASP3, AKT1, MAPK1, MAP3K8, MAP2K7, MAPK3, MAP2K6, MAP3K5, MAP2K3, MAP2K4, JUN, VCAM1, CHUK, MMP3, MAPK14, SELE, MMP9, NFKB1, MAPK13, MAPK10, MMP14, MAP3K14

hsa05215	Prostate cancer	30	3.08E-21	GSK3B, HSP90AB1, GSTP1, PIK3CD, PLAT, PIK3R1, RELA, EGFR, IGF1R, IKBKB, CCND1, PLAU, ERBB2, AKT1, MAPK1, MAPK3, PDGFRB, CHUK, BAD, MMP3, BRAF, MMP9, MTOR, NFKB1, AR, CDK2, MDM2, RAF1, FGFR2, FGFR1
hsa04933	AGE-RAGE signaling pathway in diabetic complications	30	8.00E-21	CXCL8, PIK3CD, PIK3R1, TNF, RELA, ICAM1, MAPK9, MAPK8, CCND1, CASP3, AKT1, MAPK1, JAK2, MAPK3, JUN, VCAM1, PRKCB, NOS3, MMP2, PRKCD, PRKCA, MAPK14, SELE, TGFBR1, NFKB1, TGFBR2, MAPK13, VEGFA, MAPK10, CDK4
hsa04625	C-type lectin receptor signaling pathway	30	2.70E-20	SRC, PIK3CD, PIK3R1, PTGS2, TNF, MALT1, RELA, IKBKB, MAPK9, PAK1, MAPK8, CASP1, AKT1, MAPK1, IKBKE, MAPK3, JUN, SYK, CHUK, PRKCD, MAPK14, RHOA, IL2, NFKB1, MAPK13, MAPK10, MAPKAPK2, MDM2, RAF1, MAP3K14
hsa04151	PI3K-Akt signaling pathway	51	7.29E-20	GSK3B, FLT1, HSP90AB1, FLT3, FLT4, PIK3CD, PIK3CG, IGF1R, IKBKB, CCND1, KDR, AKT1, ITGAV, JAK2, JAK3, JAK1, PDGFRB, SYK, CHUK, F2R, PRKCA, KIT, RAF1, MET, TLR4, CSF1R, PIK3R1, EGFR, RELA, ERBB2, MAPK1, MCL1, MAPK3, NTRK1, NTRK2, BAD, NOS3, INSR, MTOR, NFKB1, IL2, VEGFA, CDK6, RPS6KB1, CDK4, CDK2, MDM2, TEK, FGFR4, FGFR2, FGFR1
hsa05212	Pancreatic cancer	26	1.37E-19	PIK3CD, PIK3R1, RELA, EGFR, IKBKB, MAPK9, MAPK8, CCND1, ERBB2, AKT1, MAPK1, JAK1, MAPK3, CHUK, BAD, BRAF, TGFBR1, MTOR, NFKB1, TGFBR2, VEGFA, MAPK10, CDK6, RPS6KB1, CDK4, RAF1
hsa05163	Human cytomegalovirus infection	40	5.74E-19	PTGER4, GSK3B, CXCL8, PTGER1, SRC, PTGER2, PTGER3, PIK3CD, PIK3R1, PTGS2, TNF, RELA, EGFR, IKBKB, CCND1, CASP3, CXCR2, PTK2B, AKT1, MAPK1, ITGAV, CCR5, JAK1, MAPK3, MAP2K6, CCR1, CHUK, PRKCB, PRKCA, MAPK14, RHOA, MTOR, NFKB1, MAPK13, VEGFA, CDK6, RPS6KB1, CDK4, MDM2, RAF1
hsa04620	Toll-like receptor signaling pathway	29	1.07E-18	CXCL8, PIK3CD, PIK3R1, TNF, RELA, IKBKB, MAPK9, MAPK8, CTSK, AKT1, MAPK1, MAP3K8, MAP2K7, IKBKE, JAK1, MAPK3, MAP2K6, MAP2K3, MAP2K4, JUN, CHUK,

				TYK2, IRAK4, MAPK14, NFKB1, MAPK13, MAPK10, TLR9, TLR4
hsa04660	T cell receptor signaling pathway	30	2.60E-18	GSK3B, PIK3CD, PIK3R1, TNF, MALT1, RELA, IKBKB, MAPK9, PAK1, MAPK8, AKT1, MAPK1, MAP3K8, MAP2K7, MAPK3, JUN, CHUK, MAPK14, RHOA, IL2, NFKB1, MAPK13, MAPK10, ZAP70, PTPRC, LCK, CDK4, PRKCQ, RAF1, MAP3K14
hsa01521	EGFR tyrosine kinase inhibitor resistance	25	6.16E-18	GSK3B, SRC, PIK3CD, PIK3R1, EGFR, IGF1R, ERBB2, KDR, AKT1, MAPK1, JAK2, JAK1, MAPK3, PDGFRB, PRKCB, BAD, PRKCA, BRAF, MTOR, VEGFA, RPS6KB1, AXL, RAF1, MET, FGFR2
hsa05135	Yersinia infection	31	1.00E-17	GSK3B, CXCL8, SRC, PIK3CD, PIK3R1, TNF, RELA, IKBKB, MAPK9, MAPK8, CASP1, PTK2B, AKT1, MAPK1, MAP2K7, MAPK3, MAP2K6, MAP2K3, MAP2K4, JUN, CHUK, IRAK4, MAPK14, RHOA, IL2, NFKB1, MAPK13, MAPK10, ZAP70, LCK, TLR4
hsa01522	Endocrine resistance	27	1.04E-17	SRC, PIK3CD, PIK3R1, EGFR, IGF1R, MAPK9, MAPK8, CCND1, ERBB2, AKT1, MAPK1, MAPK3, JUN, BAD, MMP2, BRAF, MAPK14, MMP9, ESR1, MTOR, ESR2, MAPK13, MAPK10, RPS6KB1, CDK4, MDM2, RAF1
hsa04014	Ras signaling pathway	39	2.24E-17	CSF1R, FLT1, FLT3, FLT4, PIK3CD, PIK3R1, RELA, EGFR, IGF1R, IKBKB, MAPK9, PAK1, MAPK8, KDR, AKT1, MAPK1, MAPK3, PDGFRB, NTRK1, NTRK2, CHUK, PRKCB, BAD, INSR, PLA2G2A, PRKCA, RHOA, NFKB1, VEGFA, MAPK10, ZAP70, PLA2G10, KIT, TEK, RAF1, FGFR4, MET, FGFR2, FGFR1
hsa05145	Toxoplasmosis	28	2.69E-17	TNF, RELA, PIK3CG, IKBKB, MAPK9, MAPK8, CASP3, ALOX5, AKT1, MAPK1, JAK2, CCR5, JAK1, MAPK3, MAP2K6, MAP2K3, HSPA8, NOS2, CHUK, BAD, TYK2, IRAK4, MAPK14, NFKB1, MAPK13, MAPK10, TLR4, HSPA1A

Table S7. Cell viability of HFLS-RA.

Compound	Cell viability (%, mean±SD, n=3)			
	**25 μM**	**50 μM**	**75 μM**	**100 μM**
**1**	96.2±2.08	93.5±2.66	74.1±0.96	56.9±1.06
**2**	94.9±2.70	93.04±2.09	74.7±1.58	50.0±1.62
**4**	97.7±0.99	92.9±2.19	81.6±0.79	76.1±1.35
**5**	98.2±2.28	95.8±2.39	84.8±1.47	49.2±0.64
**6**	97.9±1.78	96.1±2.19	90.46±0.88	79.6±1.34
**7**	96.9±1.25	96.1±2.19	86.8±0.60	84.1±1.02
**8**	99.8±1.11	96.1±1.52	87.5±1.44	73.6±0.64

Table S8. Cell proliferation viability of LPS induced HFLS-RA.

Compound	Cell viability (%, mean±SD, n=3)	
	**25 μM**	**50 μM**
**1**	97.9±0.78	92.5±1.09
**2**	108.4±2.45	97.6±1.75
**4**	105.8±2.28	97.2±1.51
**5**	97.6±1.78	94.6±1.43
**6**	109.6±1.93	102.2±2.84
**7**	99.7±0.95	95.1±1.35
**8**	106.1±1.47	97.9±1.15
**LPS (1μg/mL)**	115.5±0.88	

Table S9. Effects of compounds 1, 5 and 7 on the levels of pro-inflammatory cytokines IL-1β.

Concentration	IL-1β (pg/mL, mean±SD, n=3)			
**(μM)**	**1**	**5**	**7**	**DEX**
**50**	1042±62.7	1161±55.6	1116±55.1	1013±27.4
**25**	1166±75.7	1264±26.4	1438±45.2	1164±63.8
**12.5**	1204±32.2	1294±22.7	1530±9.39	1322±21.1
**0**	371±13.6			
**LPS (1μg/mL)**	1740±16.7			

Table S10. Effects of compounds 1, 5 and 7 on the levels of pro-inflammatory cytokines IL-6.

Concentration	IL-6 (pg/mL, mean±SD, n=3)			
(μM)	1	5	7	DEX
50	851±18.7	905±3.02	583±45.4	207±6.66
25	941±5.94	944±12.3	697±10.4	214±5.67
12.5	962±8.88	967±10.4	729±22.5	221±7.26
0	243±8.88			
LPS (1μg/mL)	1055±12.9			

Table S11. Effects of compounds 1, 5 and 7 on the levels of pro-inflammatory cytokines TNF-α.

Concentration	TNF-α (pg/mL, mean±SD, n=3)			
(μM)	1	5	7	DEX
50	894±34.9	970±38.8	633±8.83	609±8.44
25	1013±42.2	1031±7.33	951±7.14	742±21.0
12.5	1055±16.6	1073±4.92	1062±24.3	818±6.98
0	433±14.0			
LPS (1μg/mL)	1114±11.0			
